# In-plane dielectric constant and conductivity of confined water

**DOI:** 10.1038/s41586-025-09558-y

**Published:** 2025-10-15

**Authors:** R. Wang, M. Souilamas, A. Esfandiar, R. Fabregas, S. Benaglia, H. Nevison-Andrews, Q. Yang, J. Normansell, P. Ares, G. Ferrari, A. Principi, A. K. Geim, L. Fumagalli

**Affiliations:** 1 Department of Physics and Astronomy, University of Manchester, Manchester, UK; 2 National Graphene Institute, University of Manchester, Manchester, UK; 3 Department of Physics, Politecnico di Milano, Milan, Italy; 4 Present Address: Department of Molecular Spectroscopy, Max Planck Institute for Polymer Research, Mainz, Germany; 5 Present Address: Departamento de Matemática Aplicada and Modeling Nature Research Unit, Facultad de Ciencias, Universidad de Granada, Granada, Spain; 6 Present Address: Departamento de Física de la Materia Condensada and Condensed Matter Physics Center (IFIMAC), Universidad Autónoma de Madrid, Madrid, Spain

**Keywords:** Nanofluidics, Surfaces, interfaces and thin films, Imaging techniques, Electronic properties and materials, Chemical physics

## Abstract

Water is essential for almost every aspect of life on our planet and, unsurprisingly, its properties have been studied in great detail^1^. However, disproportionately little remains known about the electrical properties of interfacial and strongly confined water^2,3^, in which the structure deviates from that of bulk water, becoming distinctly layered^4,5^. The structural change is expected to affect the conductivity of water and particularly its polarizability, which in turn modifies intermolecular forces that play a crucial role in many physical and chemical processes^6–9^. Here we use scanning dielectric microscopy (SDM)^10^ to probe the in-plane electrical properties of water confined between atomically flat surfaces separated by distances down to 1 nm. For confinement exceeding several nanometres, water exhibits an in-plane dielectric constant close to that of bulk water and its proton conductivity is notably enhanced, gradually increasing with decreasing water thickness. This trend abruptly changes when the confined water becomes only a few molecules thick. Its in-plane dielectric constant reaches large, ferroelectric-like values of about 1,000, whereas the conductivity peaks at several S m^−1^, close to values characteristic of superionic liquids. We attribute the enhancement to strongly disordered hydrogen bonding induced by the few-layer confinement, which facilitates both easier in-plane polarization of molecular dipoles and faster proton exchange. This insight into the electrical properties of nanoconfined water is important for understanding many phenomena that occur at aqueous interfaces and in nanoscale pores.

## Abstract (web summary)

Scanning dielectric microscopy of nanocapillaries filled with water reveals that interfacial and strongly confined water exhibits a large in-plane dielectric constant and an in-plane conductivity approaching superionic values.

## Main

In the bulk and at room temperature (RT), water exhibits exceptionally high dielectric constant (*ε*_bulk_ ≈ 80) and high (for a wide-bandgap insulator) electrical conductivity (*σ*_bulk_ ≈ 10^−5^ S m^−1^)^1^. Both characteristics are inherently connected with the ability of water molecules to form hydrogen bonds^11–13^ and are key to the main properties of water. Among them is its remarkable ability to dissolve more substances than any other liquid^7,8^, which originates from the high *ε*_bulk_ of water that efficiently suppresses Coulomb interactions between solutes. The strong dielectric screening is also critical in biochemical processes responsible for life^7^ (for example, proteins folding and assembly, their interaction with nucleic acids and ion transport across cell membranes). Notably, several phenomena involving water (including solvation) occurs at solid interfaces, at which liquid water exhibits a distinct layered structure^4,5^. Within these layers, the hydrogen-bond network is greatly altered compared with that of bulk water, no longer following the ice rules^14^. Accordingly, the electrical properties of water near surfaces and inside nanoscale cavities are expected to be different from those of bulk water^2,3^. These differences have been the subject of intense research. In particular, proton conductivity of near-surface water has been reported to become much larger than *σ*_bulk_, although the magnitude of this enhancement and the underlying mechanism remain debated^15–19^. Meanwhile, it has proved difficult to assess the dielectric properties of interfacial and nanoconfined water^20,21^. Only recently^22^ has it been shown that, in the direction perpendicular to surfaces, water within several near-surface layers becomes non-polarizable, exhibiting the dielectric constant *ε*_⊥_ ≈ 2, in agreement with many theoretical predictions^23–26^. However, the in-plane dielectric constant *ε*_//_ of interfacial water remains essentially unknown, because *ε*_//_ is even more challenging to measure than *ε*_⊥_ owing to the lack of suitable experimental techniques. The in-plane polarizability of near-surface water is also poorly understood theoretically and, depending on assumptions and confinement strength, *ε*_//_ was predicted to attain values from giant to bulk-like to exceptionally small^27–30^.

## SDM on water-filled nanochannels

**Fig. 1:**
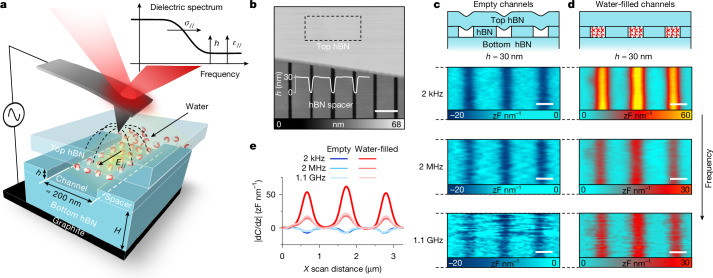
Broadband dielectric imaging of nanoconfined water. **a**, Schematic of the experimental set-up and our devices. The hBN top, bottom and spacer layers are shown in light blue and the graphite ground electrode is shown in black. The water is filled from a back-side inlet (Extended Data Fig. 1d). Oscillating voltage (*f* from 0.1 kHz to 1.1 GHz) is applied to the AFM tip and the measured local impedance is converted into dielectric spectra such as that shown schematically in the inset. Relatively thick bottom hBN (*H* ≈ 50–200 nm) is used to create in-plane field *E*_//_ inside water channels. **b**, AFM micrograph of a device with *h* ≈ 30 nm, with the inset showing a trace over the exposed spacers. **c**,**d**, Dielectric images at characteristic *f* of 2 kHz, 2 MHz and 1.1 GHz for the same three channels before and after filling them with water, respectively. The images were taken at 295 ± 1 K from the area outlined in **b** by the dashed rectangle. The top panels in **c** and **d** sketch the cross-section of the channels (not to scale). The slight sagging of the top hBN into empty channels disappears after filling them with water. The sagging was monitored by AFM to ensure that the channels were fully filled^22^. **e**, Dielectric profiles (averaged over ten lines) from **c** and **d**, colour-coded. |d*C*/d*z*| is defined in Methods. Scale bars: 1 μm (**b**); 500 nm (**c**,**d**). Source data

**Extended Data Fig. 1:**
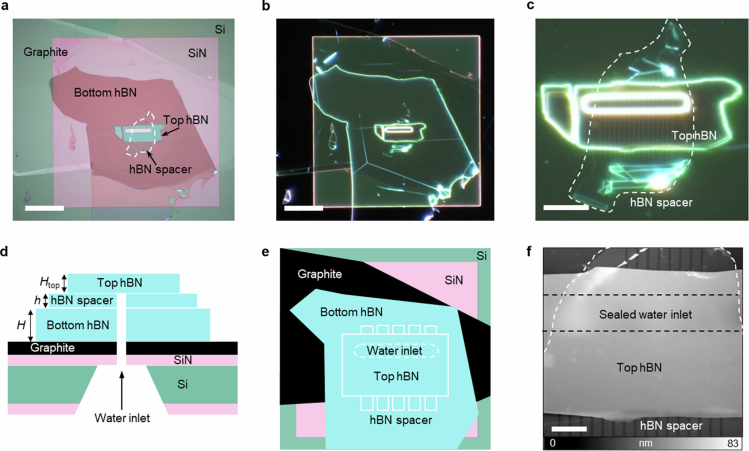
Devices for in-plane dielectric imaging of confined water. **a**, Optical image of a representative device with channel height *h* ≈ 5 nm. The edges of the spacer layer are indicated by the white dashed lines. The large square region (pink) is the suspended SiN membrane. It has an approximately 20-μm-long rectangular slit in its centre (white) that connects nanochannels to a water reservoir at the back side of the device. **b**, Dark-field optical image of the same device. **c**, Zoom into its central region in which numerous parallel channels can be seen. **d**,**e**, Sketch of the device geometry: its cross-section (**d**) and top view (**e**) (not to scale). **f**, AFM topography image of the central region of the device. The black dashed lines indicate the position of the aperture. The white dashed curve indicates the edges of the hBN spacer crystal. Scale bars, 20 μm (**a**,**b**); 10 μm (**c**); 3 μm (**f**).

To examine the in-plane response of nanoconfined water, we have used SDM^10^. Our set-up is shown schematically in Fig. 1a and detailed in Methods. Briefly, nanoscale channels of various heights *h* and width of about 200 nm were fabricated by van der Waals assembly of atomically flat monocrystals of hexagonal boron nitride (hBN) and graphite (for fabrication details, see Methods). An AC voltage was applied to an atomic force microscope (AFM) tip and its mechanical response to the electrostatic force translated into local electrical impedance. By scanning the tip across the channels, the dielectric response of water inside the nanochannels was measured and compared with that of nearby insulating spacers made of hBN with known dielectric constant (*ε*_hBN_ ≈ 4). This approach is conceptually similar to that used to study *ε*_⊥_ of nanoconfined water^22^ but the previous set-up was insensitive to *ε*_//_ because the electric field *E* was applied perpendicular to the channels. To measure *ε*_//_, we have introduced two main changes. First, the ground electrode (graphite crystal in Fig. 1a and Extended Data Fig. 1) has been moved away from the investigated water layer by adding a relatively thick hBN crystal to the assembly, which creates an in-plane field component *E*_//_ inside the water (Fig. 1a). Second, we have expanded the measurement bandwidth by about five orders of magnitude from the standard kHz frequencies into the GHz regime, which is challenging but essential for measuring *ε*_//_ in the presence of unexpectedly high *σ*_//_ found for few-layer (quasi-2D) water (see below).

**Fig. 2:**
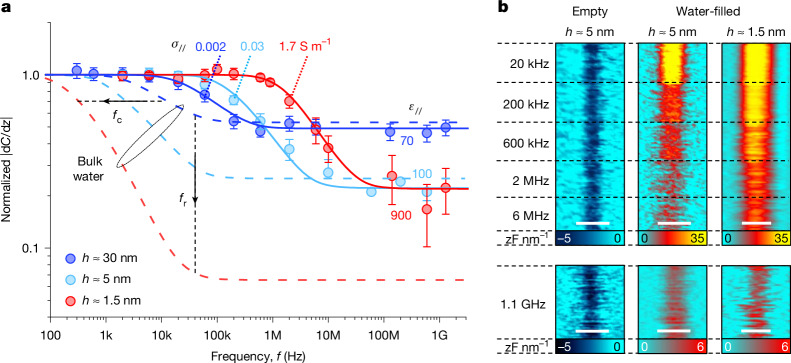
Dielectric dispersion of nanoconfined water. **a**, Examples of the dielectric spectra for three representative *h*. The colour-coded symbols are the peak values for *|*d*C*/d*z|* curves as in Fig. 1e, normalized by their magnitude at low *f*. Spectra before normalization are shown in Extended Data Fig. 10. The error bars indicate the random noise level as measured at each *f*. Solid curves, best fits using the numerical simulations. The fits match *f*_c_ (yielding *σ*_//_) and the *y*-axis position of the high-*f* plateau (yielding *ε*_//_). Dashed curves, same simulations for bulk water (*ε*_bulk_ = 80 and *σ*_bulk_ = 2 × 10^−4^ S m^−1^; see Methods). The black lines show the evolution of *f*_c_ and *f*_r_ with decreasing *h*. **b**, Dielectric images (|d*C*/d*z|* in units of zF nm^−1^) taken for individual channels with *h* ≈ 1.5 and 5 nm (empty and filled with water), as specified in the legends. Upper panels, images for a fixed-*y* position of the AFM tip; about 25 lines were recorded at each *f*. Lower panels, images at 1.1 GHz; about 100 lines are shown for clarity because of higher noise. Scale bars, 500 nm. Source data

**Extended Data Fig. 2:**
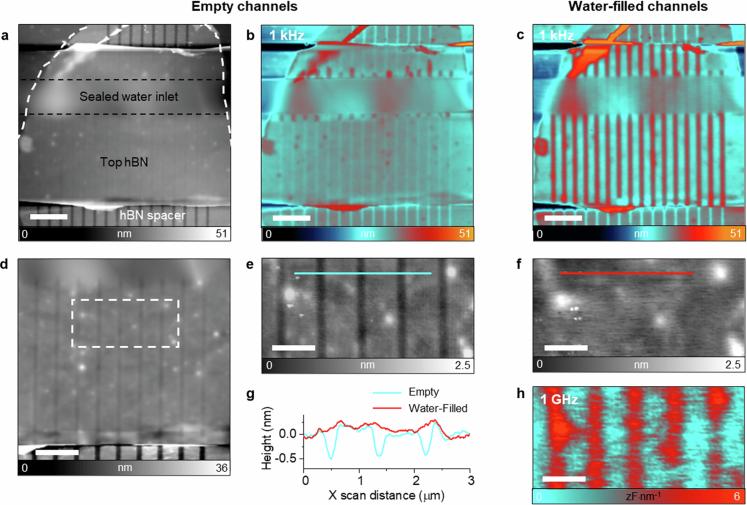
Filling nanochannels with water. **a**, AFM topography image of a representative device with *h* ≈ 5 nm before filling it with water—same image as in Extended Data Fig. 1f but using a flattening filter to better visualize the sagging of the top hBN layer into nanochannels. The image was taken in the intermittent-contact attractive mode with no voltage applied. **b**,**c**, Same as **a** but with applying a low-*f* electric field (*v*_AC_ = 8 V, 1 kHz) before (**b**) and after (**c**) filling the nanochannels with water. The contrast above the channels changes from light cyan in **b** to red in **c**, indicating successful filling of water. This contrast in **c**, which shows an apparent roughness change of several nanometres, arises from the large in-plane conductivity of water and not from actual topographical features. The top surface of the device becomes essentially atomically flat after water filling, with residual roughness <3 Å for all of our devices, as demonstrated in **f** and **g** (see also the ‘Device fabrication’ section in Methods). **d**, Zoom-in of the central region in **a**. The top hBN layer is partially sagged into the empty channels. **e**, Further zoom-in showing the region indicated by the dashed rectangle in **d**. **f**, Same as **e** but after filling nanochannels with water. It shows that the top hBN layer became flat, no longer sagging inside the nanochannels. **g**, Topography profiles over three channels in **e** and **f**, as indicated by colour-coded lines. **h**, Dielectric image taken from the same region as in **f** at a constant height and at 1 GHz. The plotted values of *|*d*C*/d*z*| are variations after subtracting the *|*d*C*/d*z*| values found at the centre of the hBN spacer region. Scale bars: 3 μm (**a**–**c**); 2 μm (**d**), 1 μm (**e**,**f**,**h**). Source data

**Extended Data Fig. 3:**
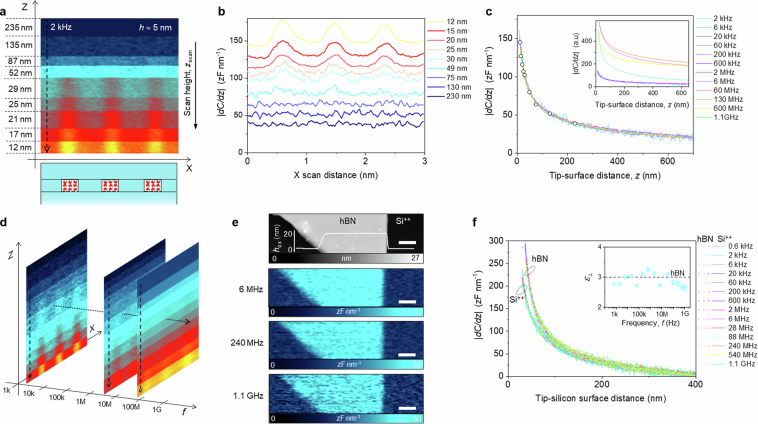
Measurement and calibration procedures for local broadband dielectric spectroscopy. **a**, Representative *|*d*C*/d*z*|(*x*,*z*) image taken at 2 kHz for channels with *h* ≈ 5 nm. The image shows three nanochannels and was acquired by measuring *|*d*C*/d*z|* at various constant heights and a fixed *y* position. The tip approached the surface from above using small steps. **b**, Absolute values of *|*d*C*/d*z|* for scanning along the lateral (*x*) axis at constant *y* and various heights. The profiles were averaged over about ten lines using images in **a**. The signal became sensitive to the presence of water inside the channels at *z* ≲ 30 nm. **c**, Gain-calibrated *|*d*C*/d*z*|(*z*) curves at different frequencies taken over the centre of the spacer region (colour-coded lines). White symbols indicate the values of *|*d*C*/d*z|* and *z*_scan_ corresponding to the profiles shown in **b**. Inset, same curves without calibration of the electrical gain *g* and the offsets (colour-coded). We used the curve at 2 kHz as a reference to calibrate *g* at high frequencies by matching the reference curve with the curves taken at higher *f*, using only *g* and an offset as fitting parameters. **d**, Schematic representation of our 3D AFM datasets *|*d*C*/d*z*|(*x*,*z*,*f*) obtained by acquiring subsequent images *|*d*C*/d*z*|(*x*,*z*) for many frequencies from kHz to GHz. The black dashed arrows in **a** and **d** indicate the position at which the *|*d*C*/d*z*|(*z*) curves in **c** were acquired before taking such 3D images. **e**,**f**, Example of broadband dielectric imaging spectroscopy on a test sample of hBN on doped silicon using the same *g* calibration procedure: topography image (grey coloured) of 21-nm-thick hBN crystal on doped Si and corresponding calibrated dielectric images (cyan-coloured images) at frequencies 6 MHz, 240 MHz and 1.1 GHz at a constant height (about 45 nm). The plotted values of *|*d*C*/d*z*| are variations after subtracting the *|*d*C*/d*z*| values measured on the Si substrate. Scale bars, 1 μm. The curves in **f** are gain-calibrated *|*d*C*/d*z*|(*z*) curves taken above the Si substrate (solid curves) and the hBN layer (dotted) at various *f*. The gain *g* for each *f* was obtained by scaling the curves measured on doped Si to the low-*f* reference curve at 2 kHz using only *g* and an offset as fitting parameters. By fitting the calibrated curves on hBN using the procedure and analytical formula in ref. ^72^, we obtained the dielectric constant of the hBN layer as a function of *f* (inset). Its average value across all of the *f* up to GHz was found to be *ε*_⊥hBN_ = 2.9 ± 0.2, which is in good agreement with the expected bulk value for hBN in the perpendicular direction (*ε*_⊥hBN_ ≈ 3, dashed line). Bars, standard error defined as 95% confidence interval of the fitting, not shown when smaller than the symbol. Other fitting parameters: AFM tip *R* = 102 nm and *θ* = 25°, obtained by fitting the curve taken on doped Si at 2 kHz. Source data

Representative SDM images from our relatively large (reference) channels with *h* ≈ 30 nm (Fig. 1b) are shown in Fig. 1c,d before and after filling them with deionized water, respectively (see also Extended Data Fig. 2). The dielectric contrast between empty channels (*ε* = 1) and hBN spacers is negative as expected, reflecting the relatively larger dielectric constant of hBN. After the channels were filled, the contrast changed and became strongly positive, confirming the presence of water inside the channels and its strong dielectric response. With increasing measurement frequency, *f*, the contrast for empty channels did not change, again as expected (Fig. 1c). For water-filled channels, it remained positive with increasing *f* from kHz to MHz and GHz but weakened (Fig. 1d). For each *f*, we then plotted the average contrast of water with respect to hBN (Fig. 1e) and, by repeating the imaging at many different *f* (see detailed measurement and calibration procedures in Methods; Extended Data Fig. 3), we obtained dielectric dispersion curves such as those shown in Fig. 2a (see the blue symbols for *h* ≈ 30 nm).

**Extended Data Fig. 4:**
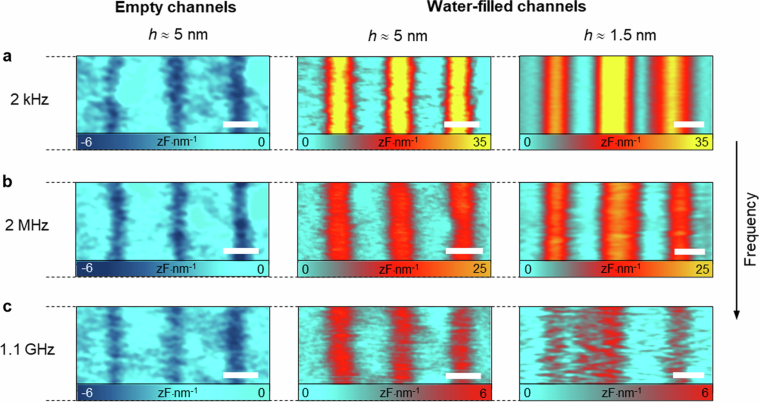
Further dielectric images. **a**–**c**, Dielectric images of three channels taken at constant height and at characteristic *f* of 2 kHz, 2 MHz and 1.1 GHz, respectively. The plotted values of *|*d*C*/d*z*| are variations after subtracting the *|*d*C*/d*z*| values found at the centre of the hBN spacer region. Same devices as in Fig. 2b: *h* ≈ 5 and 1.5 nm, empty and filled with water (as specified in the legends). Scale bars, 500 nm.

**Extended Data Fig. 5:**
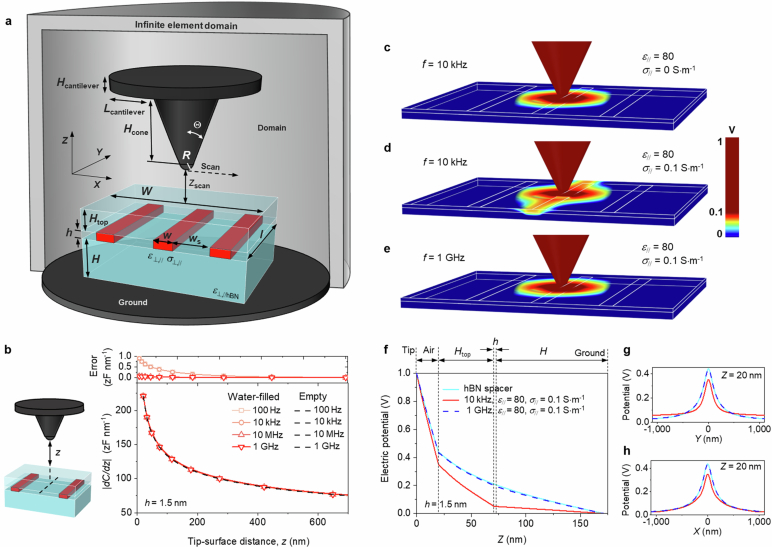
3D numerical simulations. **a**, Schematic (not to scale) of the full-3D numerical model used to fit the experimental data. **b**, Calculated absolute value of *|*d*C*/d*z|* versus tip–surface distance *z* above the centre of the hBN spacer for empty channels (black dashed line) and bulk-water-filled channels (red-coloured symbols) at various frequencies. Channel thickness: *h* = 1.5 nm. Water channel parameters: *ε*_//_ = 80, *σ*_//_ = 0.1 S m^−1^, *ε*_⊥_ = 2, *σ*_⊥_ = 0, *l* = 3 μm, *w*_c_ = 200 nm, *w*_s_ = 800 nm. Upper plot, difference (‘Error’) between calculated curves for water-filled channels and those for empty channels. **c**–**e**, Corresponding calculated electric potential for three representative cases: low frequency (10 kHz) and purely dielectric channels (*ε*_//_ = 80 and *σ*_//_ = 0) (**c**); same low frequency but conductive channels (*ε*_//_ = 80 and *σ*_//_ = 0.1 S m^−1^) (**d**). **e**, Same as **d** but at *f* beyond conductivity relaxation. **f**, Potential along the *Z* direction (counted from the AFM tip) for the tip placed at *z*_scan_ = 20 nm above the centre of the water channel (red and dashed blue lines) and above the centre of the spacer region (cyan line). Frequencies 10 kHz and 1 GHz, colour-coded. **g**, Same as **f** but along the *Y* direction at *Z* = 20 nm measured from the AFM tip (over the top layer). **h**, Same as **g** but along the *X* direction. Other parameters: *R* = 100 nm, *θ* = 25°, *H*_cone_ = 6 μm, *H*_cantilever_ = 3 μm, *L*_cantilever_ = 0 μm, *H*_top_ = 50 nm, *H* = 100 nm, *ε*_//hBN_ = 5, *ε*_⊥hBN_ = 3. Source data

**Extended Data Fig. 6:**
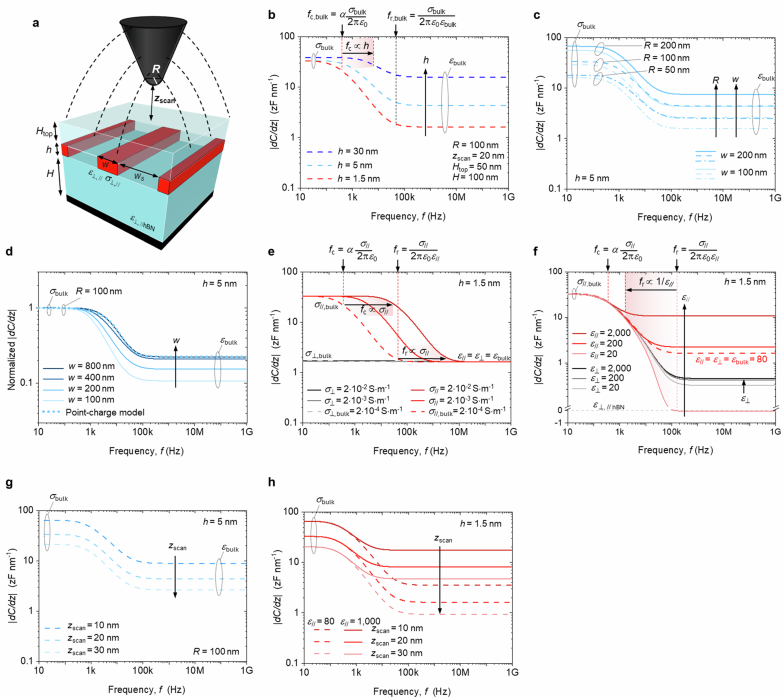
Calculated dielectric spectra using the 3D numerical simulations. **a**, Simplified illustration of our numerical model (shown in full in Extended Data Fig. 5). **b**, Calculated *|*d*C*/d*z|* as a function of frequency for different channel thicknesses (*h* = 30, 5 and 1.5 nm), bulk water (*ε*_//_ = *ε*_⊥_ = *ε*_bulk_ = 80, *σ*_//_ = *σ*_⊥_ = *σ*_bulk_ = 2 × 10^−4^ S m^−1^) inside the channels and scan height *z*_scan_ = 20 nm. **c**, Same as in **b** for *h* = 5 nm but using various AFM tip radii and different channel widths. **d**, Same as in **b** and **c** but for several *w*; normalized by the height of the low-*f* plateau. Dotted curve, corresponding calculations using our point-charge analytical model. **e**, Same as in **b** but for *h* = 1.5 nm and different water *σ*_//_ (red-coloured curves, keeping *σ*_⊥_ = 0) and different *σ*_⊥_ (grey-coloured curves, *σ*_//_ = 0). **f**, Same as in **e** but for different *ε*_//_ (red-coloured curves, keeping *ε*_⊥_ = 2) and different *ε*_⊥_ (grey-coloured curves, *ε*_//_ = 2). The red dashed curve is the case of the channel filled with bulk water, the same as shown in **b**. **g**, Same as in **c** but for several *z*_scan_. **h**, Same as in **g** but for *h* = 1.5 nm and different *ε*_//_ (keeping *ε*_⊥_ = 80). Other parameters used in **b**–**h** (unless stated otherwise in the legends): *R* = 100 nm, *θ* = 25°, *H*_cone_ = 6 μm, *H*_cantilever_ = 3 μm, *L*_cantilever_ = 0 μm, *z*_scan_ = 20 nm, *l* = 3 μm, *w*_c_ = 200 nm, *w*_s_ = 800 nm, *H*_top_ = 50 nm and *H* = 100 nm. All of the plotted *|*d*C*/d*z*| curves are after subtracting the *|*d*C*/d*z*| values found at the centre of the hBN spacer region. Source data

**Extended Data Fig. 7:**
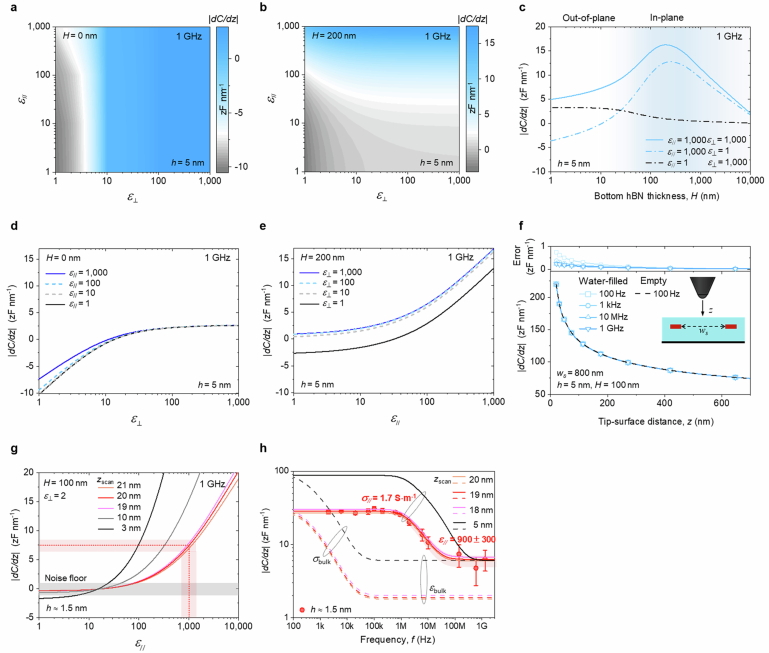
Sensitivity to the in-plane and out-of-plane dielectric constants of water and calibration curves using the 3D numerical simulations. **a**,**b**, Calculated *|*d*C*/d*z|* at 1 GHz as a function of the *ε*_//_ and *ε*_⊥_ of water (with *σ*_//_ = *σ*_⊥_ = *σ*_bulk_ = 2 × 10^−4^ S m^−1^) at scan height *z*_scan_ = 20 nm. *h* = 5 nm and the bottom hBN thicknesses *H* = 0 (metallic substrate) (**a**) and *H* = 200 nm (**b**). **c**, Calculated *|*d*C*/d*z|* as in **a** and **b** but as a function of *H* for different combinations of *ε*_//_ and *ε*_⊥_. AFM tip cone height *H*_cone_ = 12 μm and cantilever length *L*_cantilever_ = 20 μm (nominal parameters) to include long-range range effects that play a role for very thick *H*. **d**, Cross-sections from **a** as a function of the *ε*_⊥_ of water for different values of *ε*_//_. **e**, Cross-sections from **b** as a function of *ε*_//_ for different *ε*_⊥_. **f**, Calculated absolute value of *|*d*C*/d*z|* versus tip–surface distance *z* above the centre of the hBN spacer, as illustrated in the inset, for empty channels (black dashed line) and bulk-water-filled channels (cyan-coloured symbols) at various frequencies. The channel thickness is *h* = 5 nm and the bottom hBN thickness is *H* = 100 nm. Upper panel, error between the calculated curves for water-filled channels and for empty channels. **g**, Same as in **e** but for *h* = 1.5 nm and different *z*_scan_. The dotted red line corresponds to the typical *|*d*C*/d*z|* value on our smallest channels yielding *ε*_//_ ≈ 1,000 at *z*_scan_ estimated to be about 20 nm. The light-shaded region indicates the typical experimental uncertainty in *|*d*C*/d*z|* of about 1 zF nm^−1^. Other parameters used in **a**–**g** (unless stated otherwise in the legends): *R* = 100 nm, *θ* = 25°, *H*_cone_ = 6 μm, *H*_cantilever_ = 3 μm, *L*_cantilever_ = 0 μm, *z*_scan_ = 20 nm, *l* = 3 μm, *w*_c_ = 200 nm, *w*_s_ = 800 nm, *H*_top_ = 50 nm. **h**, Calculated dielectric spectra (lines) corresponding to the experimental data (symbols) for our smallest device in Extended Data Fig. 10 (and in Fig. 2 before normalization) for different scan heights. Bars indicate the random noise level as measured at each *f*. The red solid curve is the best fit with the *ε*_//_ and *σ*_//_ of water as the only fitting parameters (values shown as labels) and the experimentally estimated *z*_scan_ = 19 nm (other experimental and simulated parameters are the same as in Extended Data Fig. 10). The light-shaded region indicates the uncertainty in the extracted values of *ε*_//_ and *σ*_//_. The red dashed curves indicate 3D numerical simulations for the same device assuming the properties of bulk water (*ε*_bulk_ = 80, *σ*_bulk_ = 2 × 10^−4^ S m^−1^). The pink and orange curves are calculated values for *z*_scan_ = 18 and 20 nm, respectively, corresponding to the experimentally estimated uncertainty on *z*_scan_ of ±1 nm. The black curves are the 3D simulations for the dielectric constant of bulk water using *z*_scan_ as the fitting parameter to match the high-frequency plateau, yielding *z*_scan_ = 5 nm, with *σ*_//_ = *σ*_bulk_ (black dashed line) and *σ*_//_  = 1.7 S m^−1^ (black solid line). All of the plotted *|*d*C*/d*z*| curves in panels **a**–**e**, **g** and **h** are after subtracting the *|*d*C*/d*z*| values at the centre of the hBN spacer region. Source data

**Extended Data Fig. 8:**
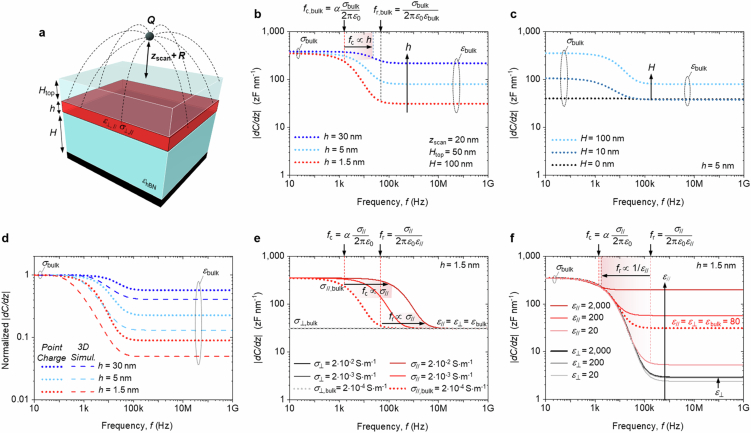
Calculated dielectric spectra using the point-charge analytical model. **a**, Its schematic. A point charge is used to approximate the AFM tip. The device is modelled as a water layer of infinite lateral size (red), which is encapsulated between top and bottom hBN layers (blue). For simplicity, the dielectric constant of hBN is assumed to be isotropic and equal to 4. **b**, Calculated *|*d*C*/d*z|* as a function of frequency for *h* = 30, 5 and 1.5 nm (colour-coded) and bulk water (*ε*_//_ = *ε*_⊥_ = *ε*_bulk_ = 80, *σ*_//_ = *σ*_⊥_ = *σ*_bulk_ = 2 × 10^−4^ S m^−1^). **c**, Same as in **b** for *h* = 5 nm but using various thicknesses of bottom hBN. **d**, The dotted curves are the same as in **b** but normalized with respect to the height of the low-*f* plateau. Dashed curves, corresponding analysis using the full-3D numerical simulations. **e**, Same as in **b** for *h* = 1.5 nm and different *σ*_//_ (red curves, keeping *σ*_⊥_ = 0) and *σ*_⊥_ (grey, *σ*_//_ = 0). **f**, Same as in **b** for *h* = 1.5 nm and various *ε*_//_ (red solid curves, keeping *ε*_⊥_ = 2) and various *ε*_⊥_ (grey curves, *ε*_//_ = 2). The red dotted curve is for the same channel filled with bulk water (also shown in **b**). Other parameters used in **b**–**f** (unless stated otherwise in the legends) are *z*_scan_ = 20 nm, *H*_top_ = 50 nm and *H* = 100 nm. All of the plotted *|*d*C*/d*z*| curves are after subtracting the *|*d*C*/d*z*| values found for the same geometry but with hBN instead of the water layer. Source data

**Extended Data Fig. 9:**
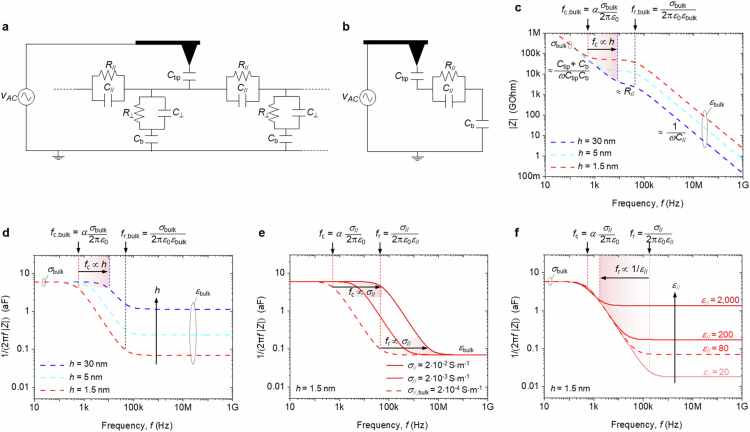
Simulated dielectric spectra using equivalent electrical circuits. **a**, Approximate impedance model for our measurement geometry using a distributed RC network that includes both in-plane and out-of-plane elements to model the water channel. **b**, Simplified in-plane equivalent circuit that takes into account the negligible contribution of the out-of-plane impedance. **c**, Calculated impedance using different channel thicknesses (*h* = 30, 5 and 1.5 nm) and properties of bulk water. **d**, Same as in **c** but plotting the effective capacitance, 1/(|*Z*|2π*f*). **e**, Same as in **d** for *h* = 1.5 nm using different *σ*_//_. **f**, Same as in **e** but for different *ε*_//_. Other parameters used in simulations (**c**–**f**): *R* = 100 nm, *θ* = 25°, *z*_scan_ = 20 nm, l* = 3 μm, *w*_c_ = 200 nm, *H*_top_ = 50 nm and *H* = 100 nm. Source data

**Extended Data Fig. 10:**
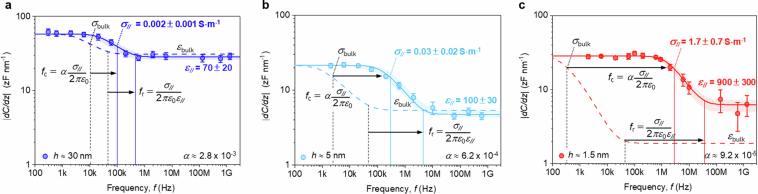
Fitting experimental spectra. **a**–**c**, Symbols are the experimental data of Fig. 2a before normalization. Bars are the random noise level as measured at each *f*. The solid curves are the best fits using the 3D numerical model with the *ε*_//_ and *σ*_//_ of water as the only fitting parameters (labels). The light-shaded regions indicate the uncertainty in the extracted values of *ε*_//_ and *σ*_//_, respectively given by the standard deviation of the mean high-*f* plateau and the standard error from fitting the spectra cut-off frequency. Dashed curves, 3D numerical simulations for the same devices assuming properties of bulk water (*ε*_//_ = *ε*_⊥_ = *ε*_bulk_ = 80, *σ*_//_ = *σ*_⊥_ = *σ*_bulk_ = 2 × 10^−4^ S m^−1^). The vertical lines indicate calculated values of *f*_c_ and *f*_r_ for the case of bulk water (black dashed) and the experimentally extracted water parameters (coloured solid). To evaluate the frequencies, we used equations (5) and (6) and *α* obtained from the full-3D numerical simulations: *α* = 2.8 × 10^−3^ (**a**), *α* = 6.2 × 10^−4^ (**b**) and *α* = 9.2 × 10^−5^ (**c**). *ε*_⊥_ is set to the values measured in ref. ^22^. Other experimental parameters used in the simulations: *h* = 30 nm, *ε*_⊥_ = 32, *σ*_⊥_ = 2 × 10^−4^ S m^−1^, *R* = 84 nm, *θ* = 26°, *H*_top_ = 21 nm, *H* = 53 nm, *w* = 210 nm, *z*_scan_ = 19 nm (**a**); *h* = 5 nm, *ε*_⊥_ = 6.5, *σ*_⊥_ = 2 × 10^−4^ S m^−1^, *R* = 62 nm, *θ* = 17°, *H*_top_ = 34 nm, *H* = 45 nm; *w* = 220 nm, *z*_scan_ = 18 nm (**b**); *h* = 1.5 nm, *ε*_⊥_ = 2.1, *σ*_⊥_ = 2 × 10^−4^ S m^−1^, *R* = 91 nm, *θ* = 27°, *H*_top_ = 50 nm, *H* = 76 nm, *w* = 210 nm, *z*_scan_ = 19 nm (**c**). All of the plotted *|*d*C*/d*z*| values are after subtracting the *|*d*C*/d*z*| values found at the centre of the hBN spacer region. Source data

Although the experimental curve resembles the Debye dispersion, the orientational relaxation of water dipoles responsible for the Debye behaviour of bulk water plays little role at *f* < 10 GHz and the dispersion found at our relatively low *f* is caused by conductive losses (Methods). Indeed, at high *f*, the dielectric response is determined by the *ε*_//_ of water and is independent of *σ*_//_, which yields the high*-f* plateau with its strength increasing with increasing *ε*_//_ and *h*. The *σ*_//_ of water starts contributing at frequencies below the conductivity relaxation frequency *f*_r_ = *σ*_//_/2π*ε*_0_*ε*_//_, leading to a stronger dielectric contrast. The conductivity contribution is cut off at *f*_c_ = *ασ*_//_/2π*ε*_0_ by capacitive coupling between the water and the bottom graphite as well as the AFM tip, which results in the low-*f* plateau. The cut-off frequency *f*_c_ is independent of *ε*_//_ and increases proportionally to *σ*_//_ through the geometric factor *α* that depends on *h* and the measurement geometry. The inset of Fig. 1a summarizes the tendencies expected for the dielectric response with changing *h*, *ε*_//_ and *σ*_//_. Further details about SDM measurements are provided in Methods (Extended Data Figs. 4–10).

## Dielectric dispersion of nanoconfined water

As well as the reference spectrum from the 30-nm channels, Fig. 2a shows the spectra obtained for ultrathin water in channels with other representative heights *h* ≈ 5 and 1.5 nm (cyan and red, respectively) and Fig. 2b provides examples of their SDM imaging (see also Extended Data Fig. 4). We can see two clear trends in how the dielectric response of water evolves with decreasing *h*. First, the transition between low-*f* and high-*f* plateaus shifts to higher frequencies, from kHz to MHz. This is also apparent from the images in Fig. 2b, in which changes in the contrast occur at higher *f* for the thinner channel. The behaviour unambiguously shows that water becomes more conductive under strong confinement. This is expected because water is known to interact with hBN walls, which results in near-surface dissociation of water molecules and higher proton conductivity^17,31^. The strongly enhanced *σ* is also consistent with previous results for water inside hBN nanochannels and nanoporous media^32,33^. Second, the high-*f* dielectric response does not gradually diminish with decreasing *h* as expected if *ε* of nanoconfined water were to remain the same as that of bulk water (compare with the inset of Fig. 1a). Instead, the high-*f* plateaus for *h* ≈ 1.5 and 5 nm in Fig. 2 remain of similar height. This observation qualitatively implies that the *ε* of water notably increases under strong confinement. This conclusion follows from the observed strength of the high-*f* signal and does not rely on any modelling. The positive contrast at high *f* for *h* ≈ 1.5 nm is particularly notable if compared with the behaviour found by SDM without a parallel component of *E*. In this case^22^, the dielectric contrast reversed from positive to negative at *h* ≈ 3 nm because of diminishing *ε*_⊥_. This does not happen for the in-plane measurement geometry even for our smallest *h* ≈ 1 nm and again implies large *ε*_//_ for water inside atomic-scale channels.

The measured spectra are essentially determined by the two characteristic frequencies *f*_r_ and *f*_c_ that in turn reflect *ε*_//_ and *σ*_//_ of nanoconfined water, respectively. Accordingly, changes in the frequencies with varying *h* can be translated into relative changes of the corresponding electrical characteristics. With reference to the inset of Fig. 1a, the experimental spectra in Fig. 2a allow us to conclude immediately that both *ε*_//_ and *σ*_//_ increase with decreasing water thickness. However, to find the scale of these changes and the absolute values of *ε*_//_ and *σ*_//_ requires knowledge of the factor *α* that depends on *h* and other geometric parameters. To this end, we used full-3D numerical simulations and calculated the detailed electric-field distribution across our nanochannels. From this analysis, we obtained anticipated dielectric dispersions for different *h* (Extended Data Figs. 5 and 6). Then we determined *σ*_//_ and *ε*_//_ by fitting *f*_c_ and the amplitude of the high-*f* plateau (all other parameters were found experimentally; Extended Data Fig. 10). These simulations also took into account that the dielectric response of nanoconfined water is anisotropic, using the *ε*_⊥_(*h*) dependence reported previously^22^. Examples of this analysis are presented in Fig. 2a by the solid curves. For comparison, the dashed curves in the same figure represent the behaviour expected if the confined water retained the electrical properties of bulk water. We also analysed the experimental spectra using two analytical approximations in which: (1) the AFM tip was simulated as a point charge and the nanochannel as a laterally infinite multilayer stack (Extended Data Fig. 8) and (2) the measurement geometry was simulated using an equivalent electrical circuit (Extended Data Fig. 9). This provided an approximation for *α* as a function of the main parameters, *α* ≅ (*hw*/l*)(1/(2π*R*) + *H*/(*ε*_hBN_wl*)), in which *R* is the AFM tip radius and l* is the effective channel length (Methods). Although less accurate, these two models were found to yield close values for both *ε*_//_ and *σ*_//_. Furthermore, we verified that the extracted values depended little on *ε*_⊥_ (because *ε*_//_ is much larger; see Methods and Extended Data Figs. 6f and 7b,c,e) and were independent of *σ*_⊥_ (the measurement geometry used is insensitive to the latter; see Extended Data Fig. 6e). The use of several approaches have shown that our results are robust with respect to modelling and, also, corroborated our qualitative conclusions drawn from the spectral shifts, as discussed above.

**Fig. 3:**
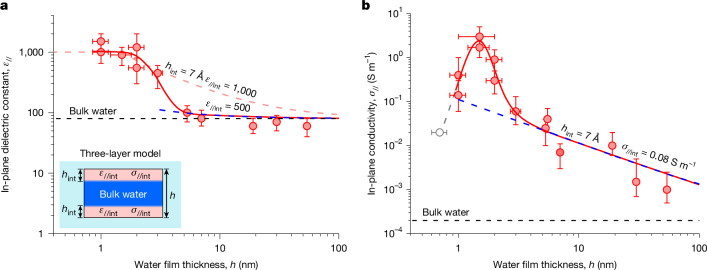
In-plane electrical properties of water under molecular-scale confinement. **a**, Experimental data for *ε*_//_. Red symbols, the mean values of the high-*f* plateaus in the spectra; bars indicate the standard deviations. Solid curve, guide to the eye. Dashed curves, three-layer capacitance model explained in the inset. Shown in light red and blue are the fits of the model with *ε*_//int_ = 1,000 and 150, respectively, using the interfacial-layer thickness *h*_int_ found in ref. ^22^. Black dashed line, *ε*_bulk_. **b**, Red symbols and red curve, same as in **a** but for *σ*. Bars are standard errors from the fits to the cut-off frequency. Grey symbol, experimental value incorporated from ref. ^32^. Black dashed line, bulk conductivity of the water used. Blue dashed line, best fit for *h* ≥ 2 nm using the three-layer model in **a** and assuming different conductivities for bulk and near-surface water. Source data

We have studied many nanochannel devices with *h* ranging from about 1 to 60 nm and, using *f* from several hundred Hz to GHz, obtained their dielectric spectra similar to those shown in Fig. 2a. Then applying the full numerical analysis for each device, the *ε*_//_ and *σ*_//_ of water were extracted as a function of *h*. The results are summarized in Fig. 3.

## In-plane dielectric constant

Inside our channels with *h* > 4 nm, water was found to exhibit *ε*_//_ ≈ 74 ± 17, that is, little difference with respect to bulk water (Fig. 3a). The dielectric constant suddenly changed for quasi-2D water (*h* ≈ 1–2 nm), which showed a sharp increase in *ε*_//_ by an order of magnitude, reaching 1,030 ± 350. Such large dielectric constants are typical of ferroelectrics. This behaviour is in strong contrast to that of *ε*_⊥_ for the same range of thicknesses^22^. The latter constant exhibits a decrease by a factor of about 40, such that quasi-2D water becomes essentially non-polarizable in the perpendicular direction^22^. Although the large *ε*_//_ for water under atomic-scale confinement may seem surprising, the result agrees (at least qualitatively) with most simulations that have predicted enhanced *ε*_//_ and strong dielectric anisotropy for water in the vicinity of solid interfaces^23,24,27–30^. The phenomenon can be understood by noting that, whereas the interaction with solid surfaces hinders the ability of water dipoles to align along *E* applied in the perpendicular direction, the dipoles are relatively free to rotate and align in the in-plane direction.

For large channels, it is reasonable to consider confined water as consisting of an inner layer of bulk water molecules unaffected by the confinement and two near-surface layers with a different interfacial dielectric constant *ε*_//int_, as sketched in the inset of Fig. 3a. This simple model (three capacitors in series) has previously proved successful in describing the observed *ε*_⊥_(*h*) dependence qualitatively well and suggested the presence of an ‘electrically dead’ near-surface layer^22^. Its thickness *h*_int_ was estimated as roughly 7 Å, in agreement with the thickness of layered water at solid interfaces^34^. However, if the same model is applied to explain the in-plane response (three capacitors in parallel, yielding the effective *ε*_//_(*h*) = *ε*_bulk_ + 2(*ε*_//int_ − *ε*_bulk_)*h*_int_/*h*), the observed *ε*_//_(*h*) dependence cannot be described even qualitatively (light red dashed curve in Fig. 3a). The sharp increase at *h* ≈ 2–3 nm clearly indicates a transition in the dielectric properties of water if the two confining surfaces become sufficiently close such that the near-surface layers merge and water develops a more distinctly layered structure^4,35^. The large increase in *ε*_//_ in this case is consistent with theory^28,29^, although the transition was predicted for *h* ⪝ 1 nm. Let us also mention the slight tendency seen in Fig. 3a at *h* > 4 nm for *ε*_//_(*h*) to increase with decreasing *h*. This increase would be consistent with many predictions^27–30^ and, within the three-layer model and our experimental accuracy, suggests values of *ε*_//int_ up to approximately 150 for interfacial water. Measurements at higher accuracy are required to corroborate the latter observation.

## In-plane conductivity

The extracted conductivities for nanoconfined water are plotted in Fig. 3b. We can see that, for moderate confinement, *σ*_//_(*h*) evolves approximately as 1/*h*, increasing from 10^−3^–10^−4^ S m^−1^ for our largest channels up to about 0.1 S m^−1^ at *h* ≈ 3 nm. This gradual increase is well described by the discussed three-layer model (inset of Fig. 3a) assuming unaffected conductivity for the inner layer and the presence of two near-surface layers with interfacial conductivity *σ*_//int_, which yields *σ*_//_(*h*) = *σ*_bulk_ + 2(*σ*_//int_ − *σ*_bulk_)*h*_int_/*h*. Using the same *h*_int_ for interfacial water as for the dielectric constant, we estimate *σ*_//int_ to be approximately 0.1 S m^−1^, three orders of magnitude higher than *σ*_bulk_ (*σ*_bulk_ was measured independently using our bulk water with pH ≈ 5.7 caused by CO_2_ absorption). Such an enhancement in the conductivity of confined water agrees well with previous reports for water inside nanochannels and is attributed to the surface charge^15,16,19,32,33^.

For quasi-2D water, in which the water becomes layered across its entire thickness (*h* ⪝ 2 nm), we observe a pronounced extra peak in conductivity. The behaviour clearly diverges from the surface charge (three-layer) model but correlates well with the anomalous increase in *ε*_//_ (compare with Fig. 3a,b). At its peak, *σ*_//_ reaches about 3 S m^−1^, which occurs at *h* ≈ 1.5 nm and corresponds to 4–5 molecular layers^35^. This value is one of the highest conductivities reported at RT, comparable with that of Nafion and approaching the superionic levels reached for molten salts (about 10 S m^−1^)^36^. Note that our smallest channels (*h* ≈ 1.0 nm) exhibited a marked (more than ten times) decrease in *σ*_//_ with respect to the peak value. To corroborate the presence of the peak for quasi-2D water, Fig. 3b incorporates a data point available in the literature (grey symbol), which was obtained for water inside 0.7-nm hBN channels using DC measurements^32^. This also shows consistency between different types of experiment.

## Discussion

Our experiment shows that water under confinement exhibits two distinct regimes. Under moderate confinement (*h* down to about 2–3 nm), water can be described as effectively containing inner bulk water and interfacial water. The latter is characterized by enhanced conductivity (by at least three orders of magnitude for the case of hBN surfaces) and a highly anisotropic dielectric constant with *ε*_⊥_ ≈ 2 and *ε*_//_ comparable with *ε*_bulk_ or slightly larger. For the extreme, atomic-scale confinement that allows only several molecular layers inside, the quasi-2D water clearly switches to another state. It is different from either bulk or interfacial water and exhibits ferroelectric-like polarizability and superionic-like conductivity (presumably provided by proton transport).

The electrical behaviour observed under moderate confinement (≥ 3 nm) generally agrees with the previous reports and theoretical expectations^15,19,27–30^. As for the atomic-scale confinement, both measurements^4^ and molecular dynamics simulations^35^ have previously shown that water has a distinctive layered structure exhibiting more profound density oscillations than those found for near-surface water. The observed anomalies in the electrical properties of few-layer water align with predictions of giant, ferroelectric-like values of *ε*_//_ (refs. ^28,29^) and superionic conductivity for monolayer water at high temperatures^37^. Experiments on water flow and ionic transport also reported the emergence of strong anomalies in the same few-layer regime^32,38^.

In the absence of a theory, we note that atomic-scale confinement induces strong hydrogen-bond disorder because of constraints on the possible orientations of water molecules and also a loss in hydrogen bonding imposed by the confining surfaces. Qualitatively, this suggests changes in the electrical properties analogous to those found for disordered hydrogen-bonded crystals^39^ and in particular in disordered ice. The latter is known to exhibit higher *ε* than liquid water (and ordered ice) owing to easier reorientation of water molecules^14^. Similarly, we speculate that the disruption of the hydrogen-bond network in quasi-2D water enables correlated reorientations of molecular dipoles, allowing their collective polarization^40^ and consequently higher, ferroelectric-like *ε*_//_ (refs. ^12,28^). Because the reorientation of water dipoles is essential in the Grotthuss mechanism, such changes in quasi-2D water should also facilitate faster proton transport, resulting in higher *σ*_//_, as well as the increased near-surface proton concentration caused by surface charges.

As water becomes structured near all solid interfaces, we expect the qualitative behaviour observed here to be general across various systems and not limited to hBN channels. However, because the molecular arrangements and dipole orientation of water are highly sensitive to surface charge density and polarity, quantitative values of *ε*_//_ and *σ*_//_ probably depend on surface chemistry. For example, recent predictions^27^ suggest that the *ε*_//_ of water can range from bulk-like to giant values near surfaces depending on their polarity. We also note our experimental uncertainty of approximately 30%, arising from the technical challenges inherent to these measurements. This limitation, however, does not affect our central finding: both the in-plane dielectric constant and the conductivity of interfacial water are large and become enhanced under strong confinement, in strong contrast to the suppression observed in the perpendicular direction^22^.

## Conclusion

We have measured the in-plane electrical properties of 2D nanoconfined water and found that they substantially change as the layered structure of water becomes more pronounced under extreme, molecular-scale confinement. Our results provide unique experimental insights into previously inaccessible properties of interfacial and strongly confined water and are fundamental to interpreting numerous physical and biochemical processes occurring at the molecular scale. In particular, they advance our understanding of the electric double layer under strong confinement, which is essential for developing energy storage and generation technologies. The reported approach to measure conductivities and dielectric constants can be refined further and also applied to substances other than water, while having the notable advantage that it examines local properties without averaging over a distribution of different confinement strengths, as inevitable in macroscale measurements.

## Methods

### Device fabrication

We fabricated our devices using procedures similar to those described in ref. ^22^. A free-standing silicon nitride (SiN) membrane was used as a substrate for our van der Waals assembly that involved four atomically flat crystals (Extended Data Fig. 1). We started with etching a rectangular aperture of approximately 3 × 20 μm^2^ in the SiN membrane. This aperture was required to later serve as a water inlet from a reservoir placed at the back of the wafer. Using the dry transfer method, we transferred a graphite crystal (thickness approximately 10 nm) to seal the aperture. The graphite later served as the bottom electrode. It was etched through from the back side using reactive ion etching, which projected the aperture into graphite. Then a relatively thick hBN crystal (*H* ≈ 50–200 nm, to serve as the bottom layer) was transferred on top of the graphite and again dry-etched through from the back. Next we selected a second hBN crystal (thickness *h* ≈ 1–60 nm), referred to as spacer, and patterned it into parallel stripes (spaced by about 200 nm) using electron-beam lithography and reactive ion etching. We transferred the spacer layer on top of the bottom hBN using wet transfer procedures and aligning the stripes perpendicular to the rectangular aperture. Finally, the third hBN crystal was transferred on top of the spacer layer using the dry transfer method. This sealed the nanochannels as well as the aperture. The thickness of the top hBN (*H*_top_ ≈ 20–80 nm) was carefully chosen to bring the AFM tip as close as possible to the water but also to ensure that the top layer exhibits some sagging into empty channels without blocking them completely^22^ (Extended Data Fig. 2a,d). After each transfer, we annealed the assembly in Ar/H_2_ at 300 °C for 3 h and then at 400 °C for 5 h to remove polymer residues and other contamination. As the final step, we made an electrical contact with the graphite using photolithography or, alternatively, using silver paint to minimize the number of cleanroom processes. Because the top layer became flattened (no longer sagged)^22^ after liquid water filled the channels (see Extended Data Fig. 2d–g), this allowed us to ensure that, irrespective of the dielectric response of water, the investigated nanochannels were fully filled with water (by comparing AFM topography images before and after filling; Extended Data Fig. 2e,f). Note that, given the large in-plane conductivity of water found in this work for all thicknesses of channel, we could also verify the water filling by taking atomic force microscopy (AFM) images in the intermittent-contact attractive mode using low-*f* applied voltages (several kHz down to DC). In this case, the acquired contrast over the channels reversed from negative to positive on filling the water (Extended Data Fig. 2b,c) owing to the onset of strong electrostatic forces associated with the conductivity of water, as the AFM feedback adjusted the *z*-scanner to compensate for these forces during the scan. We emphasize that the contrast appearing in Extended Data Fig. 2c (showing roughness of several nanometres) reflects differences in the electrical properties of water and hBN (hence the red/blue colour scale) and not the actual topography, which is shown separately in grey in Extended Data Fig. 2a. When water filled the channels, the sagging disappeared and the surface became atomically flat with residual roughness of less than 3 Å independently of the channel thickness, as described previously^22^. This flattening is demonstrated here using true topography images in Extended Data Fig. 2e,f and their profiles (Extended Data Fig. 2g).

As another improvement with respect to our previous studies, before filling with water, we normally exposed the bottom side of the devices to low-power Ar/O_2_ plasma (8 W, 16 sccm Ar and 8 sccm O_2_ flow) for 1 s. This made SiN more hydrophilic and also cleared the entrances of channels from possible contamination. This procedure has proved beneficial for getting water inside. We also found that our devices tended to delaminate if contamination remained trapped between van der Waals layers. To prevent this from happening, great care was taken to check for cleanliness of the devices at each fabrication step (using optical and AFM imaging). For example, if dark-field optical images showed bubbles trapped between layers, such devices were discarded. Representative optical images of the studied devices with clean interfaces are provided in Extended Data Fig. 1a–c.

### Local broadband dielectric imaging and spectroscopy

All SDM^10,41^ measurements were carried out using a commercial atomic force microscope (Nanotec Electronica with WSxM software^42^) operated at RT in dry atmosphere. SDM was implemented in the amplitude-modulated electrostatic-force detection mode^43,44^, adapting the approach described in ref. ^10^. Briefly, we applied an AC voltage between the AFM tip and the bottom electrode. By detecting mechanical oscillations of the cantilever, we measured the electrostatic force and, therefore, the first derivative of the tip–sample capacitance, d*C*/d*z*. Its value depends on both dielectric and conductive properties of investigated samples. SDM has previously been used for local measurements of both surface and sub-surface dielectric properties of various materials in different frequency regimes, from quasi-static to GHz (refs. ^45–48^). The same approach has also been widely used to study near-surface electron transport in solid samples^49–52^, showing the sensitivity of the technique to local conductivity. The force-sensing approach was preferable in our case to the other current-sensing and microwave-sensing AFM techniques that can also examine local impedance^53,54^ because the latter are generally less sensitive and more complex to implement.

To carry out dielectric spectroscopy over the required very wide bandwidth (100 Hz to 1 GHz), we combined two previously reported force-sensing detection methods. Both were implemented here using conductive diamond-coated AFM probes (CDT-CONTR or CDT-FMR from Nanosensors) with spring constant *k* of several Nm^−1^ and resonance frequency in the range 20–60 kHz. At low *f* up to the cantilever resonance frequency, we used the 2*ω*-detection approach, as described in ref. ^22^ for measurements of the *ε*_⊥_ of water, in which *ω* = 2π*f* is the angular frequency. Briefly, we applied an AC voltage with amplitude *v*_AC_ = 4 V and measured the amplitude *D*_2*ω*_(*z*) of the resulting mechanical oscillations of the cantilever at double the applied frequency, using an external lock-in amplifier (Zurich Instruments HF2LI). The AFM tip–sample capacitance gradient was calculated as *|*d*C*/d*z*| = *D*_2*ω*_(*z*)4*k*/*v*_AC_^2^. Despite the relatively large AC excitation, the response remained within the linear regime, as verified by systematic amplitude-dependence measurements on reference samples and validated using relatively thick channels that exhibited the expected bulk water properties. For *f* higher than the cantilever resonance frequency, we used the heterodyne detection technique demonstrated in ref. ^55^. To this end, we applied a high-*f* (carrier) signal modulated by low frequency (*f*_mod_ = 1 kHz, amplitude *v*_AC_ = 0.5 V) using an external RF/GHz signal generator (Rohde & Schwarz SMA100B). We detected the cantilever mechanical oscillations at *f*_mod_ using our external lock-in amplifier and, from those measurements, obtained the capacitance gradient at the carrier *f* as *|*d*C*/d*z*| = *D*_mod_(*z*)8*k*/(*gv*_AC_^2^), in which *g* is the *f*-dependent gain of the external circuit and *D*_mod_(*z*) is the amplitude of the cantilever mechanical oscillations at *f*_mod_. This approach allowed retrieval of *|*d*C*/d*z|* variations at *f* higher than the cantilever resonance frequency and up to GHz frequencies^51^. Note that such measurements yield only the amplitude of d*C*/d*z* and not its phase, so the obtained spectra reflect the modulus of the complex dielectric constant (see the ‘Analysis of local dielectric spectra’ section).

To minimize systematic errors, we carefully calibrated the electrical gain *g* for each device and atomic force microscopy tip at each measurement frequency. This is essential at high frequencies (> 1 MHz), at which both applied and measured signals show strong *f*-dependent variations because the cable impedance is not matched to the local sample impedance and the long-range impedance between the AFM tip and the sample. We used a calibration procedure similar to that in ref. ^47^, which relies on acquiring *|*d*C*/d*z|* curves as a function of the tip–surface distance *z* over a device region with *f*-independent impedance. We determined *g* by comparing *|*d*C*/d*z*|(*z*) curves at each frequency against a low-*f* reference curve (2 kHz), for which no gain or loss is expected. To avoid potential changes in *g* in different regions of the device, these curves were taken over the hBN spacer near the water-filled channel (Extended Data Fig. 3a). We then scaled them to match the 2-kHz curve using *g* and the offset as the two fitting parameters (Extended Data Fig. 3c). Note that, irrespective of *f*, the offset is always present in d*C*/d*z* curves and is typically subtracted before analysis, because it is independent of local electrical properties (see, for example, refs. ^10,56^). We found that, at high *f* (10 MHz to 1 GHz), the offsets changed substantially but were identical above water channels and hBN spacers. This confirmed that, for all of the frequencies, the offsets originated from long-range impedance contributions. Notably, although *g* is critical for accurately evaluating the electrical properties of water from the measured signals, offsets play little role in this study because we analysed the relative changes in *|*d*C*/d*z|* along the device surface–water channels versus hBN spacers (see the ‘Dielectric images and dielectric spectra acquisition’ and ‘Numerical modelling and data analysis’ sections). We validated the calibration using reference samples (hBN on doped Si), obtaining the expected *f*-independent dielectric spectra up to GHz frequencies (Extended Data Fig. 3e,f). Furthermore, large channels filled with water effectively served as another reference, yielding the expected spectral behaviour (flat response at high *f*) and the dielectric constant of bulk water. To prevent electrostatic crosstalk in calibration curves taken above hBN spacers from water inside the channels, we used channels separated by about 800 nm, a spacing chosen to be sufficiently large. Numerical simulations confirmed that, within our experimental accuracy (roughly 1 zF nm^−1^), the calibration curves were unaffected by electrostatics from water in the channels (Extended Data Figs. 5b and 7f). We emphasize that, if the separation were too small, such crosstalk could lead to underestimation of the dielectric constant of water.

### Dielectric images and dielectric spectra acquisition

The dielectric images in Fig. 1 and Extended Data Fig. 4 were taken at constant height *z*_scan_ (typically about 15–25 nm) from the top layer, as in ref. ^22^, using the constant-height dual-pass mode SDM developed earlier^10,41^. In this mode, the first pass records the topography without applied voltage and the second pass acquires the dielectric image with the AFM feedback loop disabled and the *z*-scanner fixed (the scanner was carefully aligned parallel to the sample plane so that *z*_scan_ remained constant along the horizontal direction). To avoid piezo creep effects, we allowed sufficient settling time at the beginning of each line for the scanner to reach the target height. This approach ensures maximum control of *z*_scan_, as this is measured with sub-nanometre accuracy by acquiring DC and AC deflection approach curves at scan line edges (here over the hBN spacer regions)^10^. The DC deflection curve allows measuring the tip–surface distance *z* with sub-nanometre accuracy, whereas the AC deflection curve provides *|*d*C*/d*z|* as a function of *z*, allowing further validation of *z*_scan_ and correction of *z*-scanner drifts by matching with the *|*d*C*/d*z|* scan line (Extended Data Fig. 3b,c). Because the second-pass scan maintains the *z*-scanner fixed, potential artefacts from the *z*-scanner motion that would arise in the standard dual-pass ‘lift’ mode are avoided. Small variations in *z*_scan_ owing to the DC electrostatic force are also measured and taken into account by recording the DC deflection during the second pass. The images as a function of *f* reported in Fig. 2b were taken at both constant *z*_scan_ and constant *y* position (*y*-axis is along the nanochannels length). We typically recorded 25 lines at each *f*. Standard AFM image processing consisting of flattening and Gaussian filtering was applied.

The dielectric spectra in Fig. 2a and Extended Data Fig. 10 were obtained for constant *z*_scan_ (about 15–20 nm) and constant *y* but after acquiring the entire 3D dataset *|*d*C*/d*z*|(*x*,*z*,*f*), such as that shown in Extended Data Fig. 3d. This set is composed of many *|*d*C*/d*z*|(*x*,*z*) images (Extended Data Fig. 3a), which were obtained at different *f* by scanning the AFM tip across the channels (along the *x*-axis) at constant *y* and approaching the surface in steps. This refined procedure was necessary here to minimize errors in *z*_scan_ owing to *z*-scanner drifts during long measurements across the whole frequency sweep. Although the spectral behaviour does not change with scan height, the amplitude of *|*d*C*/d*z|* does (Extended Data Fig. 6g,h), making it essential to maintain the same *z*_scan_ at each *f* for high accuracy in our spectral analysis. When taking *|*d*C*/d*z*|(*x*,*z*) images, the AFM tip was approached from larger distances towards the surface down to the minimum distance of typically about 15 nm, beyond which tip collapse occurred owing to long-range attractive electrostatic forces, as expected under our measurement conditions (applied voltage, RT, soft cantilevers and large AFM tip radii). At each step, we acquired dozens of lines, which were then averaged to obtain the corresponding profile (Extended Data Fig. 3b). This notably improved the signal-to-noise ratio. By taking small steps towards the surface, we could then reconstruct the dielectric spectra (Extended Data Fig. 10) by using the value measured in the middle of nanochannels at the same *z*_scan_ (±1 nm) for each *f*. This 1-nm uncertainty has negligible impact on the extracted electrical properties shown in Fig. 3, as demonstrated in Extended Data Fig. 7g,h (see also the ‘Numerical modelling and data analysis’ section). We note that all of the spectra were obtained at the nearest possible distances achievable before tip collapse to ensure maximum signal strength and measurement stability. Larger *z*_scan_ were avoided, as the *|*d*C*/d*z|* signal decays rapidly with *z*, compromising measurement accuracy, particularly for small channels (Extended Data Fig. 6g,h).

As well as the scanning height *z*_scan_, the spectral values depended on geometry/size of our devices and the AFM tip parameters (Extended Data Fig. 10). All of the necessary geometric parameters of the studied devices were measured by taking their topography images, whereas the AFM tip parameters (its radius *R* and half-angle *θ*) were determined by fitting *|*d*C*/d*z*|(*z*) curves taken directly above the graphite bottom layer, as in ref. ^22^, following the procedures described in refs. ^56,57^. For example, the tip radii were found to be in the range 50–200 nm, in good agreement with the values specified for commercial diamond-coated tips. This enabled quantitative analysis of the ‘absolute-value’ spectra (shown in Extended Data Fig. 10) as opposed to just relative variations with *f*, through full-3D numerical simulations that account for the detailed geometry of the system (see the ‘Numerical modelling and data analysis’ section). We emphasize that the values of *|*d*C*/d*z|* in the spectra that we analysed to extract the electrical parameters of water are the peak values over the centre of the water channel relative to the values measured over the centre of the hBN spacers at the same scan height, as in ref. ^22^. This differential approach makes our analysis robust against uncertainties in geometric parameters and eliminates the influence of the long-range geometry of the system, as described below.

To facilitate direct comparison of the electrical response of water across different devices and experiments and enhance clarity of presentation, we normalized our representative spectra for various water thicknesses in Fig. 2a. This normalization used the low-*f* plateau as a reference point, dividing each spectrum by this value. In this low-*f* regime, the measured signal becomes effectively independent of both the electrical properties of water and its thickness (see Extended Data Figs. 6b and 8b and the ‘Analysis of local dielectric spectra’ section). As the signal in this regime is primarily determined by the AFM tip radius and its distance from the channel, normalizing with respect to the low-*f* value effectively eliminates the geometric contributions, allowing for more direct comparison between different experiments. We emphasize that alternative normalization using high-*f* values would not be beneficial, as the signal in this regime strongly depends on both the channel thickness and its dielectric properties.

### Numerical modelling and data analysis

The observed dielectric spectra were fitted to full-3D finite-element numerical calculations implemented using COMSOL Multiphysics 5.4a (AC/DC electrostatic module), not other simplified models presented in the manuscript. These 3D calculations were based on the electrostatic model previously used in ref. ^22^, adapted here to simulate the frequency-dependent force acting on the tip as a function of *ε* and *σ* of water when an AC electric field was applied. They compute absolute |d*C*/d*z|* values while fully accounting for the actual geometry and dimensions of the system, including the tip and device, as well as the conductive and dielectric properties of water and their anisotropy, thereby eliminating potential geometric and electrostatic artefacts that could arise in the data analysis using simplified models.

A schematic of the model is shown in Extended Data Fig. 5a. Following ref. ^22^, the AFM tip was modelled as a truncated cone with the half-angle *θ* terminated with a tangent hemispherical apex of radius *R*. The values of *θ* and *R* used in our simulations were measured for each AFM tip, as discussed above. The AFM cone height *H*_cone_ was limited to 6 μm and the cantilever was omitted to reduce the computational time (unless stated otherwise). We checked that these approximations had no impact on the simulated results. The simulated nanochannel consisted of two insulating slabs of hBN separated by a lossy water slab of height *h* and width *w* (measured from topography for each device). We modelled each slab explicitly with its own respective dielectric constant and conductivity according to the general definition of anisotropic, complex dielectric constant

1 $${\varepsilon }_{\perp ,//}^{* }(\omega )={\varepsilon }_{0}{\varepsilon }_{\perp ,//}-{\rm{i}}\frac{{\sigma }_{\perp ,//}}{\omega },$$

in which *ω* is the angular frequency, *ε*_0_ is the dielectric permittivity of vacuum, *ε*_⊥_ and *ε*_//_ are the dielectric constants perpendicular and parallel to the channel, respectively, and *σ*_⊥_ and *σ*_//_ are the corresponding conductivities, respectively. Note that, for the water slab, the imaginary term in equation (1), represented by *σ*_⊥,//_, accounts for all possible losses, including those from charge transport and dipolar relaxation, whereas *ε*_⊥,//_ may contain ionic contributions owing to ion–water and ion–ion correlations, as well as the purely dielectric response of water^58,59^. Both *ε*_⊥,//_ and *σ*_⊥,//_ of water were treated here as frequency-independent, as this approximation adequately reproduces the observed dispersion, as explained below and also in the ‘Analysis of local dielectric spectra’ section. For hBN, *σ*_⊥,//_ is zero, so its ${\varepsilon }_{\perp ,//}^{* }$ reduces to its known real part, *ε*_⊥hBN_ = 3.5 and *ε*_//hBN_ = 5.5, which are constant within our experimental bandwidth. To model possible contributions from nearby nanochannels, the device was modelled as three parallel water nanochannels of length *l* = 3 μm and measured spacing *w*_s_ within the surrounding hBN dielectric matrix, with dimensions matching the measured top, bottom and spacer hBN layers (length *l* = 3 μm, width *W* = 3 μm and height *H*_top_ + *h* + *H*).

We numerically solved the Poisson’s equation in the frequency domain for each device, calculated the force acting on the AFM probe and, from that, obtained *|*d*C*/d*z|* by integrating the built-in Maxwell stress tensor on the surface of the probe. The simulations used the same box size and boundary conditions as in ref. ^22^. Examples of calculated dielectric spectra for representative geometrical parameters of our devices and *ε* and *σ* of water are shown in Extended Data Fig. 6. These simulations closely match the experimental spectra, reproducing the observed Debye-type frequency dispersion.

Our modelling approach is equivalent to the original analysis by Maxwell and Wagner^60,61^, which led to the Debye-like Maxwell–Wagner (MW) formalism commonly used to interpret dielectric relaxations arising from DC conductivity in macroscale spectra of heterogeneous lossy dielectrics^62–64^ (see the ‘Alternative phenomenological Debye-like MW analysis’ section). Using the complex dielectric constants defined in equation (1), Maxwell and Wagner showed that the effective capacitance of a planar layered system, in which each layer has constant *ε* and *σ*, acquired a frequency dependence similar to a Debye-type relaxation^62,63^. In our case, the AFM tip replaces the top electrode in their derivation, precluding an exact analytical solution and necessitating 3D numerical simulations. Nevertheless, the underlying electrostatic problem is the same, therefore no further frequency dependence of *ε* and *σ* in equation (1) for the water slab is required to reproduce the observed dispersion, unless an extra relaxation process is present. In our case, no such further relaxation needs to be assumed or is expected to occur, based on present understanding of strongly confined water. Consistent with this, the experimental spectra exhibited only a single Debye-like relaxation at lower frequencies arising from DC conductivity (for more details, see the ‘Analysis of local dielectric spectra’ section).

Experimental *|*d*C*/d*z|* spectra were fitted with the *ε*_//_ and *σ*_//_ of water as the only fitting parameters. All of the other parameters needed for the simulations were determined experimentally, as detailed above. *ε*_⊥_ was set to the values measured in ref. ^22^ and *σ*_⊥_ was set to the measured value for our bulk water (*σ*_bulk_ = 2 × 10^−4^ S m^−1^). The extracted *ε*_//_ and *σ*_//_ values were found to depend little on exact values of *ε*_⊥_ or *σ*_⊥_, indicating that our experimental geometry was rather insensitive to the out-of-plane characteristics of water, and the influence was negligible for our smallest channels (Extended Data Figs. 6e,f and 8e,f). This is because the impedance of the nanochannel in the out-of-plane direction is much smaller than the series impedance from the hBN layers and AFM tip–surface capacitances, whereas the impedance of the nanochannel in the in-plane direction is much larger (see the ‘Electrical modelling’ section). From these fitted values of *ε*_//_ and *σ*_//_, we inferred the interfacial dielectric constant, *ε*_//int_, and conductivity, *σ*_//int_, of the confined water layer using the three-capacitor model, as explained in the main text, without introducing *ε*_//int_ and *σ*_//int_ directly into the electrostatic problem. This approach minimized the complexity of our numerical calculations.

For brevity, in all of the figures, *|*d*C*/d*z|* refers to the relative dielectric contrast (unless stated otherwise), that is, the response relative to the hBN spacer so that *|*d*C*/d*z*| ≡ d*C*(*z*_scan_,*ε*_⊥,//_,*σ*_⊥,//_)/d*z* *−* d*C*(*z*_scan_,*ε*_⊥,//hBN_,0)/d*z*. Accordingly, we computed the absolute value of *|*d*C*/d*z|* over the centre of the water channel as a function of *f* and subtracted the corresponding values for the case of the tip placed over the centre of the hBN spacer, matching the experimental data processing. This differential approach avoids systematic errors. As mentioned above, it reduces the impact of uncertainties in geometric parameters and allows us to model only the local geometry, as the long-range geometric contributions do not affect *|*d*C*/d*z|* variations relative to the spacer. Furthermore, each device was modelled using its actual dimensions (spacer and channel width and height, top and bottom hBN heights) and with the measured radius of the AFM tip used in that experiment. This ensured that the 3D calculations reproduced the realistic electric-field distribution between the tip and the confined water, yielding reliable electrical properties of water with minimal approximations.

The thickness *H* of the hBN bottom layer determines whether the measurements are mostly sensitive to *ε*_//_ or *ε*_⊥_. Extended Data Fig. 7 illustrates this for the case of channel thickness *h* = 5 nm at high *f*, beyond the conductivity relaxation regime (see the ‘Analysis of local dielectric spectra’ section) and for a large AFM tip radius (100 nm), as used in our experiments. When the channel is in the immediate proximity to a metallic surface (Extended Data Fig. 7a), as in ref. ^22^, the *|*d*C*/d*z|* signal is independent of *ε*_//_ and, consequently, the extracted *ε* represents the out-of-plane component, *ε*_⊥_. Furthermore, measurements are sensitive only to relatively small values of *ε*_⊥_ (up to about 20 for *h* = 5 nm), as the signal saturates with further increases in *ε*_⊥_ (for larger channel thicknesses, the sensitivity extends to increasingly larger values of *ε*_⊥_, as shown in ref. ^22^). Conversely, when the channel is on a thick hBN layer (*H* = 200 nm; Extended Data Fig. 7b), the *|*d*C*/d*z|* signal greatly increases for *ε*_//_ > 20, becoming dominated by the in-plane component. Therefore, for relatively large values of *ε*_//_ ranging from about 80 to about 1,000, as measured in this work, the signal at such *H* shows little dependence on *ε*_⊥_. Extended Data Fig. 7c shows how the signal evolves as a function of *H*. In these simulations, we increased the cone height and cantilever length of the AFM tip to their nominal values (*H*_cone_ = 12 μm and *L*_cantilever_ = 20 μm). This allowed us to include electrostatic contributions from longer-range components of the atomic force microscopy probe^65^, which we found to be relatively small but still non-negligible for very thick bottom hBN (*H* > 500 nm). The results show that the *|*d*C*/d*z|* signal and its sensitivity to *ε*_//_ peaks between 50 and 500 nm (Extended Data Fig. 7c). For thinner hBN layers, the *ε*_//_ contribution is relatively small or negligible and the signal is dominated by *ε*_⊥_. For hBN thicker than 500 nm, the *|*d*C*/d*z|* signal decreases, becoming less sensitive to the investigated local electrical properties, consistent with previous results^56^. This is expected because the AFM tip is moved far away from the bottom electrode. In this work, we used bottom hBN layers of thickness up to 200 nm. This limitation was dictated by constraints in the fabrication of our devices, because thicker hBN crystals were stiffer and provided poor adhesion, leading to delamination on filling the channels with water.

We emphasize that, because the amplitude of the dielectric spectra depends on the scan height, data analysis may also be affected by this parameter. Specifically, although the value of *σ*_//_ is independent of *z*_scan_ (as the cut-off frequency remains unchanged with scan height; Extended Data Fig. 6g,h), the extracted *ε*_//_ may be influenced, as it is given by the amplitude of the high-*f* plateau. As described above, in this study, we used special procedures to control *z*_scan_ and determine its value with maximum possible accuracy, estimated at ±1 nm. Despite the steep increase of *|*d*C*/d*z|* with decreasing *z*, such 1-nm experimental uncertainty has negligible impact on the extracted *ε*_//_, even for our smallest channels. This is because our analysis relies on the measurement of variations of *|*d*C*/d*z|* relative to the hBN spacer region at the same *z*_scan_. This differential approach makes the analysis robust against small uncertainties in *z*_scan_. To illustrate this robustness, Extended Data Fig. 7h shows 3D simulated curves for the best-fit *z*_scan_ value (corresponding to the data in Extended Data Fig. 10c) and for best-fit *z*_scan_ ± 1 nm. The three curves nearly overlap, with experimental data points scattered around them, consistent with the stated ±1 nm experimental uncertainty. The obtained 30% error in the extracted *ε*_//_ = 900 incorporates this uncertainty, along with the standard deviation for data points on the high-*f* plateau. Notably, qualitative experimental evidence allows us to discard large uncertainties in *z*_scan_ as an alternative explanation for the enhanced *ε*_//_ observed in our smallest water channels. Indeed, to justify the large values of *|*d*C*/d*z|* observed at high *f* with bulk-like *ε*_//_ would imply extremely small scan heights (⪝5 nm). This is shown in Extended Data Fig. 7g, in which we plot the sensitivity curve of *|*d*C*/d*z|* to *ε*_//_ at various values of *z*_scan_ for 1.5-nm channels. However, such small values of *z*_scan_ are physically impossible in our constant-height scan mode, as below about 15 nm, the tip collapses onto the surface (see the ‘Dielectric images and dielectric spectra acquisition’ section). Also, the implied small values of *z*_scan_ would lead to much larger *|*d*C*/d*z|* values than observed for the low-*f* spectral plateau, which is highly sensitive to the scan height (see the ‘Analysis of local dielectric spectra’ section). In other words, although the high-*f* plateau could theoretically be reproduced by assuming a much smaller scan height (roughly 5 nm for the case of the representative device with *h* ≈ 1.5 nm in Extended Data Fig. 10c), such a simulation would fail to reproduce the low-*f* plateau, as shown in Extended Data Fig. 7h. These observations rule out substantial errors in *z*_scan_ and the large *ε*_//_ reported here for our smallest devices can be explained by inaccuracies in the scan height.

We stress that the electric field above the hBN spacer varies little with the electrical properties of confined water (Extended Data Fig. 5c–h) and that our differential analysis relative to the hBN spacer region does not introduce systematic errors associated to such electric-field variations. This is demonstrated in Extended Data Fig. 5b, which shows full-3D numerical simulations of the absolute value of *|*d*C*/d*z|* versus tip-surface distance *z* calculated with the tip above the centre of the hBN spacer at different *f*, comparing water-filled and empty channels, for our smallest channels (*h* = 1.5 nm). All curves practically overlap, with deviations at intermediate and high *f* not exceeding 0.1 zF nm^−1^, an order of magnitude smaller than our experimental uncertainty (about 1 zF nm^−1^). Only at the lowest *f* do the deviations increase notably because of the in-plane conductivity of water but still remain within experimental uncertainty. Furthermore, despite their minimal impact, these small variations are fully accounted for in our analysis, which incorporates the realistic electric-field distribution and device geometry. This also shows that, even if these field variations were not accounted for—for example, if using the simplified analytical model described below—the errors would be negligible, particularly at GHz frequencies at which we extract *ε*_//_.

### Analytical modelling

Because of the non-uniform distribution of electric field across nanochannels and the complex geometry of AFM probes, the electrostatic problem cannot be solved exactly using analytical models. Hence, to fit the experimental data and obtain the results shown in Fig. 3, we used the 3D numerical simulations described above. Nonetheless, it is informative to provide an analytical approximation that would substantiate our numerical results and offer further physical insight into the observed spectral behaviour that is relatively independent of the details of experimental geometry. Such an approximation could be used for semi-quantitative estimates. To this end, we used the point-charge model in which the AFM tip was replaced by a point charge *Q* positioned at distance *z* *=* *z*_scan_ + *R* from the top hBN surface (Extended Data Fig. 8a). The devices were modelled as a stack of different slabs, infinite in the in-plane direction. The slabs corresponding to the hBN layers were positioned at 0 < *z* < *−H*_top_ and *−*(*H*_top_ + *h*) < *z* < *−*(*H*_top_ + *h* + *H*) and modelled as insulators with zero conductivity. The water layer at *−H*_top_ < *z* < *−*(*H*_top_ + *h*) was described by anisotropic dielectric constants *ε*_//_,_⊥_ and conductivities *σ*_//_,_⊥_. The boundary condition of zero voltage was imposed at *z* = *−*(*H*_top_ + *h* + *H*) to model the highly conducting ground electrode. By making use of the in-plane translational invariance, the Laplace equation for the Fourier transform electrostatic potential in the in-plane direction *ϕ*_*q*_(*z*) becomes

2 $${q}^{2}{\varepsilon }_{//}(z){\phi }_{q}(z)-{\partial }_{z}({\varepsilon }_{\perp }(z){\partial }_{z}{\phi }_{q}(z))={Q}_{q}\delta (z-{z}_{{\rm{scan}}}-R)$$

in which **q** = (*q*_*x*_,*q*_*y*_) is the wave vector in the in-plane direction and the position-dependent dielectric constants are denoted as *ε*_//_(*z*) and *ε*_⊥_(*z*), respectively. The dielectric constant of the hBN slabs was, for simplicity, assumed to be isotropic with *ε*_//_(*z*) *=* *ε*_⊥_(*z*) = *ε*_hBN_ = 4. The Laplace equation was solved within each slab using exponential functions. The solutions were matched at each interface by imposing the following boundary conditions: continuity of *ϕ*_*q*_(*z*) and

3 $${\varepsilon }_{\perp }(z+\eta ){\partial }_{z}{\phi }_{q}(z+\eta )-{\varepsilon }_{\perp }(z-\eta ){\partial }_{z}{\phi }_{q}(z-\eta )=0$$

in which *η* is an infinitesimal constant. A similar relation holds at the point-charge location, *z*_scan_ + *R*, but the right-hand side in equation (3) becomes equal to *Q*. The potential in real space *ϕ*(*r*,*z*) was then obtained by performing the Fourier transform of *ϕ*_*q*_(*z*) in the in-plane direction. The capacitance was estimated as *C* = *Q*/*ϕ*(0,*z*_scan_) and, from the latter, the capacitance gradient *|*d*C*/d*z|* was calculated. Examples of the calculated dielectric spectra for representative parameters of our devices and *ε* and *σ* of water are given in Extended Data Fig. 8, in which, again for brevity, *|*d*C*/d*z|* indicates its value relative to the case of the heterostructure fully made of hBN, *|*d*C*/d*z*| = d*C*(*z*_scan_,*ε*_⊥,//_,*σ*_⊥,//_)/d*z* *−* d*C*(*z*_scan_,*ε*_hBN_,0)/d*z*. The calculated spectra show that the model captures all of the main features of the observed behaviour as a function of various parameters and agrees well with our numerical simulations in Extended Data Fig. 6. Note, however, that, because the analytical model assumes an infinite water layer and does not account for a finite *w*, the transition between low-*f* and high-*f* plateaus becomes less pronounced (Extended Data Fig. 8d). The analytical results agree with the numerical simulations only for *w* much larger than the tip radius *R* (Extended Data Fig. 6d). Accordingly, if only the analytical model were used to fit the reported experimental data, this would result in systematic underestimation of both *ε*_//_ and *σ*_//_. In particular, the values extracted for quasi-2D water layers (*h* ⪝ 2 nm) would be underestimated by a factor of about 5, although all of the trends with changing *h* would remain correct.

### Analysis of local dielectric spectra

The observed dispersion arises from DC conductive losses of water within the channels, not from its dipolar orientational (Debye) relaxation^66^. The latter in bulk water occurs only at *f* ≈ 10 GHz (ref. ^67^), well above the frequencies examined here. Furthermore, the spectra exhibit very high low-*f* plateaus, which, if fitted with Debye-like models, would yield physically implausible values for the *ε*_//_ of water (see the ‘Alternative phenomenological Debye-like MW analysis’ section). Instead, these plateaus are accurately reproduced by our model using realistic values of *σ*_//_ of water in the conductive term of equation (1). A similar situation is encountered in the analysis of macroscale dielectric spectra of heterogeneous lossy dielectrics measured by broadband dielectric spectroscopy^62^ (for example, colloidal suspensions and composite liquid/solid materials such as water confined in porous media^68,69^), in which MW interfacial polarization relaxations^60,61^ emerge. In such complex heterogeneous systems, phenomenological Debye-like models become necessary, because the measured response represents the effective dielectric constant of the entire system and explicit modelling of each dielectric component is precluded by geometric complexity. Although these approximations can fit macroscale spectra, they typically yield unrealistically high *ε* at low frequencies, often reaching very large values (>10^4^). Such high apparent permittivities, also reported for water confined in porous media^68^, do not represent intrinsic dielectric constants but, rather, reflect DC conductive losses^68,70^. By contrast, our three-layer nanochannel system enables a ‘first-principles’ description in which each dielectric layer is modelled explicitly with its own frequency-independent *ε* and *σ*, as in equation (1). This approach allows *ε*_//_ and *σ*_//_ to be determined independently from the spectra without introducing unphysical parameters or approximations.

No further frequency dependence of *ε*_//_ and *σ*_//_ of water was required in this analysis, as the measured spectra show only a single Debye-like relaxation at low *f* arising from DC conductivity, with no indication of more relaxations. This is evident from the high-*f* plateaus, which remain flat and correspond to bulk-like or enhanced values of *ε*_//_. If in our smallest channels (<4 nm) the intrinsic dipolar relaxation of water were shifted to lower *f* by the geometric confinement, we would expect either an extra dielectric plateau or broadening of the Debye-like relaxation transition region, both leading to reduced high-*f* values of *ε*_//_. Instead, we consistently observed high, flat plateaus at high *f* across all of our devices, ruling out further dipolar relaxations in the in-plane direction. The same reasoning applies to anomalous Debye-like dipolar relaxations of the hydrogen-bond network reported for confined water^69^ in the 100 kHz to 10 MHz range, which would also reduce both the low-*f* and the high-*f* plateaus, an effect not observed here. Although a weak dipolar relaxation from hydrogen-bond restructuring in the out-of-plane direction cannot be entirely ruled out in our largest channels, this is unlikely for our smallest channels, which show very low *ε*_⊥_ at low frequencies (kHz), as previously reported^22^. In any case, variations in *ε*_⊥_ have negligible impact on the extracted *ε*_//_ and *σ*_//_ and, therefore, would not alter our conclusions.

The reason for a DC conductivity contribution to our spectra can be understood by noticing that we measured the modulus of d*C*/d*z*, that is, the modulus of the complex dielectric constant ${|\varepsilon }_{\perp ,//}^{* }(\omega )|$. As a result, the dielectric response is determined by both *ε* and *σ* and, depending on frequency, either the first or the second term in equation (1) becomes dominant. At sufficiently low *f*, the conductivity term always dominates ${|\varepsilon }_{\perp ,//}^{* }(\omega )|$, similar to the case of macroscale measurements using standard broadband dielectric spectroscopy^62^. To this end, it is useful to recall that, at low frequencies, the spectrum of deionized water at macroscale is known^67^ to be dominated by *σ*_bulk_. Therefore, the spectrum is effectively divided into two regions separated by the conductivity relaxation frequency, *f*_r,bulk_, at which the behaviour changes. In the conduction-dominated region (*f* < *f*_r,bulk_), the dielectric response decreases with increasing *f*, whereas for *f* > *f*_r,bulk_, it is constant and depends only on *ε*_bulk_. The value of *f*_r,bulk_ is given by^62^

4 $${f}_{{\rm{r,bulk}}}=\frac{{\sigma }_{{\rm{bulk}}}}{2{\rm{\pi }}{\varepsilon }_{0}{\varepsilon }_{{\rm{bulk}}}}$$

which follows from equation (1) if we use |*ε*_⊥,__//_| = *ε*_bulk_ and |*σ*_⊥,//_| = *σ*_bulk_. Note that this frequency is analogous to the cut-off frequency for systems that can be modelled by a simple RC circuit (see the ‘Electrical modelling’ section). For the bulk water used in our experiments (*ε*_bulk_ ≈ 80, *σ*_bulk_ ≈ 2 × 10^−4^ S m^−1^), equation (4) yields approximately 45 kHz. This value agrees well with *f*_r,bulk_ obtained from both numerical and analytical modelling discussed above for the specific experimental geometry. Indeed, the analyses shown in Extended Data Figs. 6b and 8b yielded the purely dielectric behaviour characterized by high-*f* plateaus starting at *f* ⪞ 45 kHz for all of our nanochannels, irrespective of their height *h*. The extra plateau found in our simulations at low *f* reflects the presence of non-lossy dielectrics (air gap between the AFM tip and the device; the top and bottom hBN layers). These dielectrics can be represented as further capacitances in series to the contribution of water (see the ‘Electrical modelling’ section). Accordingly, for *f* below the cut-off frequency *f*_c,bulk_, the modelled response becomes purely dielectric, reflecting the series capacitances. For *f*_c,bulk_ < *f* <*f*_r,bulk_, the response is dominated by the *σ* of water, whereas above the relaxation frequency *f*_r,bulk_, it is again purely dielectric but now dominated by the *ε* of water. For other relevant values of *σ* and *ε* of water, the spectra exhibit similar behaviour (Extended Data Figs. 6e,f and 8e,f): there is a low-*f* plateau up to the cut-off frequency *f*_c_, above which the response decreases with increasing *f*, and the high-*f* plateau develops above the conductivity relaxation frequency *f*_r_.

The onset of the low-*f* plateau with decreasing *f* is determined by the in-plane conductivity of water *σ*_//_, whereas no information can be inferred about *σ*_⊥_ from our experimental data because the measurement geometry is insensitive to the latter conductivity. This can be seen in Extended Data Figs. 6e and 8e, in which the low-*f* plateau shifts in frequency with varying *σ*_//_, but not *σ*_⊥_, and completely disappears if *σ*_//_ = 0 and *σ*_⊥_ ≠ 0. Qualitatively, the stronger dielectric response at low *f* reflects the fact that the electric potential drops mostly between the AFM tip and the conductive water layer. We used our numerical simulations to illustrate this effect (Extended Data Fig. 5). At low *f* and *σ*_//_ ≠ 0, the electric potential decreases almost entirely across the air and the top hBN layer with little voltage drop left below the water layer (Extended Data Fig. 5f). Also, the potential distribution extends along the length of the nanochannel for several micrometres (Extended Data Fig. 5d). By contrast, at high *f* > *f*_r_, the potential drop occurs across the entire thickness of our devices, similar to the case in which the AFM tip is placed above hBN spacers (Extended Data Fig. 5f–h). The potential drop at high *f* also extends nearly equally in all of the lateral directions around the tip apex (Extended Data Fig. 5e), similar to the case in Extended Data Fig. 5c for non-conducting water layer (*σ*_//_ = 0). The effective screening by the conductivity of water along the channel length explains the observed large low-*f* response. Note that the absolute value of the low-*f* response is independent of *σ*_//_ but depends on geometric parameters, in particular the channel width *w*, tip radius *R* (Extended Data Fig. 6c) and the bottom-layer thickness *H* (Extended Data Figs. 7c and 8c). This makes simulations essential for accurate evaluation of the magnitude of changes on the dispersion curves.

Although the value of *σ*_//_ does not affect the low-*f* response, it controls the cut-off frequency *f*_c_, which shifts to higher *f* proportionally to *σ*_//_ (Extended Data Figs. 6e and 8e) but independently of *ε*_//_ (Extended Data Figs. 6f and 8f). This behaviour can be described by

5 $${f}_{{\rm{c}}}=\alpha \frac{{\sigma }_{//}}{2{\rm{\pi }}{\varepsilon }_{0}},$$

in which *α* is the geometrical parameter that can be estimated analytically (see the ‘Electrical modelling’ section). For more accurate results, *α* was obtained using numerical simulations (Extended Data Fig. 10), which yielded *α* ≈ 2.8 × 10^−3^, 6.2 × 10^−4^ and 9.2 × 10^−5^ for our three representative devices discussed in the main text, with *h* ≈ 30, 5 and 1.5 nm, respectively. These values of *α* suggest that *f*_c_ should shift relatively little (from about 10 kHz to about 500 Hz) if water filling the channels exhibited the bulk properties (Extended Data Figs. 6b,c and 8b,c). This shift is much smaller than that observed experimentally and, in fact, occurs in the opposite direction, towards lower *f* for smaller *h*, in strong contrast to the experimental behaviour (Fig. 2a). This reiterates the fact that the observed increase in *f*_c_ with decreasing *h* cannot be associated with changes in geometry but comes from a huge increase in the *σ*_//_ of water for stronger confinement.

At high *f*, beyond the conductivity relaxation regime, water behaves as a purely dielectric media and, accordingly, the relative height of the high-*f* plateau is no longer dependent of *σ*_//_ but depends on the *ε* of water, the geometry of the channel (in particular its height *h*) and the AFM tip radius (Extended Data Fig. 6c). The height of the plateau allows us to extract *ε*_//_ using numerical simulations. Notably, for small channels and large in-plane dielectric constant (*ε*_//_ ≥ 80), the contribution of *ε*_⊥_ becomes negligible (Extended Data Figs. 6f and 8f). Furthermore, we find that *f*_r_ shifts to higher frequencies proportionally to *σ*_//_ and inversely proportionally to *ε*_//_ and is given by

6 $${f}_{{\rm{r}}}=\frac{{{\sigma }}_{//}}{2{\rm{\pi }}{{\varepsilon }}_{0}{{\varepsilon }}_{//}}.$$

This equation is independent of the device geometry and presents an equivalent of equation (4) valid at the macroscale. Therefore, once *σ*_//_ is known, *ε*_//_ can be directly obtained from *f*_r_ using equation (6), provided that *f*_r_ is well separated from *f*_c_, as in the case of our smallest channels. Also, if *α* is known, both *σ*_//_ and *ε*_//_ could be readily estimated from the two characteristic frequencies *f*_c_ and *f*_r_ without the need for simulations, simply using the equations

7 $${\sigma }_{//}=\frac{{2{\rm{\pi }}{\varepsilon }_{0}f}_{{\rm{c}}}}{\alpha },$$

8 $${\varepsilon }_{//}=\frac{{f}_{{\rm{c}}}}{\alpha {f}_{{\rm{r}}}},$$

which follow directly from equations (5) and (6). Note that the above considerations and equations (5)–(8) can also be helpful for the analysis of dielectric spectra obtained using other scanning probe approaches^44^, including those that examine higher derivatives of *|*d*C*/d*z|* and scanning impedance/microwave microscopy that directly investigates the local impedance.

### Electrical modelling

It is instructive to use an equivalent impedance circuit to describe the observed dielectric dispersion. However, because of long-range contributions from various AFM cantilever components and the complex geometry of our nanochannel devices that result in a non-uniform distribution of the electric field, an equivalent circuit should be so complicated that it is unrealistic to describe our spectra quantitatively. Below, we provide a simplified model that aims to explain the physics behind and support our numerical results (Extended Data Fig. 9). To this end, the AFM tip–nanochannel interaction can be modelled by the capacitance, *C*_tip_, that—for simplicity—accounts for both tip–air and top-hBN-layer capacitances and, to a first approximation, can be calculated as *C*_tip_ = 2π*ε*_0_*R*ln(1 + *R*(1 − sin*θ*)/(*z*_scan_ + *H*_top_/*ε*_⊥hBN_)) using the formula described in ref. ^57^. We neglect the stray capacitances associated with the AFM cantilever and consider only the tip apex capacitance that is expected to provide the dominant contribution. As for the water-filled nanochannel, we consider it as a distributed RC network shown in Extended Data Fig. 9a. It consists of two elementary RC circuits describing in-plane and out-of-plane impedances *Z*_//_(*ω*) = *R*_//_/(1 + i*ωR*_//_*C*_//_) and *Z*_⊥_(*ω*) = *R*_⊥_/(1 + i*ωR*_⊥_*C*_⊥_), respectively. *R*_⊥,//_ and *C*_⊥,//_ can be estimated as *C*_//_ = *ε*_0_*ε*_//_*wh*/Δ*l*, *R*_//_ = Δ*l*/(*σ*_//_*wh*) for the in-plane direction and *C*_⊥_ = *ε*_0_*ε*_⊥_*w*Δ*l*/*h* and *R*_⊥_ = *h*/(*σ*_⊥_*w*Δ*l*) for the out-of-plane direction, in which Δ*l* is the length of the circuit element along the channel. We also considered another capacitance *C*_b_ in series to *Z*_⊥_, to model the effect of the bottom hBN layer between the water channel and the ground. Plugging in the experimental values relevant to our devices, we can readily find that *Z*_⊥_(*ω*) ≪ 1/(*ωC*_b_) for all frequencies, meaning that the contribution of *Z*_⊥_(*ω*) is negligible in our experiments, in agreement with the above numerical and analytical calculations. The distributed network can then be simplified further and described by the electrical circuit shown in Extended Data Fig. 9b, in which the water impedance is modelled by a single RC unit in the in-plane direction, that is, *Z*_ch_(*ω*) = *R*_//_/(1 + i*ωR*_//_*C*_//_), in which *C*_//_ = *ε*_0_*ε*_//_*wh*/l*, *R*_//_ = l*/(*σ*_//_*wh*) and l* is the effective length of the nanochannel contributing to electrostatic interactions with the AFM tip. With reference to Extended Data Fig. 5d, l* notably exceeds the tip diameter and, without loss of generality, can be assumed to be on the order of several micrometres. The total equivalent impedance between the AFM tip and the ground is then given by

9 $$Z(\omega )=\frac{1+{{\rm{i}}\omega R}_{//}({C}_{//}+{C}_{{\rm{geom}}})}{{\rm{i}}\omega {C}_{{\rm{geom}}}(1+{\rm{i}}\omega {R}_{//}{C}_{//})},$$

in which *C*_geom_ = *C*_tip_*C*_b_/(*C*_tip_ + *C*_b_) is the capacitance that depends on geometric parameters but not on the electrical properties of water. Extended Data Fig. 9c shows *|Z|* as a function of *f* for the three representative devices. The effective capacitance of the modelled circuit is given by 1/*ωZ*(*ω*) and shows the same qualitative dependence on *f*, *σ* and *ε* of the capacitance gradient, *|*d*C*/d*z*|. Extended Data Fig. 9d plots *|*1/*ωZ*(*ω*)*|* that indeed exhibits both low-*f* and high-*f* plateaus characterized by frequencies *f*_c_ and *f*_r_, in good agreement with the experiment and numerical simulations. Despite its simplicity, the electrical model also reproduces well the changes in the high-*f* plateau with varying *ε*_//_ of water (Extended Data Fig. 9f) and changes in *f*_c_ with varying *σ*_//_ (Extended Data Fig. 9e).

Using this model, we can also corroborate the expressions for *f*_c_ and *f*_r_ given by equations (5) and (6). Indeed, the pole of equation (9) yields the relaxation frequency as *f*_r_ = 1/(2π*R*_//_*C*_//_) and, plugging in *R*_//_ and *C*_//_ in terms of *σ*_//_ and *ε*_//_, results in equation (6). The zero of equation (9) yields the cut-off frequency so that *f*_c_ ≅ 1/(2π*R*_//_*C*_geom_), for which we take into account that *C*_geom_ is larger than *C*_//_. Accordingly, *f*_c_ is proportional to *σ*_//_ ∝ 1/*R*_//_ and depends on the measurement geometry (through *C*_tip_ and *C*_b_) but is independent of *ε*_//_, in agreement with our numerical simulations. Expressing *C*_tip_, *C*_b_ and *R*_//_ in terms of geometric and electrical parameters as defined above and taking *C*_b_ = *ε*_0_*ε*_hBN_wl*/*H*, we obtain equation (5), in which, in the case of our geometry, the geometric factor *α* can be approximated to *α* ≅ (*hw*/l*)(1/(2π*R*) + *H*/(*ε*_hBN_wl*)). Using the specific parameters for our representative devices (*h* ≈ 30, 5 and 1.5 nm) and the effective channel length l* = 3 μm, this yields *α* of about 4 × 10^−3^, 1 × 10^−3^ and 2 × 10^−4^, respectively, in reasonable agreement with the numerically simulated values of *α*. Thus, equations (7) and (8) with *α* calculated analytically, as obtained from the simplified electrical model shown in Extended Data Fig. 9b, also allow for semi-quantitative estimates of *σ*_//_ and *ε*_//_, which are found to differ by only a factor of less than 2 from the values extracted through our more quantitative, numerical analysis.

### Alternative phenomenological Debye-like MW analysis

For completeness, we demonstrate that applying the MW formalism typically used for macroscale dielectric spectra^62–64^ would yield the same *ε*_//_ and *σ*_//_ values as reported in Fig. 3, although in a more convoluted way. In this alternative framework, the anisotropic, complex dielectric function in equation (1) for the water slab is replaced with the following expression:

10 $${\varepsilon }_{\perp ,//}^{* }(\omega )={\varepsilon }_{0}\left({\varepsilon }_{{\rm{h}}f\perp ,//}+\frac{{\varepsilon }_{{\rm{l}}f\perp ,//}-{\varepsilon }_{{\rm{h}}f\perp ,//}}{1+{\rm{i}}\omega {\tau }_{\perp ,//}}\right)-{\rm{i}}\frac{{\sigma }_{\perp ,//}}{\omega },$$

in which *τ*_⊥_ and *τ*_//_ are characteristic Debye-type relaxation times and *ε*_l*f*⊥_ (*ε*_h*f*⊥_) and *ε*_l*f*//_ (*ε*_h*f*//_) are the low-*f* (high-*f*) dielectric constants of the water slab, respectively, in each direction. No broadening parameters are required, as a single, ideal Debye-type relaxation describes our spectra. We omit the explicit conductivity term and further simplify equation (10) to:

11 $${\varepsilon }_{\perp ,//}^{* }(\omega )={\varepsilon }_{0}\left({\varepsilon }_{{\rm{h}}f\perp ,//}+\frac{{\varepsilon }_{{\rm{l}}f\perp ,//}-{\varepsilon }_{{\rm{h}}f\perp ,//}}{1+{\rm{i}}\omega {\tau }_{\perp ,//}}\right),$$

because the Debye term in equation (10) inherently accounts for conductivity contributions at finite frequencies. This is evident by rewriting equation (11) in terms of its real and imaginary parts:

12 $${\varepsilon }_{\perp ,//}^{* }(\omega )={\varepsilon }_{0}\left({\varepsilon }_{{\rm{h}}f\perp ,//}+\frac{{\varepsilon }_{{\rm{l}}f\perp ,//}-{\varepsilon }_{{\rm{h}}f\perp ,//}}{1+{\omega }^{2}{{\tau }_{\perp ,//}}^{2}}\right)-{\rm{i}}\left(\frac{({\varepsilon }_{{\rm{l}}f\perp ,//}-{\varepsilon }_{{\rm{h}}f\perp ,//})\omega {\tau }_{\perp ,//}}{1+{\omega }^{2}{{\tau }_{\perp ,//}}^{2}}\right).$$

**Extended Data Fig. 11:**
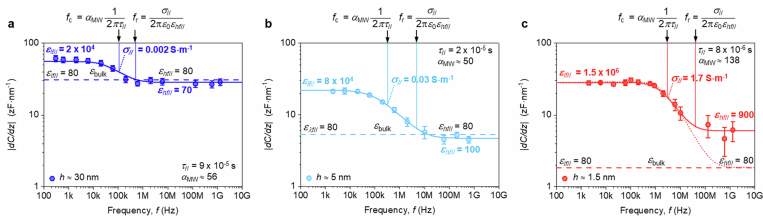
Fitting experimental spectra with a Debye-type approximation. **a**, Experimental data as a function of frequency (symbols) and corresponding fits (solid line) using the Debye-type MW relaxation model in equation (11) and 3D numerical simulations for our representative device with *h* = 30 nm. Bars are the random noise level as measured at each *f*. Fitted parameters: *τ*_//_ = 9 × 10^−5^, *ε*_l*f*//_ = 2 × 10^4^ (yielding *σ*_//_ = 0.002 S m^−1^ using *σ*_//_ = *ε*_0_*ε*_l*f*//_/*τ*_//_) and *ε*_h*f*//_ = 70. Dashed line shows the model prediction for high-*f* and low-*f* dielectric constants equal to the value of bulk water (*ε*_l*f*//_ = *ε*_h*f*//_ = *ε*_l*f*⊥_ = *ε*_h*f*⊥_ = *ε*_bulk_). **b**, Same as in **a** but for our representative device with *h* = 5 nm. Fitted parameters: *τ*_//_ = 2 × 10^−5^, *ε*_l*f*//_ = 8 × 10^4^ (yielding *σ*_//_ ≈ 0.03 S m^−1^) and *ε*_h*f*//_ = 100. **c**, Same as in **a** and **b** but our representative device with *h* = 1.5 nm. Fitted parameters: *τ*_//_ = 8 × 10^−6^, *ε*_l*f*//_ = 1.5 × 10^6^ (yielding *σ*_//_ = 1.7 S m^−1^) and *ε*_h*f*//_ = 900. Dotted red line is the best fitting at low frequencies but imposed high-*f* dielectric constant equal to the bulk value (*ε*_h*f*//_ = 80), demonstrating that, for *h* = 1.5 nm, the experimental data at high frequencies lie well above the response expected from bulk water. Other experimental parameters used in the simulations: geometric parameters and out-of-plane dielectric constants of water (*ε*_l*f*⊥_ = *ε*_h*f*⊥_) are as used in Extended Data Fig. 10. Source data

This equation reproduces the observed spectral behaviour. However, because the imaginary part vanishes as *ω* → 0, fitting the observed low-*f* plateau requires assuming unrealistically large in-plane *ε*_l*f*//_, as usually happens in the MW analysis of macroscale systems^68,70^. Extended Data Fig. 11 shows results from implementing equation (10) in our 3D numerical simulations and fitting simulated *|*d*C*/d*z|* to the experimental spectra for our representative nanochannels with thicknesses *h* = 30, 5 and 1.5 nm. Consistent with the fittings in Extended Data Fig. 10, the simulated spectra are largely insensitive to *ε*_⊥_ or *σ*_⊥_ and the fits yield the same in-plane dielectric constants at high *f* as our model, that is, *ε*_//_ = *ε*_h*f*//_ (Extended Data Fig. 10 and Fig. 3a). They correspond to intrinsic dielectric constants of water, extracted beyond the MW relaxation regime (≫10 MHz), in which the conductivity of water no longer dominates. This shows that the extracted *ε*_//_ are independent of the specific analysis used. At low *f*, however, the fit using equation (10) yields very large dielectric constants (*ε*_l*f*//_ ≈ 10^4^–10^6^) up to the cut-off frequency *f*_c_. These values are not physical dielectric constants and should not be mistaken with the values of *ε*_//_ reported in Fig. 3a. They simply reflect the in-plane DC conductivity of the water, which—in this formalism—can be estimated as *σ*_//_ = *ε*_0_*ε*_l*f*//_/*τ*_//_, yielding the same *σ*_//_ values as obtained from equation (1) (Extended Data Fig. 10 and Fig. 3b). Alternatively, using the fitted high-*f* dielectric constant, *ε*_h*f*//_, and the relaxation frequency, which—in this approximation—is given by *f*_r_ = *σ*_//_/(2π*ε*_0_*ε*_h*f*//_), we again recover the same *σ*_//_ values.

Although this phenomenological approximation ultimately gives identical *ε*_//_ and *σ*_//_ to our model, it has notable drawbacks. First, as discussed above, it yields unphysically large *ε*_l*f*//_ that have no physical meaning. Second, unlike macroscale measurements, in which the MW approximation can be fitted directly to the measured effective dielectric function, our nanoscale spectra still require full-3D numerical simulations to account for the system geometry, such as the AFM tip geometry, the scan height and the nanochannels structure. Moreover, in this framework, extracting *σ*_//_ depends on either *ε*_l*f*//_ or *ε*_h*f*//_, both of which can only be determined from 3D numerical fitting of the low-*f* or high-*f* plateaus, respectively. By contrast, in our model, *σ*_//_ is directly proportional to the cut-off frequency *f*_c_ through the geometric factor *α* (equation (5)), which can be estimated analytically, allowing extraction of *σ*_//_ without further numerical simulations.

We emphasize that, despite similarities, there are also key differences between our local spectra and those measured at the macroscale. Therefore, caution is needed when applying the MW approximation to our local measurements. First, several combinations of *ε*_l*f*//_ and *τ*_//_ can fit the low-*f* plateau equally well, because neither parameter has physical meaning. In fact, in our local spectra, the amplitude of the low-*f* plateau is set by the geometric capacitances in series with the channels—determined primarily by the AFM tip size, the channel thickness and its distance from the bottom gate—rather than by the conductivity of water. As a result, higher values of *σ*_//_ do not lead to higher low-*f* plateaus in our spectra, as we might naively expect. Instead, it only shifts the MW relaxation to higher frequencies, as described by equation (5). We also note that, in our ‘first-principles’ model, the complex dielectric constants defined in equation (1) diverge at zero frequency for a finite conductivity, but this divergence is truncated by the system geometry directly treated in the modelling, producing a Debye-like plateau independent of the *ε*_//_ or *σ*_//_ of water. Finally, the characteristic frequency 1/(2π*τ*_//_) of the Debye-like relaxation in equation (10) does not coincide with the cut-off frequency *f*_c_. Again, this is because, in our local measurements, *f*_c_ depends on the system geometry. In the MW formalism, *f*_c_ = *α*_MW_/(2π*τ*_//_), in which *α*_MW_ is a different system-dependent parameter that is not purely geometric but related to the purely geometric parameter *α* introduced in our model by *α*_MW_ = *αε*_l*f*//_, in which *ε*_l*f*//_ is extracted from numerically fitting the low-*f* plateau. These observations highlight that, although the Debye-like MW approximation ultimately produces the same results as those obtained using equations (1) and (5)–(8), it is less straightforward, depends more heavily on simulations and introduces parameters without physical meaning.

## Online content

Any methods, additional references, Nature Portfolio reporting summaries, source data, extended data, supplementary information, acknowledgements, peer review information; details of author contributions and competing interests; and statements of data and code availability are available at 10.1038/s41586-025-09558-y.

## Supplementary information

Peer Review File

## Source data

Source Data Fig. 1

Source Data Fig. 2

Source Data Fig. 3

Source Data Extended Data Fig. 2

Source Data Extended Data Fig. 3

Source Data Extended Data Fig. 5

Source Data Extended Data Fig. 6

Source Data Extended Data Fig. 7

Source Data Extended Data Fig. 8

Source Data Extended Data Fig. 9

Source Data Extended Data Fig. 10

Source Data Extended Data Fig. 11

## Data Availability

Source data of all figures and Extended Data Figs. that support the findings of this study are available with this paper and at https://zenodo.org/records/16883291 as a .txt file^71^. All other data are available from L.F. on reasonable request.
