# Supplementary material for: In-plane dielectric constant and conductivity of confined water
Source: Nature. 2025 Oct 15;646(8085):606–10. doi: 10.1038/s41586-025-09558-y (PMC12527945; doi:10.1038/s41586-025-09558-y)
Supplement: Supplementary file 1 — Peer Review File [file 41586_2025_9558_MOESM1_ESM.pdf]

nature portfolio

Peer Review File

In-plane dielectric constant and conductivity of confined water

Corresponding Author: Dr Laura Fumagalli

Any redactions in this file are there to maintain patient confidentiality, the confidentiality of unpublished data, or to remove third-party material.

This file contains all reviewer reports in order by version, followed by all author rebuttals in order by version.

Version 0:

Reviewer comments:

Referee #2

(Remarks to the Author)
This is a very nice paper that reports scanning dielectric microscopy studies to probe the in-plane dielectric properties of water confined between flat surfaces down to 1 nm confinement thickness. For nano-confined water, the in-plane dielectric constant reaches giant values whereas the conductivity approaches superionic values. This behavior is interpreted in terms of the disordered water hydrogen-bonding network, which facilitates water in-plane polarization as well as fast proton motion. The work is sound and the paper is well written, the methods are novel and the results are relevant for the field of nano-confined liquids, I recommend publication and only have a few small comments on the presentation.

If I understand correctly, the measured frequency-dependent polarization response is split into the dielectric part and the conductivity part in Eq. 1 by use of the zero-frequency (static) conductivity sigma, which removes the zero-frequency divergence of the polarization response. This is a standard procedure both experimentally and theoretically. My question concerns the interpretation of the dielectric part. According to the classical linear response theory of electrolytes (Theoretical calculation of ionic solution properties, J. M. Caillol, D. Levesque, and J. J. Weis, J. Chem. Phys. 85, 6645 (1986)), the dielectric part contains ionic contributions, in fact it has a contribution due to ion-water correlations and due to ion-ion correlations. In particular the ion-water contribution has been controversially discussed in literature, by simulations (Dissecting ion-specific dielectric spectra of sodium-halide solutions into solvation water and ionic contributions, Rinne et al., The Journal of Chemical Physics 141, 214502 (2014)) it has been shown that the water-ion as well as ion-ion contributions contribute non-negligibly to the zero-frequency dielectric constant of salt solutions. So the decomposition of the polarization response of electrolytes into a water part (dielectric) and an ionic part (conductivity) is not strict, not even for bulk electrolytes. At interfaces there seems to be even more cross-talk between these two material properties. I would suggest to briefly mention the fact that the splitting into dielectric and conductivity contributions is heuristic and the interesting contribution of ion-water correlations to the dielectric response.

Minor comments:
The second sentence of the abstract talks about interfacial water, I think the authors mean confined water here?
Likewise, when water conductivity is first mentioned in the abstract and in the introduction, it would be good to already state here whether proton or salt conductivity is meant.

Referee #3

(Remarks to the Author)
In this paper, Fumagalli, Geim et al. describe electrostatic force microscopy-based measurements of the in-plane dielectric constant of confided water. To this end, they used water-filled nanochannels in hexagonal boron nitrate – a system they have used in their famous previous study to measure the out-of-plane dielectric constant.

The key change for the current study is to change the geometry to have the electrostatic field dominated by the in-plane dielectric component, and to extend the frequency range of the dielectric measurements to the MHz and GHz regime to

compensate for the ionic in-plane conductivity. The key finding is a spectacularly high in-plane dielectric constant as well as a high lateral conductivity for a channel height of less than a few nanometers. The findings are indeed fascinating because interfacial water is still poorly understood, yet relevant for many fields. Due to the fundamental nature and the general relevance of interfacial effects in water, I would support a publication in Nature if the following comments are addressed.

I think the authors should make clear from the start that the giant in-plane dielectric constant they report is induced by the confinement, rather than being a general effect seen in any form of interfacial water that they were only able visualize in confined water.

The entire story relies heavily on the statement that the sensitivity of the capacitance gradient signal to the in-plane dielectric function inside the cannels increases with increasing height of the hBN spacer between channel and bottom electrode. I think the simulations show that this is indeed the case. Nevertheless, I wonder how the authors chose the specific spacer thickness for the experiments. Can the transition between “sensitive to perpendicular epsilon” to “sensitive to in-plane epsilon” be seen in the simulations (e.g. Ext. Fig 7c)? I guess there is a tradeoff between sensitivity to in-plane epsilon and overall strength of the dC/dz signal. I think discussing these points in more detail would strengthen the story significantly.

The strong increase of the in-plane conductivity in the channels is probably connected to the electrostatic double layer (EDL) that forms at the water-hBN interface. Here I wonder, how much the specific surface chemistry between the hBN and the slightly acidic CO2-saturated water would influence the results. The authors mention specific effects between the hBN and the water molecules on proton conductivity. This raises the question: How general are these observations or is the giant dielectric constant on hBN a specific effect of this particular system? Under strong confinement, the EDL would probably only consist of the surface charge and a Stern layer of adsorbed or strongly confined ions. I therefore wonder if the results reported by the authors could also provide further insights into the physics of the EDL.

The methods of AFM-based dielectric spectroscopy as used in this work are well established through several publications by Dr. Fumagalli’s own group and many others. A critical step in the acquisition of the data is the calibration step described at the end of the Local broadband dielectric imaging and spectroscopy chapter on page 6. Both the drive signal and the detected force signal are subject to frequency dependent transfer gains through the signal cables, contact points and through the “background” dielectric properties of the hBN substrate. The authors address this by making a reference dC/dz measurement as function of the vertical tip position on the spacer region and normalizing the signals with respect to a reference low frequency curve. First of all, I think it would be helpful to discuss the ideas behind this calibration for the non-expert readers in more detail. Secondly, the authors simply state that they “scaled” the curves to match the reference curve. For me, scaling means that the data is multiplied by a scaling factor. Looking at the raw curves in Ext. data 3d, I get the suspicion that the scaling is slightly more complex: For example, comparing the yellow 600 MHz curve to the low frequency curves or the 1.1 GHz curve, there is at least an additional offset that needs to be subtracted to obtain the scaled curves in the big graphs. What is the physics behind these calibration steps? Another issue I have with the calibration procedure is that the geometry of the electric fields depends on the tip sample height. Thus, the reference curves measured on the spacers between the channels will at certain tip-sample heights also contain electrostatic information from the channels. I would find it more than helpful if the authors would describe and discuss the calibration procedure in more detail.

Finally, I wonder if the dielectric measurements could not be done with lateral electrodes applied to the surface. This would provide at least one independent measurement.

Version 1:

Reviewer comments:

Referee #2

(Remarks to the Author)
I am satisfied with the authors´ reply and recommend publication of the revised version.

Referee #4

(Remarks to the Author)
The authors report a very detailed and meticulously performed study of the dielectric properties of water at small dimensions. The work combines three key elements. The first is the nanofabricated structures that allow to create confined channels of water in the broad interval of thicknesses, and importantly spanning range from sub-nanometer to multiple nanometers. It is important to note that these channels are also atomically clean. The second is the dielectric microscopy - which in this case spans a range of techniques including direct second harmonic imaging to frequency mixing measurements. The third is the extensive electrostatic modelling. All three are SoTA, and their combination brings the dielectric measurements to the next level of quantitativeness.

The findings that authors report on the formation of the partially disordered layers with ultra high dielectric constant is extremely interesting. While speculative arguments were made towards possibiolities of such anomalies for multiple decades (the earliest example I am aware of stems from Rebinder/Schukin school of colloid chemistry), experimental evidence has been unreliable at best. I believe that the work by Fumagalli et al establishes conclusive proof of this behavior. Thsi manuscript goes well beyond their first paper on this topic (also in Nature, I believe), and definitely merits publication.

I also believe that the author methodology is compartible with much broader range of liquids, and hence can affect a broad range of applciations from carbon capture to biology and medicine.

Referee #5

(Remarks to the Author)
The manuscript reported a scanning dielectric microscopy study to probe the in-plane dielectric properties of nanoconfined water down to 1 nm. The data showed that the in-plane dielectric constant reached giant values. In addition, the conductivity peaked at a few S m-1 17, close to values characteristic of superionic liquids. Those properties were interpreted in terms of the disordered water hydrogen bonding network, which facilitated water in-plane polarization as well as fast proton motion. The manuscript is exceptionally well written. However, I have reservations about the method to measure the dielectric constant of a liquid. I cannot recommend its publication in Nature.
The recent interest on the dielectric properties of nanoconfined water was prompted by a publication from the same group (ref. 22, Fumagalli et al. , Science 360, 1339-1342 (2018)). That paper motivated several theoretical models and simulations to explain the data. Conflicting interpretations were proposed.

I note that the experiments have not been reproduced/repeated by any other group. This is surprising after 7 years. I wonder why the theoretical community has been so keen on performing simulations while the experimental community has not. By using a far less sophisticated experimental set-up and AFM method O. Teschke and co-workers measured the dielectric constant of water next to a mica surface (O. Teschke, G. Ceotto, and E. F. de Souza, Phys. Rev. E 64, 011605 (2001)).

The scientific topic is characterized by controversial interpretations and very few experimental results. I would have appreciated an independent validation of the experimental method. The authors might consider to test the method in a model system with well-defined dielectric constant values at all distances. Otherwise, this contribution might just feed a bubble of theoretical interpretations.

Referee #6

(Remarks to the Author)
In this manuscript, the authors employ scanning dielectric microscopy to investigate the in-plane dielectric properties of confined water within 200 nm-wide nanoscale fluidic channels of varying heights. These channels are fabricated using van der Waals-assembled hBN monocrystals and graphene. The primary distinction from their previous work, which examined the out-of-plane dielectric permittivity of confined water, lies in two key modifications: (i) the inclusion of a thick hBN layer between the ground electrode and the water-filled nanochannel to enable an in-plane electric field within the channel, and (ii) the utilization of significantly higher electric field frequencies, reaching the GHz regime, to capture dielectric dispersion over a broader bandwidth.
The experimental and modeling efforts behind this manuscript are of exceptionally high quality. The authors demonstrate meticulous sample preparation, conduct scanning dielectric microscopy measurements with precise calibrations, and perform in-depth data analysis using multiple modeling techniques. Their expertise in scanning dielectric microscopy is evident, as they have optimized the device configuration to achieve the desired sensitivity to in-plane dielectric properties of confined water. Additionally, they establish a rigorous calibration protocol to accurately quantify the measured capacitive gradients.
By comparing the signal from water-filled nanochannel regions with that of hBN spacers, the authors isolate the net contribution of confined water, which is proportional to the modulus of its complex dielectric constant. Their experimental findings reveal a frequency-dependent dispersion of the capacitive gradient (modulus of the complex dielectric constant) in the range of 10^5 to 10^7 Hz, varying as a function of the nanofluidic channel height. This behavior is also linked to significant differences in capacitance gradient drops.
Beyond these observations, the authors assume that the dispersion in dielectric properties around the MHz range arises solely from conductive losses, arguing that orientational polarization relaxation of water dipoles occurs only above 10 GHz. Based on this premise, they define an anisotropic complex dielectric constant in which the imaginary component accounts exclusively for losses due to sample conductivity. Using this framework, they conduct 3D numerical simulations to compute the dielectric spectra (dC/dz vs. frequency) as a function of various parameters, including bottom hBN thickness, water channel height, in-plane and out-of-plane dielectric permittivity, and conductivity. By fitting their model to the experimental data, they extract effective values for the anisotropic dielectric permittivity and conductivity as functions of water confinement dimensions, which they discuss in detail.
That fundamentally means that all the results, discussion and interpretation relies critically on the assumption that all observed losses stem from conductivity. Consequently, their conclusions regarding the substantial enhancement of in-plane water polarizability (with ε// exceeding that of bulk water by more than an order of magnitude) and the superionic-level in-plane conductivity for channel heights below 4 nm are contingent on this hypothesis. While the discussion remains internally consistent, the authors fail to consider other potential dielectric losses in the MHz range, particularly Maxwell-Wagner dispersion due to surface charge accumulation leading to space charge polarization. Accounting for this contribution could yield more realistic values for in-plane dielectric permittivity and conductivity, obviating the need to reconcile ferroelectric-like polarizability with superionic conductivity in confined water.
Maxwell-Wagner dielectric relaxation is a well-documented phenomenon in microscopically confined water within a dielectric medium of differing dielectric properties. The disparity between the dielectric properties of water and hBN can induce charge accumulation, which relaxes similarly to a rotating dipole. In confined water, molecular mobility is hindered due to hydrogen bonding with the confining walls, thereby modifying relaxation times. Consequently, interactions between

confined water and the interface are expected to introduce space charge polarization, leading to an additional low-frequency relaxation process characteristic of Maxwell-Wagner-type polarization. In narrower confinements, interface effects are amplified, enhancing interfacial polarization contributions to the dielectric spectrum.
To properly account for this effect, the complex dielectric permittivity should include an additional Debye-like term, which the authors have not considered. Moreover, this relaxation process should exhibit a decrease of the relaxation frequency upon increasing temperatures. The omission of such a contribution in their data analysis compromises the quantification of results and subsequent discussion. Practically, this oversight also implies that low-frequency permittivity could be enhanced by the water confinement while the high-frequency permittivity values could converge for all channel heights (a trend seemingly evident in Figure 2b of the manuscript). This suggests that the appropriate normalization of dC/dz should reference the 1 GHz values.
Given these concerns, I cannot recommend the manuscript for publication in its current form. Major revisions are required to incorporate an interface dipole Maxwell-Wagner-like relaxation (potentially alongside conductivity) as a source of the observed losses in the MHz range. This necessitates recalculating the 3D numerical simulations under this revised framework and modifying or omitting Extended Data Figures 5, 6, 8, 9, and 10, as well as Main Figures 2a and 3. A thoroughly revised discussion of the obtained results is also required.
Minor Comments:
• Extended Data Figure 7: Please use the same color scale for panels (a) and (b), as they convey similar types of information.
• Figures (d) and (e): Modify the color scheme to improve clarity, as the current choice is confusing.

Version 2:

Reviewer comments:

Referee #5

(Remarks to the Author)
In the previous report I suggested to validate the experimental method by measuring the dielectric contact of a liquid with a well-defined dielectric contact as a function of the tip-hBN distance. Figure 3 in their previous publication (Fumagalli et al. , Science 360, 1339-1342 (2018)) had an inconsistence. The out-plane value of the dielectric constant at 20 nm seemed to be 30. How the hBN surface could affect the organization of the water molecules 20 nm away from the surface? Furthermore, the fittings provided in that paper implied that the influence of the surface could be observed 100 nm away. For that reason, the authors should have validated the experimental method in a non-polar liquid. In this system, the dielectric constant should not depend on the distance to the surface.

Referee #6

(Remarks to the Author)
I've carefully read the reply provided by the authors and I'm very satisfied with their answers and I positively appreciate the modifications included in the text. However, I'm still concerned about the accuracy of the high values obtained for the in-plane dielectric constant for the confined values and its implications in terms of a physico-chemical interpretation of the water molecules organization, so I would like to delve deeper into the calculation process and further clarify some more specific points before recommending this manuscript for publication.
From what I understand from the manuscript and supplementary material, in their analysis, the authors isolate the contribution of the confined water to the measured capacitance gradient by subtracting the signal obtained over the hBN spacer regions from that of the water-filled nanochannels, i.e., they analyze the quantity Δ(dC/dz)|z = (dC/dz)|z, water – (dC/dz)|z, hBN measured at a fixed z (which I assume it is the same for all frequencies) and this is the data used to fit with the simulations (line 428 page 11).
• The first thing it would be important to clarify is how z is calculated/measured. At some point, the authors mention that they obtain the data by scanning at a constant high – is this a constant z-scanner position or a constant z-distance from the surface calculated from a previously measured topography (dual pass mode?). In the first case, what could be the impact of different topographical features of the hBN top layer over the water channels? When the channels are empty the topography shows a valley but I wonder if water filled channels show a significant roughness that can have an impact. This concern emerges from images EDFig2 b and c; the scale bars for these figures are showing nm and from the Figure caption it says "(b,c) Same as (a)" which is a Topography image. If this is the case, the filled channels there show a roughness of tens of nanometers… Since it doesn't make sense, it would be great for the authors to correct it. My point is that if the data under analysis is Δ(dC/dz)|z, then any error/shift in the z position where the measurements are taken for the water channels and the hBN spacers would significantly impact in the result since the dC/dz signal changes exponentially at low z, and this could perturb the calculations.
• Another potential source of systematic error in the scanning dielectric microscopy measurements presented in the manuscript arises from deflection-induced variations in the actual tip-sample distance when comparing different regions of the sample—specifically, between the water-filled nanochannels and the surrounding hBN spacers. Since the electrostatic force acting on the AFM tip is proportional to the local capacitance gradient (dC/dz), and this force in turn causes a deflection of the cantilever, the tip will experience a slightly different vertical displacement depending on the local dielectric environment. Even when the tip is nominally held at a constant height by the piezo actuator, a higher dC/dz over the water regions will induce greater attraction, subtly pulling the tip closer to the sample surface. A preliminary analysis using the manuscript data shows that the resulting shift in tip height (Δz) is likely to be on the sub-picometer to picometer scale under

shown conditions, but the steep dependence of dC/dz on z means that even such small errors can propagate into significant deviations in the extracted dielectric constant if not properly accounted for. Finite element simulations used to model the dielectric response are particularly sensitive to absolute tip-sample distance, and a 1 nm error in z can yield overestimations or underestimations of ε‖ by more than 10%. Therefore, precise control and calibration of the tip position—and, ideally, active correction for cantilever deflection effects—are essential to ensure accurate quantification of the dielectric properties of confined water and therefore, it would be great if the authors could include a more detailed explanation of the measurements protocol and z calculation.
• Finally, if I understand it correctly that in the manuscript the authors extract the dielectric response of the confined water by subtracting the capacitance gradient measured over the hBN spacer regions from that measured over the water-filled nanochannels (which can be confirmed numerically by looking at the values show in in EDFig.10, where for example, values of dC/dz of 30 zF nm-1 are shown for low f for the smaller channels), this subtraction assumes that the only difference between the two regions is the dielectric material filling the channel, while the surrounding electrostatic environment and the field distribution remain essentially unchanged.
This method relies on a critical assumption: that the electric field geometry and boundary conditions are sufficiently similar in both regions such that the difference in dC/dz arises solely from the change in dielectric properties within the nanofluidic channel. While this may be approximately valid for a static, linear dielectric (e.g., in the GHz regime where losses are negligible), it becomes problematic at lower frequencies—particularly in the MHz range—where the confined water exhibits significant in-plane conductivity or interfacial polarization. Under these conditions, the confined water cannot be treated as a simple dielectric medium. Instead, it behaves as a heterogeneous, lossy material, with free or bound charge carriers contributing to complex dielectric relaxation and conductivity effects. These charge dynamics modify the spatial distribution of the electric field within the nanochannel, redistributing it away from what would be expected in a purely capacitive system. Importantly, this field redistribution is nonlinear and spatially asymmetric—it depends strongly on the conductivity and anisotropy of the confined water, the geometry of the channel, and the frequency of the applied electric field. In contrast, the hBN spacer regions, being purely insulating and homogeneous, sustain a very different field profile as shown in ED Fig5d. Thus, subtracting Δ(dC/dz)|z = (dC/dz)|z, water –(dC/dz)|z, hBN under these conditions introduces non-physical artifacts into the signal attributed to the confined water. The resulting Δ(dC/dz) is no longer representative of the water's dielectric response alone but also includes contributions from the difference in field line geometry and energy storage in the surrounding environment. This has a direct impact on the interpretation and fitting procedure: when the authors use FEM simulations to extract ε‖ and σ‖ from the Δ(dC/dz) curves, they implicitly assume that their model reflects the true experimental field distribution. However, if the field profile in the water region is substantially altered by conduction or interfacial effects, and this is not incorporated into the model, then the simulation will converge on compensatory values of ε‖ and σ‖ to match the shape and magnitude of the experimental curves. In practice, this can lead to artificially high dielectric constants or conductivity values, as the model attempts to absorb the impact of a more complex underlying field distribution into its fitted parameters. This could explain the extraordinarily high values of ε‖ (ferroelectric-like) and σ‖ (superionic-like) reported in the manuscript for water confined below ~4 nm. Rather than indicating new physics, these values may instead reflect modeling artifacts arising from an oversimplified interpretation of the field geometry and signal decomposition. To mitigate this, the authors should consider modeling the absolute dC/dz values over both the hBN and water regions individually, using realistic frequency-dependent field distributions that account for both dielectric anisotropy and conduction. Alternatively, a full 3D simulation that includes charge transport and relaxation dynamics in the confined layer would be necessary to decouple the real physical contributions from geometric and electrostatic artifacts.
• In this regard, it would be great to add the corresponding X and Y electric potential profiles to Extended Data Fig. 5 e, to see the variation of the potential in plane along and across the nanochannels in the different conditions.
On a second order of questions, the authors use the experimental data Δ(dC/dz)|z = (dC/dz)|z, water –(dC/dz)|z, hBN obtained at z~ 18-19 nm to best fit using the 3D numerical model. I wonder how is the z-dependence of the results, i.e., are the obtained values for the fitting different when using experimental data at z~ 10 nm or z~30 nm? Could the authors also show the equivalent Figure 2 obtained from data measured at another z?

Version 3:

Reviewer comments:

Referee #2

(Remarks to the Author)
I read carefully the reports by referees 5 and 6, the authors´ reply and the revised manuscript. I must say that the reports are written in a very careful and knowledgeable fashion, the referees know are experts. All questions were clearly answered in the reply and mostly required only slight changes in the paper.

The first question by referee 5 is somewhat contradictory, validation of the method with a different liquid is not really possible because we do not know the dielectric behavior of liquids at the nanometer scale. The second question of referee 5 was correctly answered by the authors, carefully reading the 2018 paper it becomes clear that the authors realized that no long-range effect on the water dielectric properties is needed to explain the data (in agreement with simulation results by Schlaich et al, PRL 2016).

Referee 5 asks about the theoretical analysis of the measurements, which is very involved since the authors need to solve the Maxwell equations including a finite frequency-dependent conductivity, the replies of the authors are clear and the changes in the paper make the complex analysis more accessible to the reader.

With the changes I recommend publication of the paper as is.

Referee #3

(Remarks to the Author)
I reviewed an earlier version of the manuscript and I can say that all the points I have raised are now solved also thanks to the additional questions raised by the other referees.

I think reviewer #5 raised an important point by asking for experiments on other dielectrics than water. While I see the particular experimental challenge, I think this would provide valuable insights into the interpretation of the effect. The authors discuss the role of the suppression of hydrogen bonds under confinement in the context of the Grotthuss mechanism. Experiments on non-polar liquids that do not exhibit hydrogen bonds (such as low-n alkanes or other non-polar solvents) could directly show the role of the hydrogen mobility in the observed effects.

Reviewer #6 raised a couple of technical questions on the measurement procedure. The first question concerns the accuracy of the vertical tip-sample distance. The authors used a “constant height” approach, where the scanner remains at a constant vertical position during the horizontal scans. The explanations all make sense as long as the sample is oriented exactly parallel to the scanner. I assume that the authors took care of that – nevertheless it would probably be helpful to mention this, as it is crucial to understand how the constant height mode improves accuracy here. I also wonder how piezo creep affects the vertical position. Did the authors observe changes in the vertical position between two subsequent “touch downs” between scanlines?

The concern about potential z-error induced by electrostatics-induced cantilever bending seems to be taken into account by recording the deflection and applying a corresponding correction to the vertical position. Finally, additional simulations show that in the z-range used in this study, the effect of smaller z-variations on the expected outcome is negligible.

The third comment concerned the modelling of the electric field around the channels. The authors used a 3D numerical modelling, that explicitly calculated the field distribution around the channels, so as far as I understood the reviewer’s question, the issue is resolved.

When reading the manuscript and the discussion, I had one additional question: The authors used relatively high AC drive amplitudes (up to 4V). At high voltages, the response can become non-linear (e.g. due to band bending effects at the surfaces). Did you check whether the response is still within the linear regime?

Open Access This Peer Review File is licensed under a Creative Commons Attribution 4.0 International License, which permits use, sharing, adaptation, distribution and reproduction in any medium or format, as long as you give appropriate credit to the original author(s) and the source, provide a link to the Creative Commons license, and indicate if changes were made.
In cases where reviewers are anonymous, credit should be given to 'Anonymous Referee' and the source.
The images or other third party material in this Peer Review File are included in the article’s Creative Commons license, unless indicated otherwise in a credit line to the material. If material is not included in the article’s Creative Commons license and your intended use is not permitted by statutory regulation or exceeds the permitted use, you will need to obtain

permission directly from the copyright holder.
To view a copy of this license, visit https://creativecommons.org/licenses/by/4.0/

Reply to Reviewer's comments

Reply to Reviewer #2

This is a very nice paper that reports scanning dielectric microscopy studies to probe the in-plane dielectric properties of water confined between flat surfaces down to 1 nm confinement thickness. For nano-confined water, the in-plane dielectric constant reaches giant values whereas the conductivity approaches superionic values. This behavior is interpreted in terms of the disordered water hydrogen-bonding network, which facilitates water in-plane polarization as well as fast proton motion. The work is sound and the paper is well written, the methods are novel and the results are relevant for the field of nano-confined liquids, I recommend publication and only have a few small comments on the presentation.

We thank the Reviewer for the kind assessment of our work.

If I understand correctly, the measured frequency-dependent polarization response is split into the dielectric part and the conductivity part in Eq. 1 by use of the zero-frequency (static) conductivity sigma, which removes the zero-frequency divergence of the polarization response. This is a standard procedure both experimentally and theoretically. My question concerns the interpretation of the dielectric part. According to the classical linear response theory of electrolytes (Theoretical calculation of ionic solution properties, J. M. Caillol, D. Levesque, and J. J. Weis, J. Chem. Phys. 85, 6645 (1986)), the dielectric part contains ionic contributions, in fact it has a contribution due to ion-water correlations and due to ion-ion correlations. In particular the ion-water contribution has been controversially discussed in literature, by simulations (Dissecting ion-specific dielectric spectra of sodium-halide solutions into solvation water and ionic contributions, Rinne et al., The Journal of Chemical Physics 141, 214502 (2014)) it has been shown that the water-ion as well as ion-ion contributions contribute non-negligibly to the zero-frequency dielectric constant of salt solutions. So the decomposition of the polarization response of electrolytes into a water part (dielectric) and an ionic part (conductivity) is not strict, not even for bulk electrolytes. At interfaces there seems to be even more cross-talk between these two material properties. I would suggest to briefly mention the fact that the splitting into dielectric and conductivity contributions is heuristic and the interesting contribution of ion-water correlations to the dielectric response.

We appreciate this pertinent comment. We have added the suggested note together with the above two references into the data analysis section of Methods.

Minor comments:
The second sentence of the abstract talks about interfacial water, I think the authors mean confined water here?

We thank the Reviewer for pointing this out. It was an introductory statement where we tried to say that generally the electrical properties of both interfacial and confined water were not well understood. We have clarified the concerning sentence in the revised manuscript.

Likewise, when water conductivity is first mentioned in the abstract and in the introduction, it would be good to already state here whether proton or salt conductivity is meant.

We agree - now both abstract and introduction make it clear that we refer to proton conductivity.

Reply to Reviewer #3

In this paper, Fumagalli, Geim et al. describe electrostatic force microscopy-based measurements of the in-plane dielectric constant of confided water. To this end, they used water-filled nanochannels in hexagonal boron nitrate – a system they have used in their famous previous study to measure the out-of-plane dielectric constant.
The key change for the current study is to change the geometry to have the electrostatic field dominated by the in-plane dielectric component, and to extend the frequency range of the dielectric measurements to the MHz and GHz regime to compensate for the ionic in-plane conductivity. The key finding is a spectacularly high in-plane dielectric constant as well as a high lateral conductivity for a channel height of less than a few nanometers. The findings are indeed fascinating because interfacial water is still poorly understood, yet relevant for many fields. Due to the fundamental nature and the general relevance of interfacial effects in water, I would support a publication in Nature if the following comments are addressed.
We thank the Reviewer for careful reading of our manuscript and for its positive assessment.

I think the authors should make clear from the start that the giant in-plane dielectric constant they report is induced by the confinement, rather than being a general effect seen in any form of interfacial water that they were only able visualize in confined water.
We agree with the criticism. The observed giant dielectric constant is certainly not a general interfacial phenomenon but arises due to strong confinement. Our text was confusing in this respect. The problem is now rectified throughout the manuscript, starting from the abstract.

The entire story relies heavily on the statement that the sensitivity of the capacitance gradient signal to the in-plane dielectric function inside the cannels increases with increasing height of the hBN spacer between channel and bottom electrode. I think the simulations show that this is indeed the case. Nevertheless, I wonder how the authors chose the specific spacer thickness for the experiments. Can the transition between "sensitive to perpendicular epsilon" to "sensitive to in-plane epsilon" be seen in the simulations (e.g. Ext. Fig 7c)? I guess there is a tradeoff between sensitivity to in-plane epsilon and overall strength of the dC/dz signal. I think discussing these points in more detail would strengthen the story significantly.
We are grateful for this question. Indeed, in parallel with doing the reported experiments over the last 5 years, we also used simulations to determine the most suitable thickness of bottom hBN so that we could assess the in-plane epsilon and minimize contributions from the out-of-plane epsilon. As the Reviewer suggested correctly, there is a trade-off. Our analysis shows that the thickness range is relatively narrow, between 50-500 nm. For thinner bottom hBN, the *dC/dz* signal is dominated by the out-of-plane epsilon and the in-plane contributions are small or negligible. For hBN thicker than 500 nm, the signal amplitude decreases substantially, making error bars unreasonably large. We had used bottom hBN layers up to 200 nm thick. This extra limitation was dictated by fabrication requirements: Thicker hBN crystals are stiffer and provide poor adhesion, leading to delamination upon filling the channels with water. Following the Reviewer's comment, we have added this discussion about suitable hBN thicknesses to Methods (end of section Numerical modeling and data analysis, page 11), including a new figure with simulations (Extended Fig. 7a-e).

The strong increase of the in-plane conductivity in the channels is probably connected to the electrostatic double layer (EDL) that forms at the water-hBN interface. Here I wonder, how much the

specific surface chemistry between the hBN and the slightly acidic CO2-saturated water would influence the results. The authors mention specific effects between the hBN and the water molecules on proton conductivity. This raises the question: How general are these observations or is the giant dielectric constant on hBN a specific effect of this particular system? Under strong confinement, the EDL would probably only consist of the surface charge and a Stern layer of adsorbed or strongly confined ions. I therefore wonder if the results reported by the authors could also provide further insights into the physics of the EDL.

Indeed, the gradual (1/*h*) increase of the in-plane conductivity for relatively large channels is attributed to the standard surface-charge effect as, e.g., discussed in ref. 15 of our manuscript. There are no surprises in this behavior. For simplicity, we described this surface-charge effect using our 3-layer model in Fig. 3. We admit that this point was poorly explained, and the revised manuscript now mentions explicitly that the conductivity increase at relatively large *h* is due to surface charges.

For channel heights below a few nm, we observe an additional increase in conductivity by nearly two-orders of magnitude, which correlates with the sharp rise in the in-plane dielectric constant. In the continuous theory of electrolytes, this spatial scale is comparable to the thickness of the Stern and Gouy-Chapman layers, much smaller than the Debye length characterizing the entire EDL thickness. We have deliberately avoided any references to the continuous theory because, for the few-nm confinement, the discreteness of water cannot be ignored, as its structure becomes strongly layered. The increase in both in-plane epsilon and conductivity is attributed to this restructuring of nanoconfined water, which causes disruption of the hydrogen-bond network. From this perspective, we expect our observations to apply to water confined between any surfaces, not only hBN. This conclusion agrees with the theory in refs. 28,29 where simulations were carried out for nanochannels with graphene surfaces. The theory has also found giant dielectric constants and, like our manuscript, attributed the effect to both water-dipole ordering and hydrogen-bond disorder induced by the extreme confinement rather than specifics of the confining material. For technical reasons, our experiments have been limited to hBN nanochannels and, therefore, we avoided statements about applicability of our results to other surfaces. However, following the Reviewer's comment, we have revised the concluding paragraph stating that we expect the observed behavior to be rather general for nanoconfined water, independently of the nature of confining surfaces.

The methods of AFM-based dielectric spectroscopy as used in this work are well established through several publications by Dr. Fumagalli's own group and many others. A critical step in the acquisition of the data is the calibration step described at the end of the Local broadband dielectric imaging and spectroscopy chapter on page 6. Both the drive signal and the detected force signal are subject to frequency dependent transfer gains through the signal cables, contact points and through the "background" dielectric properties of the hBN substrate. The authors address this by making a reference dC/dz measurement as function of the vertical tip position on the spacer region and normalizing the signals with respect to a reference low frequency curve. First of all, I think it would be helpful to discuss the ideas behind this calibration for the non-expert readers in more detail. Secondly, the authors simply state that they "scaled" the curves to match the reference curve. For me, scaling means that the data is multiplied by a scaling factor. Looking at the raw curves in Ext. data 3d, I get the suspicion that the scaling is slightly more complex: For example, comparing the yellow 600 MHz curve to the low frequency curves or the 1.1 GHz curve, there is at least an additional offset that needs to be subtracted to obtain the scaled curves in the big graphs. What is the physics behind these calibration steps?

Indeed, calibration of the obtained signals was critical at high frequencies. We omitted a detailed explanation for brevity, because a similar calibration procedure was reported previously. As the Reviewer correctly observed, our 'scaling' involved not only the gain factor but also the offset, two parameters. The latter is always present for these types of capacitance curves, irrespective of applied frequency. In their analysis, the offset is typically just subtracted, because it does not depend on local properties (e.g., see refs. 10,57). We are sorry that we made a mental shortcut and did not mention these details previously.
We agree that the calibration procedure needs to be discussed better for the general reader. Accordingly, we have substantially expanded the section Local Broadband Imaging in Methods (page 9) and added new figures (Extended Figs. 3e,f). The revised Methods now clarify the calibration steps in detail and explain the physics behind. In brief, we obtained $|dC/dz|(z)$ curves at different frequencies and scaled them to the curve measured at our lowest frequency of 2 kHz, using gain and offset as fitting parameters. Offsets were found to be the same above water channels and away from them, above hBN spacers. This confirmed that, for all frequencies up to 1GHz, the offset originated from long-range impedance contributions between the upper part of the AFM tip/cantilever and the sample. Importantly, while the gain factor is critical to accurately evaluate the electrical properties of water from the measured signals, the offset played little role. This is because our analysis relies on relative changes in $|dC/dz|$ along the device surface, that is, between regions with and without water (between water channels and spacer areas). In addition, we verified our calibration procedures using reference devices made of hBN on silicon and obtained good agreement with the expected dielectric spectra for all frequencies (now added to the manuscript, see Extended Figs. 3e,f). Also, large channels can be considered as another reference where water is expected to exhibit bulk properties, as indeed found in the experiment. We thank the Reviewer for this thoughtful comment.

Another issue I have with the calibration procedure is that the geometry of the electric fields depends on the tip sample height. Thus, the reference curves measured on the spacers between the channels will at certain tip-sample heights also contain electrostatic information from the channels. I would find it more than helpful if the authors would describe and discuss the calibration procedure in more detail.
The Reviewer is right in the assessment that, if separation between water channels were small, calibration curves obtained above hBN spacers can contain electrostatic contributions coming from water itself. This would lead to an underestimate of the dielectric constant of water inside the channels. To avoid such crosstalk, we have specifically chosen the distance between the channels large enough. We checked that the calibration curves obtained above hBN spacers were not influenced by electrostatic effects from water inside the channels even for large tip-surface distances (hundreds of nm). To clarify this point, we have now added another figure (Extended Data Fig. 7f). It shows that within our experimental accuracy the calibration curves do not change due to the presence of water. Following the Reviewer's comment, we have added a caveat about a possibility of the electrostatic crosstalk (end of section Local broadband dielectric imaging and spectroscopy in Methods, page 11).

Finally, I wonder if the dielectric measurements could not be done with lateral electrodes applied to the surface. This would provide at least one independent measurement.
We have also been thinking about fixed electrodes for dielectric studies of nanoconfined water for a long time. It would be great, as many more groups would be able to use such a technique, employing standard capacitance bridges. However, this approach is extremely challenging. The main problems are the following. For very thin channels, only a fraction of our nanochannels gets filled with water,

and not necessarily with a uniform thickness over the whole channel length. Furthermore, there are water puddles/bridges forming between channels because of partial delamination. If extended electrodes are placed along the nanochannels, this uncertainty in water coverage would inevitably lead to large artifacts when extracting water's electrical properties. In addition, one needs to have a proper reference to compare signals coming from water with those of the known dielectric materials and to quantitatively take into account possible electrostatic stray contributions. Our SDM provides the unique advantage of killing the two birds with one stone. The technique allows us to exactly see the channels that are filled with water, measure their thickness with the required accuracy and choose channels' sections most suitable for measurements of dielectric spectra, thus minimizing possible errors. Moving the AFM tip between water channels and spacer areas provides an in-situ reference to calibrate the signals and avoid stray electrostatic effect. Unfortunately, we still cannot see any other way to measure even out-of-plane dielectric properties of water without the use of SDM, leaving aside the more complex in-plane measurements.

Reply to Reviewer's comments

Reply to Comments of Reviewer #2

I am satisfied with the authors´ reply and recommend publication of the revised version.

We are grateful for the support from the Reviewer.

Reply to Comments of Reviewer #4

The authors report a very detailed and meticulously performed study of the dielectric properties of water at small dimensions. The work combines three key elements. The first is the nanofabricated structures that allow to create confined channels of water in the broad interval of thicknesses, and importantly spanning range from sub-nanometer to multiple nanometers. It is important to note that these channels are also atomically clean. The second is the dielectric microscopy - which in this case spans a range of techniques including direct second harmonic imaging to frequency mixing measurements. The third is the extensive electrostatic modelling. All three are SoTA, and their combination brings the dielectric measurements to the next level of quantitativeness.

The findings that authors report on the formation of the partially disordered layers with ultra high dielectric constant is extremely interesting. While speculative arguments were made towards possibiolities of such anomalies for multiple decades (the earliest example I am aware of stems from Rebinder/Schukin school of colloid chemistry), experimental evidence has been unreliable at best. I believe that the work by Fumagalli et al establishes conclusive proof of this behavior. Thsi manuscript goes well beyond their first paper on this topic (also in Nature, I believe), and definitely merits publication.

I also believe that the author methodology is compartible with much broader range of liquids, and hence can affect a broad range of applciations from carbon capture to biology and medicine. am satisfied with the authors´ reply and recommend publication of the revised version.

We appreciate not only the kind words but even more the Reviewer's thorough analysis of our work. The articulation of our three methodological pillars (which the Reviewer captured so clearly) demonstrates their expert understanding of both the technical challenges we overcame and the significance of our findings. We are also encouraged by the provided historical perspective, recognizing that our work offers conclusive experimental evidence for phenomena discussed for decades but only speculatively.

Reply to Comments of Reviewer #5

The manuscript reported a scanning dielectric microscopy study to probe the in-plane dielectric properties of nanoconfined water down to 1 nm. The data showed that the in-plane dielectric constant reached giant values. In addition, the conductivity peaked at a few S·m-1 17, close to values characteristic of superionic liquids. Those properties were interpreted in terms of the disordered water hydrogen bonding network, which facilitated water in-plane polarization as well as fast proton motion. The manuscript is exceptionally well written.

We thank the Reviewer for reading our manuscript and its analysis.

However, I have reservations about the method to measure the dielectric constant of a liquid. I cannot recommend its publication in Nature.

The recent interest on the dielectric properties of nanoconfined water was prompted by a publication from the same group (ref. 22, Fumagalli et al. , Science 360, 1339-1342 (2018)). That paper motivated several theoretical models and simulations to explain the data. Conflicting interpretations were proposed.

I note that the experiments have not been reproduced/repeated by any other group. This is surprising after 7 years. I wonder why the theoretical community has been so keen on performing simulations while the experimental community has not. By using a far less sophisticated experimental set-up and AFM method O. Teschke and co-workers measured the dielectric constant of water next to a mica surface (O. Teschke, G. Ceotto, and E. F. de Souza, Phys. Rev. E 64, 011605 (2001)).

Our previous (and current) results were based on the combination of nanoscale dielectric microscopy (only a few groups worldwide have this expertise) and nanoscale capillaries made by van der Waals assembly (only 4 groups have so far published using such capillaries). The geographical overlap between these two technologies exists only in Manchester, which makes direct replication of the previous experiment difficult, especially in the absence of sufficient incentives. Indeed, the central observation from our Science 2018 paper (the suppressed polarization of water near surfaces) has been accepted for a century, and it has been confirmed directly and indirectly using various experimental techniques by many research groups. It is also worth mentioning that even the PRE 2001 paper cited by the Reviewer made essentially the same claim (very low dielectric constant for water near the surface of mica crystals), despite limited accuracy and complex modelling depending on many parameters employed in that experiment.

The significant attention our previous work received, particularly from theorists, stems primarily from it being the first rigorous experimental proof of the low dielectric constant of confined water, particularly evident when dielectric images of water and hBN of the same thickness were measured side by side and compared directly without any modelling. As for experimentalists, we believe there remains little incentive to repeat our measurements, not only because of their technical complexity but also because our findings align with the established consensus about the out-of-plane dielectric constant of interfacial water. If there were substantive doubts about our report in Science 2018, many experimental groups would be highly motivated to challenge the findings.

The scientific topic is characterized by controversial interpretations and very few experimental results.

We acknowledge that the topic has produced diverse theoretical interpretations using many models and assumptions. However, we would characterize this as healthy debates on the origin of the phenomenon rather than a controversy over its existence. In fact, most of these interpretations are molecular dynamics calculations, which inherently depend on numerous parameters and assumptions. We certainly agree with the Reviewer that additional experimental work would be valuable to resolve these debates and further validate our findings.

I would have appreciated an independent validation of the experimental method. The authors might consider to test the method in a model system with well-defined dielectric constant values at all distances. Otherwise, this contribution might just feed a bubble of theoretical interpretations.

Over the last 7 years, we have extensively tested our experimental method through measurements of dielectric constants and conductivity for different materials with nanoscale thicknesses. These control measurements, primarily technical in nature, have routinely been presented at conferences to confirm and support the accuracy and sensitivity of our dielectric microscopy methodology. Specifically, we have found that hBN maintains the same dielectric constant independently of its thickness down to 1 nm, as expected. Also, our studies of ionic in-plane conductivity of water confined between layers of vermiculite agree quantitatively with previously published measurements for macroscopic vermiculite samples (e.g., M. S. Whittingham, *Solid State Ionics* 168, 255-263 (2004)). To substantiate this point, below we include a figure summarizing our measurements of the dielectric constant of hBN at various thicknesses and of ionic conductivity between vermiculite layers. We have considered these test experiments unnecessary to include in the original manuscript, especially because reference (test) measurements on large channels were

provided and, as expected, the latter exhibited behavior of bulk water. We plan to publish our additional test experiments to support other ongoing studies where it is difficult to use the channel height as a variable. However, if the Reviewer and Editor believe it would strengthen our manuscript without overloading it, we would be happy to include these test measurements as Supplementary Information.

Redacted

Reply to Comments of Reviewer #6

In this manuscript, the authors employ scanning dielectric microscopy to investigate the in-plane dielectric properties of confined water within 200 nm-wide nanoscale fluidic channels of varying heights. These channels are fabricated using van der Waals-assembled hBN monocrystals and graphene. The primary distinction from their previous work, which examined the out-of-plane dielectric permittivity of confined water, lies in two key modifications: (i) the inclusion of a thick hBN layer between the ground electrode and the water-filled nanochannel to enable an in-plane electric field within the channel, and (ii) the utilization of significantly higher electric field frequencies, reaching the GHz regime, to capture dielectric dispersion over a broader bandwidth.

The experimental and modeling efforts behind this manuscript are of exceptionally high quality. The authors demonstrate meticulous sample preparation, conduct scanning dielectric microscopy measurements with precise calibrations, and perform in-depth data analysis using multiple modeling techniques. Their expertise

in scanning dielectric microscopy is evident, as they have optimized the device configuration to achieve the desired sensitivity to in-plane dielectric properties of confined water. Additionally, they establish a rigorous calibration protocol to accurately quantify the measured capacitive gradients.
By comparing the signal from water-filled nanochannel regions with that of hBN spacers, the authors isolate the net contribution of confined water, which is proportional to the modulus of its complex dielectric constant. Their experimental findings reveal a frequency-dependent dispersion of the capacitive gradient (modulus of the complex dielectric constant) in the range of 10^5 to 10^7 Hz, varying as a function of the nanofluidic channel height. This behavior is also linked to significant differences in capacitance gradient drops.
We are grateful for this thorough assessment and positive comments, which recognize the rigor of our experiment and modelling procedures as well as the significance of our findings.

Beyond these observations, the authors assume that the dispersion in dielectric properties around the MHz range arises solely from conductive losses, arguing that orientational polarization relaxation of water dipoles occurs only above 10 GHz. Based on this premise, they define an anisotropic complex dielectric constant in which the imaginary component accounts exclusively for losses due to sample conductivity. Using this framework, they conduct 3D numerical simulations to compute the dielectric spectra (dC/dz vs. frequency) as a function of various parameters, including bottom hBN thickness, water channel height, in-plane and out-of-plane dielectric permittivity, and conductivity. By fitting their model to the experimental data, they extract effective values for the anisotropic dielectric permittivity and conductivity as functions of water confinement dimensions, which they discuss in detail.
That fundamentally means that all the results, discussion and interpretation relies critically on the assumption that all observed losses stem from conductivity. Consequently, their conclusions regarding the substantial enhancement of in-plane water polarizability (with $\varepsilon_{/\!/}$ exceeding that of bulk water by more than an order of magnitude) and the superionic-level in-plane conductivity for channel heights below 4 nm are contingent on this hypothesis.
While the discussion remains internally consistent, the authors fail to consider other potential dielectric losses in the MHz range, particularly Maxwell-Wagner dispersion due to surface charge accumulation leading to space charge polarization. Accounting for this contribution could yield more realistic values for in-plane dielectric permittivity and conductivity, obviating the need to reconcile ferroelectric-like polarizability with superionic conductivity in confined water.
Maxwell-Wagner dielectric relaxation is a well-documented phenomenon in microscopically confined water within a dielectric medium of differing dielectric properties. The disparity between the dielectric properties of water and hBN can induce charge accumulation, which relaxes similarly to a rotating dipole. In confined water, molecular mobility is hindered due to hydrogen bonding with the confining walls, thereby modifying relaxation times. Consequently, interactions between confined water and the interface are expected to introduce space charge polarization, leading to an additional low-frequency relaxation process characteristic of Maxwell-Wagner-type polarization. In narrower confinements, interface effects are amplified, enhancing interfacial polarization contributions to the dielectric spectrum.
We appreciate the Reviewer's assessment and concerns. We believe there has been a misunderstanding stemming from differences between our local dielectric spectroscopy and the macroscale one as well as our omission to explicitly mention the Maxwell-Wagner model, although its effect has fully been accounted for in our analysis.

Indeed, the dielectric relaxation in our spectra reflects the same mechanisms as those described by Maxwell-Wagner dispersion formalism, which is commonly employed to analyze confined water within media of differing dielectric properties (e.g., colloidal suspensions or porous media, see for example Colosi

et al, Phys. Chem. Chem. Phys. 15, 20153 (2013)). We avoided this terminology in our manuscript because our approach does not require this approximation. The Maxwell-Wagner formalism was originally derived for capacitors comprising parallel dielectric slabs with finite conductivities and later generalized to describe less geometrically defined dielectric media using phenomenological coefficients. Our nanoscale capillaries represent a well-defined trilayer system similar to the original model used in the formalism's derivation. This allows us to model the physical system without a need of phenomenological approximations that are necessary in the Maxwell-Wagner analysis of complex heterogeneous systems.

Our analysis is straightforward and quite general. The studied system consists of two hBN slabs with well-established properties and a water layer of the known thickness, which properties are to be extracted. We modelled each slab using the most general (textbook) definition of ε^* (Eq.1 in our manuscript):

$$\varepsilon^*_{\perp,\parallel}(\omega) = \varepsilon_0\varepsilon_{\perp,\parallel} - i\frac{\sigma_{\perp,\parallel}}{\omega}. \quad \text{(R1)}$$

In this definition, the imaginary term accounts for all possible losses, including both charge transport and dipolar relaxation. For hBN slabs, the imaginary term is absent as hBN is a wide-gap insulator. We then solved the electrostatic problem, where the detailed geometry of the system is taken into account, and fitted the measured $|dC/dz|$ dispersion with water's σ and ε as the only unknown parameters. Using the definition in Eq. (R1), Maxwell and Wagner showed that the effective capacitance of a planar layered system, where σ and ε are constant, acquires a frequency dependence similar to a Debye-type relaxation. The same occurs in our case, although 3D numerical simulations are needed to take into account the AFM tip geometry and solve the electrostatic problem.

This approach substantially differs from standard macroscale analyses of complex heterogeneous systems that require effective medium approximations, where $\varepsilon*$ is typically written as:

$$\varepsilon^*(\omega) = \sum_j \frac{\varepsilon_0\Delta\varepsilon_j}{[1+(i\omega\tau_j)^{\alpha_j}]^{\beta_j}} + \varepsilon_0\varepsilon_\infty - i\frac{\sigma_{DC}}{\omega}. \quad \text{(R2)}$$

Here the Debye-like terms phenomenologically account for different relaxation mechanisms with all imaginary components contributing to losses and altering dielectric dispersion. Such multi-parameter analyses are able to directly fit complex dielectric spectra obtained from experimental apparatus for the whole macroscale system ignoring its geometry but, without parameter constraints, can produce unphysical results. The most common is extremely high ε with colossal values >10,000 being often reported for water at low f (kHz to MHz) whereas at high f water recovers its standard ε. This analysis contradicts the fact that the dielectric materials involved typically exhibit frequency-independent ε at those frequency. In particular, bulk water remains frequency independent up to the GHz range, where dipolar relaxation effects become notable. The large values of apparent ε at low frequencies are known to be an artifact due to a dominant contribution of water's conductivity (like metals can be described by infinitely high ε because of their perfect screening of low-frequency electromagnetic waves), and for that reason this is termed Maxwell-Wagner effect.

Our analysis does not rely on phenomenological approximations and includes both conductive and dipolar losses. The only assumption we have made is that confined water continues to be described by frequency-independent σ and ε within our measurement bandwidth (1GHz), similar to bulk water. These assumptions are supported by the simple behavior of the observed dielectric spectra with two flat plateaus and the transition region in between which is described by a single Debye-like relaxation without broadening, as demonstrated below. The low-frequency plateaus can only be fitted with colossal values of $\varepsilon_{//}$, as shown in Fig. R2, which can only be explained as a result of the Maxwell-Wagner approximation due to DC conductive losses. Further support comes from the fact that the high-f plateau corresponds to bulk or larger values of $\varepsilon_{//}$ (note that the accuracy of our approach allows us to fit the absolute value of ε, not simply its

variations, as is often the case in macroscale measurements). If $\varepsilon_{//}$ were to exhibit a strong frequency dependence at intermediate or high frequencies (in addition to the one induced by DC conductivity), one would expect additional relaxation features or at least some broadening of the observed transition region leading to lower high-f values of $\varepsilon_{//}$. Instead, we consistently observe flat plateaus at high frequencies corresponding to bulk or higher $\varepsilon_{//}$ for all our devices. This rules out additional dipolar relaxations in the in-plane direction. In principle, we cannot exclude that the discussed restructuring of hydrogen bonds under confined water causes some additional, albeit relatively weak, dipolar relaxation (such as that reported by Colosi *et al, Phys. Chem. Chem. Phys.* 15, 20153 (2013)), but only in the out-of-plane direction and in our largest channels. However, this is unlikely in the smallest channels, which exhibit exceptionally low dielectric constant at low frequencies (kHz), as reported in our Science 2018 study and extensively confirmed by theory. While our measurement bandwidth and accuracy was insufficient to probe this possibility in 2018, any such out-of-plane effects have no impact on the present study. Indeed, as shown in the manuscript, variations in $\varepsilon_{\perp}$ would have negligible impact on the extracted in-plane conductivity and dielectric constant. Accordingly, following the Occam Razor principle, we assumed a frequency-independent σ and ε in both directions, leaving the possibility of extra dipolar relaxations in the out-of-plane direction for future investigations.

Note that, to avoid the Maxwell-Wagner relaxation at low frequencies, we had to extend our measurements to GHz frequencies – a challenging task for nanoscale experiments which took us several years. This ensured that conductivity effects – important for the smallest capillaries even above 10 MHz – did not obscure water's intrinsic dielectric response. At sufficiently high f, all our nanochannels exhibited a frequency-independent plateau, indicating the limit $\omega \to \infty$ where losses no longer contribute to spectra and both ours and Maxwell-Wagner analyses yield exactly the same ε (see Fig. R2 below). The correctness of our analysis is also evidenced by values of ε obtained for larger channels (> 4 nm), which align well with water's bulk values.

We thank the Reviewer for this comment and acknowledge that the difference between our analysis and standard procedures used for complex media, along with our avoidance of Maxwell-Wagner terminology, may have created the impression that we neglected interfacial polarization. This is not the case. Following the Reviewer's comment, we have revised Methods and added Supplementary Information to clarify these details with appropriate references to Maxwell-Wagner relaxation in complex media and mentioning the above caveat about possible but negligible additional dipole relaxation mechanism in the out-of-plane direction (see first paragraphs of "Numerical modeling and data analysis", page 10, and "Analysis of local dielectric spectra", page 12, in Methods; and Supplementary Information, pages 1-6).

To properly account for this effect, the complex dielectric permittivity should include an additional Debye-like term, which the authors have not considered. Moreover, this relaxation process should exhibit a decrease of the relaxation frequency upon increasing temperatures. The omission of such a contribution in their data analysis compromises the quantification of results and subsequent discussion. Practically, this oversight also implies that low-frequency permittivity could be enhanced by the water confinement while the high-frequency permittivity values could converge for all channel heights …

Following the Reviewer's suggestion, we have carried out the Maxwell-Wagner analysis. To describe our dielectric spectra with a single relaxation transition, we need only one Debye-like term in Eq. R2, which leads to the following expression for the water slab:

$$\varepsilon^*_{\perp,\parallel}(\omega) = \varepsilon_0 \left(\varepsilon_{hf\perp,\parallel} + \frac{\varepsilon_{lf\perp,\parallel} - \varepsilon_{hf\perp,\parallel}}{1 + i\omega\tau_{\perp,\parallel}} \right) \tag{R3}$$

This analysis is discussed in detail in Supplementary Information and supports our original conclusions. It yields exactly the same in-plane dielectric constant and conductivity values as reported in Fig. 3 of the original manuscript.

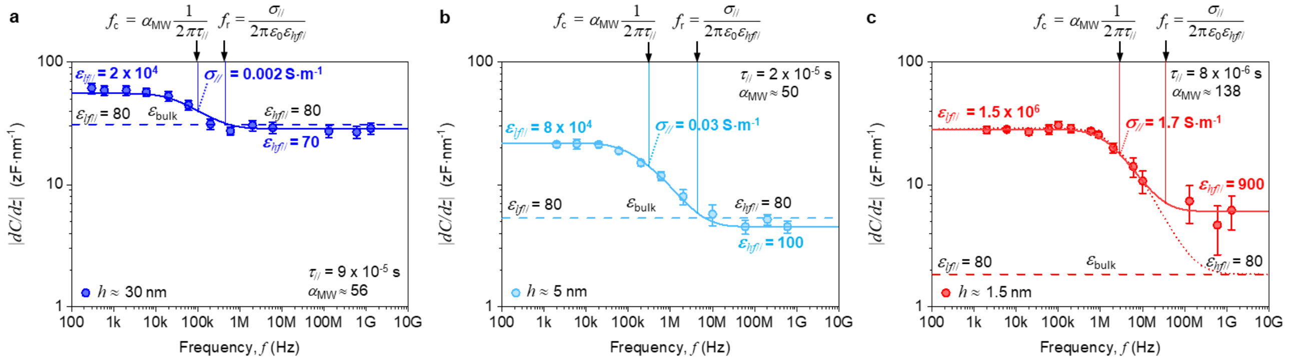

Figure R2 | Maxwell-Wagner analysis of local dielectric spectra measured for representative devices. Symbols: Experimental data without normalization. Curves: Calculated $|dC/dz|$ using 3D numerical simulations. Channel heights (**a**) h = 30 nm, (**b**) 5 nm and (**c**) 1.5 nm. Dashed lines: Bulk response $\varepsilon_{lf//} = \varepsilon_{hf//} = \varepsilon_{bulk}$.

Figure R2 above shows fits using Eq. R3 for the water slab (instead of Eq. R1) for our representative devices with thicknesses h = 30, 5, and 1.5 nm (using the same experimental data as in Extended Data Fig. 10 of the manuscript). As expected, the Debye-like model successfully fits the entire spectrum. At low frequencies, it produces apparent dielectric constants $\varepsilon_{lf//}$ reaching 10^4-10^6. Even for our relatively large channels (h = 30 nm) where water is essentially bulk, we obtained $\varepsilon_{lf//} \approx$ 20,000. As mentioned earlier, these unrealistically high values are artifacts of interpreting conductivity losses in terms of dielectric constant.

At high f, beyond the Maxwell-Wagner relaxation regime, the extracted dielectric constants in Fig. R2 are identical to those reported in Fig. 3a of the main text. This is expected because losses become negligible at high frequencies (Debye-like terms in Eqs. R2-R3 become negligible), and both analyses yield intrinsic dielectric constants. For thicker devices (h = 5 and 30 nm), $\varepsilon_{hf//}$ agrees with expected bulk water values, while the device with h = 1.5 nm shows $\varepsilon_{hf//} \approx$ 900, consistent with our original findings.

The Debye-like term in the Maxwell-Wagner model can also be rewritten in terms of conductivity so that $\sigma_{//}$ can be evaluated as the ratio of $\varepsilon_{lf//}/\tau_{//}$. To this end, note that low-frequency plateaus in Fig. R2 extend further in frequency with decreasing h, indicating shorter relaxation times $\tau_{//}$ for smaller channels. This is accompanied by the rapid increase in $\varepsilon_{lf//}$ as h decreases. Both spectral changes correspond to higher $\varepsilon_{lf//}/\tau_{//}$ and, hence, higher conductivity in smaller channels. In quantitative terms, the Maxwell-Wagner analysis yielded $\sigma_{//}$ values closely matching those in Fig. 3b of the manuscript.

It is important to emphasize that the obtained values of $\sigma_{//}$ are neither unrealistic nor challenging to explain. They agree with expectations for water thicknesses down to 2-4 nm, accounting for the known increased conductivity of water near surfaces, as discussed in the manuscript. The peak approaching superionic values at h = 1-2 nm is consistent with recent theoretical predictions for 2D confined water/ice (Kapil et al. *Nature* 609, 512–516 (2022); Jiang et al. *Nature Physics* 20, 456–464 (2024)). Similarly, the enhanced dielectric constant for highly confined water aligns with recent first-principle predictions (Motevaselian et al. *J. Phys. Chem. Lett.* 11, 10532-10537 (2020), Ruiz-Barragan et al. *Phys. Chem. Chem. Phys.* 22, 10833-10837 (2020)). Similar values and ferroelectric-like arrangements are also predicted for water inside nanotubes [e.g., Calegari Andrade et al. *J. Phys. Chem. Lett.*, 15, 6872–6879 (2024)], supporting our interpretation regarding disruption of water's H-bond network.

Finally, let us note that we have validated our modeling approach not only using channels with large h as a reference (where water is expected to be bulk-like) but also studying different experimental systems. For example, we have applied the analysis to measurements of water confined between vermiculite layers. The latter exhibits low-frequency Maxwell-Wagner relaxation similar to that observed in our nanochannels. These results (summarized in Fig. R1 above in our response to Reviewer #5) show that the extracted

conductivity values quantitatively match those previously reported for macroscopic vermiculite samples (e.g., M. S. Whittingham, *Solid State Ionics* 168, 255-263 (2004)). This additional validation further confirms the accuracy and reliability of our approach and modeling.

… while the high-frequency permittivity values could converge for all channel heights (a trend seemingly evident in Figure 2b of the manuscript).

We believe the Reviewer's interpretation of Figure 2b stems from a confusion regarding the color scales. The colors represent measured capacitive signals $|dC/dz|$, not extracted dielectric constants. The similar values (~6 zF/nm^{-1}) at GHz frequencies for different channel thicknesses (h = 5 nm and 1.5 nm) are accidental (they also depend on geometry) and actually indicate very different $\varepsilon_{//}$ values, not convergence to the bulk value. This is because the measured signal strength depends strongly on water thickness, h. If the dielectric constant in 1.5 nm-thick channels remained bulk-like and unaffected by such a strong confinement, we would expect a substantially smaller $|dC/dz|$ compared to 5 nm channels. This relationship is demonstrated by the theoretical curves (dashed lines) in Figs. 2a, Extended Data Fig. 6b and 8b, which show that the measured signal decreases roughly proportionally to h. This dependence reflects the fact that the interaction of an AFM tip with a water layer is proportional to its thickness. Therefore, the same color scale for $|dC/dz|$ in Fig. 2b provides direct experimental evidence that $\varepsilon_{//}$ is significantly enhanced in our smallest channels. This conclusion is qualitative and independent of a specific modeling approach—both our original analysis and the Maxwell-Wagner approach suggested by the Reviewer yield the same result.

We have added clarification on this point in the revised manuscript (see Fig. 2 caption and first paragraph of "Dielectric dispersion of nanoconfined water", page 3).

This suggests that the appropriate normalization of dC/dz should reference the 1 GHz values.

We accept that certain aspects of our normalization procedures were not clearly explained in our original manuscript. To clarify, normalizing the spectra using either the high- or low-frequency plateau does not affect the extracted values for ε and σ. The data fitting was performed using absolute values of $|dC/dz|$ reported in Extended Data Fig. 10, and the normalization in Fig. 2a is only for clearer presentation, to avoid stretching the y-axis and minimize the impact of variations of the AFM tips when comparing different experiments.

While it would also be possible to normalize the measured signal to 1 GHz frequency values as the Reviewer suggested, this approach is less optimal. First, as explained above, the permittivities do not converge to the same high-frequency values for all channel heights and, in addition, the high-frequency signal $|dC/dz|$ depends on channel thickness h, making it awkward as a reference point when comparing different dielectric properties and thicknesses. In contrast, at low frequencies (kHz range), the measured signals become effectively independent of the electrical properties of water and its thickness (see Extended Data Figs. 6a and 8a). They do not increase with water's ε and σ (see Extended Data Figs. 6e,f and 8e,f), and this is a key difference between our local spectra and macroscale dispersions. This occurs because in our case the low-frequency plateau reflects the geometric capacitances of the AFM tip and hBN layers in series with the channel. In this regime, the electric potential is effectively "screened" by water's conductivity along the channel length (as visualized in Fig. S5b-e), and the signal is primarily determined by the AFM tip radius and its distance from the channel, so normalizing with respect to the low-frequency value, as we did, effectively scales out these geometric factors. This makes plots using the low-frequency normalization more informative, enabling more direct comparison of water's electrical response across different devices.

Following the Reviewer's comment, we have added this clarification to Methods (see added paragraph at the end of the section "Dielectric image and dielectric spectra acquisition", page 10) and further discussed the differences between local and macroscale spectroscopy in Supplementary Information (page 5).

Given these concerns, I cannot recommend the manuscript for publication in its current form. Major revisions are required to incorporate an interface dipole Maxwell-Wagner-like relaxation (potentially alongside conductivity) as a source of the observed losses in the MHz range. This necessitates recalculating the 3D numerical simulations under this revised framework and modifying or omitting Extended Data Figures 5, 6, 8, 9, and 10, as well as Main Figures 2a and 3. A thoroughly revised discussion of the obtained results is also required.

We hope that our detailed responses and additional 3D numerical simulations using the suggested Maxwell-Wagner framework have addressed the Reviewer's concerns. We sincerely thank the Reviewer for raising all these important points, which have ultimately helped to strengthen our manuscript. As demonstrated, our results and conclusions remain valid regardless on the modelling approach used. Our figures remain valid and, in particular, Extended Data Figs 5-10 are essential for illustrating the key differences between our local spectra and standard macroscale ones, as further discussed now in Supplementary Information. To summarize, our "first-principle" approach and the Maxwell-Wagner formalism are fundamentally based on the same physics principles—they simply represent different paths to the same destination. For our well-defined trilayer system, our direct solution is more straightforward and transparent, while the Maxwell-Wagner approximation takes a more circuitous route, along which additional approximations and unphysical parameters have to be introduced.

Minor Comments:

• Extended Data Figure 7: Please use the same color scale for panels (a) and (b), as they convey similar types of information.

• Figures (d) and (e): Modify the color scheme to improve clarity, as the current choice is confusing.

We thank the Reviewer for these helpful suggestions regarding the figures' color schemes. We have modified Extended Data Figure 7 to use consistent color scales for panels (a) and (b) and have updated the color schemes in Figures (d) and (e) to improve clarity. These changes helped to further improve our manuscript. Appreciated.

Reply to Reviewer's comments

Reply to Comments of Reviewer #5

In the previous report I suggested to validate the experimental method by measuring the dielectric contact of a liquid with a well-defined dielectric contact as a function of the tip-hBN distance.

We have extensively validated our method through multiple approaches. For macroscopically-thick liquids with well-defined dielectric constants, our AFM-based method accurately reproduces their established values. Regarding confined liquids, it is important to note that all liquids organize into layered structures at the nanoscale. Measuring properties of such nanoscale liquids poses extreme challenges for any technique, and one cannot simply assume that dielectric properties remain unchanged if the thickness approaches the molecular scale. This is precisely why our AFM method provides valuable insights into a subject that remains largely inaccessible to other techniques.

In our previous response, we described two (among several) approaches that were used to validate our technique. First, we tested hBN crystals of various nanoscale thicknesses. As a non-polar solid with well-characterized theoretical properties, hBN served as an excellent reference system, whereas in terms of methodology it makes no difference whether it is a liquid or a solid. Our measurements agree well with theoretical predictions for hBN down to monolayer thickness, confirming our technique's reliability. Second, we measured water confined between mica layers, a system conceptually similar to our water capillaries but where macroscopic samples were available and studied by standard dielectric spectroscopy. Our AFM-based measurements showed good agreement with the macroscopic results, further validating our approach.

Figure 3 in their previous publication (Fumagalli et al. , Science 360, 1339-1342 (2018)) had an inconsistence. The out-plane value of the dielectric constant at 20 nm seemed to be 30. How the hBN surface could affect the organization of the water molecules 20 nm away from the surface? Furthermore, the fittings provided in that paper implied that the influence of the surface could be observed 100 nm away. For that reason, the authors should have validated the experimental method in a non-polar liquid. In this system, the dielectric constant should not depend on the distance to the surface.

There is no inconsistency in our 2018 paper. We agree completely that hBN cannot affect water molecules 20 nm away from the surface. However, our paper did not suggest this. As clearly shown in the inset of Figure 3 and explained in the text, the experimental behavior is fully and consistently explained by a model where only 0.6 nm (few layers) of non-polarizable water exists near the hBN surface, while the remaining water retains the bulk properties. This model accurately reproduces our experimental data and is physically reasonable, reflecting the limited spatial extent of surface effects on a water structure.

Reply to Comments of Reviewer #6

I've carefully read the reply provided by the authors and I'm very satisfied with their answers and I positively appreciate the modifications included in the text.

We thank the reviewer for the positive assessment of our answers and revised manuscript.

However, I'm still concerned about the accuracy of the high values obtained for the in-plane dielectric constant for the confined values and its implications in terms of a physico-chemical interpretation of the water molecules organization, so I would like to delve deeper into the calculation process and further clarify some more specific points before recommending this manuscript for publication.

From what I understand from the manuscript and supplementary material, in their analysis, the authors isolate the contribution of the confined water to the measured capacitance gradient by subtracting the

signal obtained over the hBN spacer regions from that of the water-filled nanochannels, i.e., they analyze the quantity Δ(dC/dz)|z = (dC/dz)|z, water –(dC/dz)|z, hBN measured at a fixed z (which I assume it is the same for all frequencies) and this is the data used to fit with the simulations (line 428 page 11).

• The first thing it would be important to clarify is how z is calculated/measured. At some point, the authors mention that they obtain the data by scanning at a constant high – is this a constant z-scanner position or a constant z-distance from the surface calculated from a previously measured topography (dual pass mode?). In the first case, what could be the impact of different topographical features of the hBN top layer over the water channels? When the channels are empty the topography shows a valley but I wonder if water filled channels show a significant roughness that can have an impact. This concern emerges from images EDFig2 b and c; the scale bars for these figures are showing nm and from the Figure caption it says "(b,c) Same as (a)" which is a Topography image. If this is the case, the filled channels there show a roughness of tens of nanometers… Since it doesn't make sense, it would be great for the authors to correct it. My point is that if the data under analysis is Δ(dC/dz)|z, then any error/shift in the z position where the measurements are taken for the water channels and the hBN spacers would significantly impact in the result since the dC/dz signal changes exponentially at low z, and this could perturb the calculations.

We thank the Reviewer for this comment. We acknowledge that the topography of our devices and EDFigs 2b&c may not have been sufficiently well explained in the previous manuscript.

To clarify: Our channels were intentionally fabricated with nanoscale sagging in their top surface by using a relatively thin top hBN layer. When water filled these channels, this sagging disappeared, and the surface became atomically flat with residual roughness of < 3Å. This design serves to indicate that water has successfully filled the channels (as some might be blocked and should then be ignored). We mention this procedure in the Device fabrication section with reference to our Science 2018 paper where we firstly introduced it and demonstrate it here in EDFigs. 2e,f&g. Those images were taken before and after water filling using the standard topography images in dynamic (tapping) mode that relies on short-range van der Waals forces between the AFM tip and substrate (with no voltage bias applied). One can see how the initial sagging disappears after water filling.

EDFigs. 2b&c, which caused confusion, illustrate a different effect we used to confirm water filling and should not be confused with topography images. While using the same topography imaging mode as described above and in EDFig. 2a, we intentionally applied a large low-frequency voltage between the tip and graphite bottom electrode (8 V at 1 kHz, as stated in the caption). This induced a strong electrostatic force acting on the AFM tip due the high conductivity of water in the in-plane direction (as revealed in the dielectric spectra), overwhelming the short-range van der Waals forces. As the tip scanned across the channels, the AFM feedback system adjusted the z-scanner to compensate for changes in electrostatic force, resulting in an apparent height contrast of several nanometers (shown in EDFig. 2c). This method (not reported previously, as it relies on the electrical response of water in the in-plane direction) was used only to assess water filling, not during actual dielectric measurements.

We acknowledge that using the same red/blue color scale for EDFigs. 2b&c as for our dielectric images may have contributed to the confusion. We chose these colors intentionally to distinguish these images from the true topography (given in gray scale) and to emphasize that the contrast appears due to the different electrical properties of water and hBN.

The manuscript has now been revised to clarify these technical details and to prevent any misunderstanding (with changes in Methods, section "Device fabrication", and the caption of EDFig. 2). No corrections are required in EDFigs. 2b&c or their scale bars. Given the atomically flat surface of our devices after water filling, no significant error is introduced in the z-position of the AFM tip that could affect our dielectric measurements/calculations.

Regarding other possible concerns about topography effects or errors in the tip scan height z, we feel it could be helpful to elaborate further on our methodology here. We employed a constant-height dual-pass mode, developed in earlier scanning dielectric microscopy works (see, e.g., *Nano Lett.* 2009 & *Nat. Mater.* 2012; referred to accordingly in the manuscript). This approach was specifically introduced to ensure precise control and accurate measurement of the scan height *z*, which is essential for reliable quantitative dielectric measurements at the nanoscale—not only in this study, but in general.

In this mode, the first pass acquires topography without an applied field, avoiding electrostatic force contributions. In the second pass, a relatively small AC field is applied and dielectric data are recorded at a fixed *z*-scanner position. This differs significantly from the standard "lift mode" (commonly used in electrostatic force microscopy and scanning capacitance microscopy, and likely referred to here by the Reviewer), where the tip retraces the topography profile but at a higher scan height. While the lift mode helps avoid van der Waals forces, it can also introduce substantial artifacts from variable stray capacitance due to the involved height increase. Also, determining the actual scan height becomes complicated because of tapping distance and DC electrostatic forces.

Our constant-height dual-pass mode overcomes these limitations. We directly determine the scan height by acquiring DC and AC deflection approach curves at scan image (or line) edges. The DC deflection curve allows locating the surface with sub-nanometer accuracy by effectively "touching down" the tip from the scan height, while the AC deflection curve provides *dC/dz* as a function of *z*, offering independent validation of the height in addition to the DC deflection curve, allowing correction of possible z-scanner drift. By matching the latter with the *dC/dz* scan line (as shown in EDFigs 3b&c), the actual scan height can be obtained with sub-nm precision (Fumagalli et al., *Nat. Mater.* 2012). Because the second-pass scan maintains the same fixed height, we eliminate potential artifacts from the z-scanner motion that would otherwise be present in the standard lift mode.

The described methodology ensures that our measurements of dielectric properties are truly quantitative and not compromised by topographical variations or scan height inconsistencies.

• Another potential source of systematic error in the scanning dielectric microscopy measurements presented in the manuscript arises from deflection-induced variations in the actual tip-sample distance when comparing different regions of the sample—specifically, between the water-filled nanochannels and the surrounding hBN spacers. Since the electrostatic force acting on the AFM tip is proportional to the local capacitance gradient (dC/dz), and this force in turn causes a deflection of the cantilever, the tip will experience a slightly different vertical displacement depending on the local dielectric environment. Even when the tip is nominally held at a constant height by the piezo actuator, a higher dC/dz over the water regions will induce greater attraction, subtly pulling the tip closer to the sample surface. A preliminary analysis using the manuscript data shows that the resulting shift in tip height (Δz) is likely to be on the sub-picometer to picometer scale under shown conditions, but the steep dependence of dC/dz on z means that even such small errors can propagate into significant deviations in the extracted dielectric constant if not properly accounted for. Finite element simulations used to model the dielectric response are particularly sensitive to absolute tip-sample distance, and a 1 nm error in z can yield overestimations or underestimations of ε∥ by more than 10%. Therefore, precise control and calibration of the tip position—and, ideally, active correction for cantilever deflection effects—are essential to ensure accurate quantification of the dielectric properties of confined water and therefore, it would be great if the authors could include a more detailed explanation of the measurements protocol and z calculation.

We thank the Reviewer for this thoughtful comment regarding potential systematic errors caused by cantilever deflection. The Reviewer correctly recognizes that electrostatic forces can cause variable

deflection as the AFM tip scans across regions with different dielectric properties. However, it is not a problem in our case. First of all, our measurement protocol specifically accounts for this effect. Indeed, the constant-height dual-pass mode simultaneously records both DC and AC deflection during the second pass, along with DC and AC approach curves at scan line edges. This allows us to directly measure any variation in static deflection across the scan line and, if significant, apply corrections to the z-value obtained from approach curves. Second, for the measurements reported in this study, we experimentally found that variations in DC deflection over the water channels were negligible, that is, within the picometer range, as the Reviewer correctly estimated. These variations were much smaller than the experimental errors in *z* due to *z*-scanner drifts. The latter uncertainty, which we estimated to be approximately ±1 nm, and the electrical noise level are what actually limited our accuracy in extracting the dielectric constant.

Importantly, we did not simply assumed *z* to be constant through the whole spectrum. To minimize possible errors, we measured the scan height value *z* for each frequency. By doing that, we found that the scanner drift can become significant (several nm) during day-long measurements required to acquire the whole dielectric spectrum. Therefore, for maximum possible accuracy, we specifically acquired the data in a way to maintain the same scan height *z* across all frequencies. To this end, our procedure was refined in this study by acquiring *dC/dz* scan lines at different *z*-values (not a single z value) for each frequency, as described in EDFigs. 3a-d. This approach allowed us to reconstruct the spectra at constant *z* within ±1 nm accuracy.

It is also important to emphasize that, despite the steep dependence of *dC/dz* on *z*, even the 1-nm experimental uncertainty has little impact on the extracted dielectric constants. This is because our analysis relies on measuring the difference in *dC/dz* between water channels and hBN spacers at the same z. This differential approach makes our measurements robust against small variations in absolute z. To illustrate this robustness, Fig. R1a shows the simulated and experimental spectra for our smallest representative device (1.5 nm) shown in Fig. 2 and Fig. 10c. The figure displays calculated curves for the best-fit z-value (red; corresponding to the best-fit curve in EDFig. 10c) and for z ±1 nm (orange and pink). The three curves nearly overlap, with experimental data points scattered around them, reflecting the stated ±1 nm experimental uncertainty. The 30% error we report for the extracted dielectric constants incorporates this uncertainty, along with the standard deviation of data points on the high-frequency plateau.

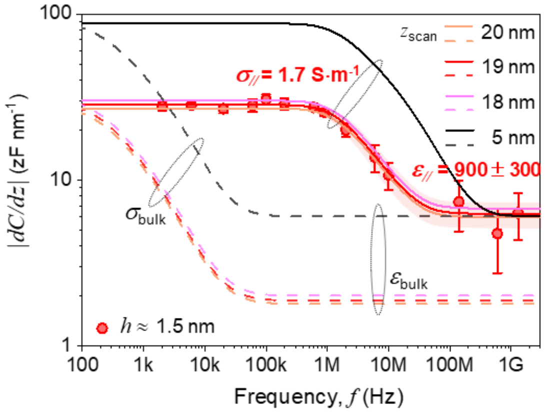

Figure R1 | Impact of the scan height uncertainty in the fitting of experimental data. (a) Symbols are the experimental data of our smallest device Extended Data Fig. 10 (and in Fig. 2 before normalization). Curves are calculated values using our full 3D numerical simulations. The red solid curve is the best fit with water's $\varepsilon_{//}$ and $\sigma_{//}$ as the only fitting parameters (labels) and the experimentally estimated scan height z_{scan} = 19 nm (other experimental parameters are the same as in Extended Data Fig. 10). The light-shaded regions indicate the uncertainty in the extracted values of $\varepsilon_{//}$ and $\sigma_{//}$. The red dashed curves: 3D numerical simulations for the same device assuming bulk water's properties (ε_{bulk} = 80, σ_{bulk} = 2 x 10^{-4} S·m^{-1}). The pink and orange curves are corresponding calculated values for z_{scan} = 18 and 20 nm, respectively, corresponding to our experimentally estimated uncertainty on the scan height of ± 1 nm. The black curves are the 3D simulations for bulk water's dielectric constant (ε_{bulk} = 80) using the scan height z_{scan} as fitting parameter to match the high-frequency plateau, yielding z_{scan} = 5 nm, with $\sigma_{//}$ = σ_{bulk} = 2 x 10^{-4} S·m^{-1} (black dashed line) and $\sigma_{//}$ = 1.7 S·m^{-1} (black solid line).

Finally, we note that we can confidently rule out that the reported large in-plane dielectric constant was due to errors in the scan height z based on several independent *qualitative* observations:

- First, the consistency of small scatter in our data across the entire spectrum provides strong validation. Each point in our dielectric spectrum comes from independent measurements at different frequencies, all requiring accurate *z* determination. If our *z* values were significantly mistaken, we would not observe such consistently flat plateaus at both low and high frequencies, nor recover the expected Debye-like relaxation across the entire spectrum.
- Second, our measurements on thicker channels accurately recover the known dielectric constant of bulk water, validating our methodology. Since we applied identical procedures to all devices with similarly flat surfaces, there is no reason why our *z* determination would be less accurate for thinner channels.
- Third, the low-frequency plateau in our spectra provides yet another crucial experimental verification. This plateau results from screening effects due to water's in-plane conductivity and, while its absolute value is independent of channel thickness and water's dielectric properties, it is highly sensitive to *z* (as explained in the manuscript and our previous reply). The excellent agreement in their absolute values between our measured low-frequency *dC/dz* values and full 3D simulations (see EDFig. 10) confirms the accuracy of our analysis.

To illustrate why alternative explanations using smaller dielectric constants fail for water inside our smallest channels, Fig. R1a shows simulations where water's $\varepsilon_{//}$ is fixed to its bulk value (black lines). While the high-frequency plateau could theoretically be reproduced by assuming a much smaller scan height (approximately 5 nm), such a simulation completely fails to reproduce the low-frequency plateau, with huge discrepancies of several tens of zF/nm. We also note that, first, such a large error in *z* determination (approximately 15 nm) is physically impossible given the high precision of modern AFM scanners. Second, such small scan heights (and all scan heights below 10-15 nm) are physically inaccessible in our constant-height mode due to tip collapse onto the surface from long-range attractive electrostatic forces under our experimental conditions (room temperature, applied voltage, soft cantilevers, and large AFM tip radii). The minimum scan heights at which we could safely operate were between 15-20 nm, as expected for these experimental conditions, which is the reason why we measured the spectra at those heights. These considerations definitively rule out such significant errors in *z* determination.

To summarize, we appreciate the Reviewer's important point about the scan height precision. However, as demonstrated above, we employed specific protocols to ensure accurate z-determination and, in addition, have strong experimental evidence confirming the reliability of our approach. Our analysis shows that potential *z*-uncertainties cannot possibly explain the enhanced dielectric constant observed in our smallest water channels. We have added these clarifications to the revised manuscript (Methods, sections 'Dielectric images and dielectric spectra acquisition' and 'Numerical modeling and data analysis'), including the figure presented here (added as EDFig. 7h) along with the sensitivity curve (*dC/dz* vs $\varepsilon_{//}$) for 1.5 nm-channels (added as EDFig. 7g), which illustrate the explanations.

• Finally, if I understand it correctly that in the manuscript the authors extract the dielectric response of the confined water by subtracting the capacitance gradient measured over the hBN spacer regions from that measured over the water-filled nanochannels (which can be confirmed numerically by looking at the values show in in EDFig.10, where for example, values of dC/dz of 30 zF nm-1 are shown for low f for the smaller channels), this subtraction assumes that the only difference between the two regions is the dielectric material filling the channel, while the surrounding electrostatic environment and the field distribution remain essentially unchanged.

This method relies on a critical assumption: that the electric field geometry and boundary conditions are sufficiently similar in both regions such that the difference in dC/dz arises solely from the change in dielectric properties within the nanofluidic channel. While this may be approximately valid for a static, linear dielectric (e.g., in the GHz regime where losses are negligible), it becomes problematic at lower frequencies—particularly in the MHz range—where the confined water exhibits significant in-plane conductivity or interfacial polarization. Under these conditions, the confined water cannot be treated as a simple dielectric medium. Instead, it behaves as a heterogeneous, lossy material, with free or bound charge carriers contributing to complex dielectric relaxation and conductivity effects. These charge dynamics modify the spatial distribution of the electric field within the nanochannel, redistributing it away from what would be expected in a purely capacitive system. Importantly, this field redistribution is nonlinear and spatially asymmetric—it depends strongly on the conductivity and anisotropy of the confined water, the geometry of the channel, and the frequency of the applied electric field. In contrast, the hBN spacer regions, being purely insulating and homogeneous, sustain a very different field profile as shown in ED Fig5d. Thus, subtracting $\Delta(dC/dz)|z = (dC/dz)|z$, water $-(dC/dz)|z$, hBN under these conditions introduces non-physical artifacts into the signal attributed to the confined water. The resulting $\Delta(dC/dz)$ is no longer representative of the water's dielectric response alone but also includes contributions from the difference in field line geometry and energy storage in the surrounding environment. This has a direct impact on the interpretation and fitting procedure: when the authors use FEM simulations to extract $\varepsilon_{/\!/}$ and $\sigma_{/\!/}$ from the $\Delta(dC/dz)$ curves, they implicitly assume that their model reflects the true experimental field distribution. However, if the field profile in the water region is substantially altered by conduction or interfacial effects, and this is not incorporated into the model, then the simulation will converge on compensatory values of $\varepsilon_{/\!/}$ and $\sigma_{/\!/}$ to match the shape and magnitude of the experimental curves. In practice, this can lead to artificially high dielectric constants or conductivity values, as the model attempts to absorb the impact of a more complex underlying field distribution into its fitted parameters. This could explain the extraordinarily high values of $\varepsilon_{/\!/}$ (ferroelectric-like) and $\sigma_{/\!/}$ (superionic-like) reported in the manuscript for water confined below ~4 nm. Rather than indicating new physics, these values may instead reflect modeling artifacts arising from an oversimplified interpretation of the field geometry and signal decomposition. To mitigate this, the authors should consider modeling the absolute dC/dz values over both the hBN and water regions individually, using realistic frequency-dependent field distributions that account for both dielectric anisotropy and conduction. Alternatively, a full 3D simulation that includes charge transport and relaxation dynamics in the confined layer would be necessary to decouple the real physical contributions from geometric and electrostatic artifacts.

We fully agree with the Reviewer that treating water as a purely dielectric (non-conducting) liquid can lead to significant artifacts at low frequencies because of incorrect field distribution assumptions. However, our analysis specifically accounts for water's high conductivity, its geometry and the resulting changes in field distribution. In fact, we have implemented exactly what the Reviewer suggested above: full 3D numerical simulations that account for the actual geometry and dimensions of the system, and water's conductive and dielectric properties.

The Reviewer's concern appears to stem from different models presented in the manuscript that may have caused some confusion. To clarify: All experimental spectra were analyzed using comprehensive 3D numerical modelling that treats water as a conductive medium with anisotropic properties. The simplified analytical model also presented in the manuscript—which indeed assumes the only difference between channel and hBN regions is in their dielectric constants—was never used to fit experimental data. Its purpose is purely qualitative/illustrative, to provide physical insight into spectral behavior that is relatively independent of details of experimental geometry. While this simplified model has obvious limitations, it

offers readers an accessible understanding of the underlying physics without requiring mastery of the complex 3D simulations we developed and used.

Our full 3D simulations make no simplifying assumptions and compute absolute *dC/dz* values while fully accounting for confined water's conductive and dielectric properties as well as their anisotropy, thereby eliminating the potential geometric and electrostatic artifacts the Reviewer describes. The only simplifications involved were the reduction in channels' length and the AFM probe's dimensions (cone height and cantilever length) to a few microns to manage computational costs. As discussed in the manuscript, we verified that these simplifications have no impact on our results, as the tip-sample interaction is essentially localized under the AFM tip apex and in nearby regions. Furthermore, each channel device (more than 10 in total were studied in detail) was individually modeled using its actual dimensions (spacer and channel width/height), along with the individually measured radius for each AFM tip used. We did not rely on nominal values. This meticulous modeling approach was undertaken specifically to obtain the most realistic electric field distribution between the AFM tip and confined water, ensuring that the electrical properties of confined water were extracted with minimal approximations. Also, our simulations computed absolute *dC/dz* values at various positions across the device in all three dimensions (*X,Y,Z*), replicating the experimental acquisition process at constant height. To fit the measured *dC/dz* variation between water channel and hBN spacer, we simulated the tip over each region separately in absolute values and then subtracted the resulting values—exactly matching our experimental data processing. This approach has ensured that no artifacts or systematic errors are introduced by the subtraction process.

Following the Reviewer's comment, the revised manuscript clarifies the different roles and aspects of the discussed two models which should help avoid any confusion.

• In this regard, it would be great to add the corresponding X and Y electric potential profiles to Extended Data Fig. 5 e, to see the variation of the potential in plane along and across the nanochannels in the different conditions.

Following the Reviewer's suggestion, we have added electric field profiles in the X and Y directions to EDFig. 5, complementing the Z-direction profile already provided in EDFig. 5e in the previous manuscript.

We'd like to expand on an important point regarding how changes in water conductivity affect our analysis. What matters most is the impact of these changes not on the electric potential distribution but on the *dC/dz* dependence above the hBN spacer—the quantity we measured, analyzed and subtracted to quantify dielectric properties. In EDFig. 7f, we previously demonstrated for the case of *h* = 5 nm that our calibration curves taken above the hBN spacer do not noticeably change with either frequency or water filling. To verify this remains true for our smallest channels (*h* = 1.5 nm), where we observed the most significant enhancement of dielectric constant and for which potential variations were simulated in EDFig. 5, we have added to the manuscript a new figure analogous to EDFig. 7f but for the *h* = 1.5 nm case. This figure (see EDFig. 5b in the revised manuscript) shows representative 3D simulations of the *absolute* value of *dC/dz* measured above the center of the hBN spacer at different frequencies (ranging from 100 Hz to 1 GHz) and compares water-filled versus empty channels as a function of scan height *z*. All curves practically overlap, with only minor shifts (~0.1 zF/nm) for scans close to the surface. These shifts are an order of magnitude smaller than our experimental resolution of ~1 zF/nm. While the shift increases slightly at the lowest frequencies due to water's in-plane conductivity contributions, it remains well within experimental uncertainty. We emphasize that, despite their minimal impact, these small variations were fully accounted for in our analysis. Nonetheless, this figure demonstrates that even if water's conductivity were not accounted for (for example, if using the simplified analytical model), the error in determining water's dielectric properties would be negligible, especially at GHz frequencies where water conductivity is less important and we observed the enhanced dielectric constant.

We hope this further addresses the Reviewer's concerns regarding our analysis and modeling approach, which was commended by Reviewer #4 as latest state-of-the-art modeling. We have renumbered the figures panels in EDFig. 5, added the additional X and Y potential profiles in EDFig. 5g&h and the calculated absolute values of *dC/dz* on the hBN spacer in EDFig. 5b and corresponding explanation in Methods, end of section 'Numerical modeling and data analysis'.

On a second order of questions, the authors use the experimental data Δ(dC/dz)|z = (dC/dz)|z, water – (dC/dz)|z, hBN obtained at z~ 18-19 nm to best fit using the 3D numerical model. I wonder how is the z-dependence of the results, i.e., are the obtained values for the fitting different when using experimental data at z~ 10 nm or z~30 nm? Could the authors also show the equivalent Figure 2 obtained from data measured at another z?

We thank the Reviewer for this question. The spectra shown in Fig. 2 were taken at 19 ± 1 nm from the surface, which we found to be within the optimal distance range for our measurements, providing maximum signal while ensuring measurement stability. Measurements at smaller heights (around 10 nm as suggested by the Reviewer) were impossible in our experiments. As explained above, when operating in the constant-height mode (with the AFM feedback switched off), the tip cannot be safely positioned too close to the surface due to long-range electrostatic forces that cause the tip to collapse. Under our experimental conditions, this collapse typically occurred at *z* of 10-15 nm from the surface. Therefore, we reconstructed and analysed our spectra at the nearest possible distances (15-20 nm), which maximized signal strength while maintaining a safe margin from the tip collapse. Conversely, we avoided higher *z* of 20-30 nm because the *dC/dz* signal decreased rapidly with distance, which would have significantly compromised our measurement accuracy. This consideration was particularly important for the smallest channels, where the signal-to-noise ratio already presented a challenge even at our chosen optimal distances. This can be seen in EDFigs. 6g &h of the revised manuscript, where we show full 3D numerical simulations as a function of scan height z for our smallest channels. The plots confirm that the spectral shape remains unchanged as a function of the scan height and only the signal amplitude varies. Therefore, the range of 15-20 nm represents a compromise that balances maximum signal strength with measurement stability and safety against the AFM tip collapse with the applied voltages. Following the Reviewer's comment, we have added this explanation and EDFigs. 6g&h to revised section 'Dielectric images and dielectric spectra acquisition'.

Finally, it might be helpful to provide context about the reported dielectric properties of confined water and their significance. As noted in the manuscript, our results for the intermediate confinement regime (> 4 nm) align well with previous experiments and theoretical expectations. The values obtained for the strongest confinement (< 2-3 nm) qualitatively agree with practically all theoretical predictions in the literature, where significant enhancement of both in-plane conductivity and dielectric constant is anticipated. Some theoretical studies have even predicted quantitatively similar enhancements to those we observed, as discussed in our previous reply. These values are also physically reasonable. As water becomes strongly structured near solid interfaces, it would be rather surprising to observe bulk-like dielectric properties, rather than strong changes. Since this interfacial structuring occurs universally, we expect the qualitative behavior observed here to be general across various confining systems, not limited to hBN. However, because water's molecular arrangements and dipole orientation are highly sensitive to surface charge density and polarity, quantitative values of $\varepsilon_{//}$ and $\sigma_{//}$ likely depend on surface chemistry. For example, recent predictions suggest that $\varepsilon_{//}$ can range from bulk-like to giant values (100-1,000 range) depending on surface polarity (see, e.g., Loche et al., J. Phys. Chem. B, 2020). While we acknowledge experimental limitations affecting our accuracy (approximately 30%), these do not undermine our central finding: both water's ε and σ in the in-plane direction are large near the surface and become notably enhanced under strong confinement. Our results

provide unique insights into the electrical properties of interfacial and strongly confined water that have remained experimentally inaccessible until now. Crucially, we demonstrate a viable measurement method that can be further refined in future work. To address the Reviewer's valuable comments, we have added this clarification in the revised Discussion & Conclusion sections to better contextualize our findings.

List of revisions in the resubmitted manuscript

Main text

- Pg. 3: added definition of error bars in Fig. 2a caption.
- End of 'Discussion' and 'Conclusion' section, pg. 6: added clarifications to better contextualize our findings and their significance.

Methods

- section "Device fabrication", pg. 9: extended explanation to clarify EDFig. 2b&c.
- section "Dielectric images and dielectric spectra acquisition" pgs. 10-11: improved explanation of how the constant-height mode works and how the scan height is controlled and measured; added clarification that any potential additional electrostatic force is measured by recording DC deflection and only yields negligible variations; added clarification that the estimated 1-nm uncertainly has also negligible impact, with reference to added Extended Data Figs. 7g,h. Added clarification regarding our choice of scan height and why other scan heights were experimentally impossible, with reference to added Extended Data Figs. 6g,h; added clarification about the spectral values, their 'absolute' character and our differential analysis with respect to the hBN spacer that makes our analysis robust against systematic errors and uncertainties.
- Section "Numerical modeling and data analysis", top of pg.12, end of pg.13 and top of pg. 14: added clarification that we used full 3D numerical calculations to fit the experimental data and they account for actual detailed geometry and electrical properties of the system; added paragraph explaining why our differential analysis relative to the hBN region does not introduce systematic errors and account for most realistic electric field distribution; added paragraph clarifying the impact of scan height uncertainty in the extracted values and explaining why the results obtained cannot be explained by a systematic error with that; added paragraph clarifying why our differential analysis relative to the hBN region does not introduce systematic errors associated to small electric field variations, with reference to added Extended Data Figs. 5b&g&h.
- Section "Analytical modelling", top of pg. 14: added clarification on the purpose of this simplified model and its use in the manuscript as opposed to the full 3D numerical modelling.
- Section "Analysis of local dielectric spectra", top of pg. 17: added paragraph pointing out differences between macroscale spectra and our local spectra; added the alternative phenomenological model as Eq. (9) and related main details, with reference to added Extended Data Fig. 11.

Extended Data Figures:

- Extended Data Fig. 2, pg. 22: added clarification in the caption regarding figure panels b&c
- Extended Data Fig. 5, pg. 25: added electric potential profiles in X&Y directions in figure panels g&h and maximum *dC/dz* error introduced by water's electrical response and electric field variations in our differential analysis with respect to the hBN region.
- Extended Data Fig. 6, pg. 26: added calculated spectra as function of scan height for our smallest devices (5 and 1.5 nm) in figure panels g&h.
- Extended Data Fig. 7, pg. 27: added sensitivity plot to $\varepsilon_{//}$ for 1.5 nm devices in figure panel g and 3D simulations for the experimental data in Fig. 10c in figure panel h showing the impact of the scan height.
- Extended Data Fig. 11, pg. 32: added fittings of experimental spectra with the alternative Maxwell-Wagner phenomenological model.

Reply to Reviewer comments

Reply to Comments of Reviewer #2

I read carefully the reports by referees 5 and 6, the authors´ reply and the revised manuscript. I must say that the reports are written in a very careful and knowledgeable fashion, the referees know are experts. All questions were clearly answered in the reply and mostly required only slight changes in the paper.

The first question by referee 5 is somewhat contradictory, validation of the method with a different liquid is not really possible because we do not know the dielectric behavior of liquids at the nanometer scale. The second question of referee 5 was correctly answered by the authors, carefully reading the 2018 paper it becomes clear that the authors realized that no long-range effect on the water dielectric properties is needed to explain the data (in agreement with simulation results by Schlaich et al, PRL 2016).

Referee 5 asks about the theoretical analysis of the measurements, which is very involved since the authors need to solve the Maxwell equations including a finite frequency-dependent conductivity, the replies of the authors are clear and the changes in the paper make the complex analysis more accessible to the reader.

With the changes I recommend publication of the paper as is.

We are grateful to the Reviewer for taking the time to evaluate the comments of Reviewers #5 and #6 and for the positive assessment of our replies. We particularly appreciate that the Reviewer took the additional time to read our earlier paper (Science 2018) and confirmed the validity of our previous measurements and analysis regarding the out-of-plane dielectric constant of confined water.

Reply to Comments of Reviewer #3

I reviewed an earlier version of the manuscript and I can say that all the points I have raised are now solved also thanks to the additional questions raised by the other referees.

We thank Reviewer #3 for taking the time to evaluate our manuscript again and for the continued support of our work.

I think reviewer #5 raised an important point by asking for experiments on other dielectrics than water. While I see the particular experimental challenge, I think this would provide valuable insights into the interpretation of the effect. The authors discuss the role of the suppression of hydrogen bonds under confinement in the context of the Grotthuss mechanism. Experiments on non-polar liquids that do not exhibit hydrogen bonds (such as low-n alkanes or other non-polar solvents) could directly show the role of the hydrogen mobility in the observed effects.

We appreciate this suggestion and fully agree that experiments on other liquid dielectrics could provide valuable insights and help with more detailed interpretation. From a technical perspective, in additional to bulk water reference channels, we carefully carried out control experiments using other dielectrics (atomically-thin solids). These have clearly validated the accuracy of our approach. In addition, experiments on nanoconfined liquids present significant challenges, as this Reviewer clearly appreciates. As previously noted in our reply to Reviewer #5, all liquids, including the suggested low-n alkanes, are expected to exhibit an anomalous dielectric response with increasing confinement (see, e.g., Motevaselian et al. ACS Nano 2020, doi: 10.1021/acsnano.0c03173). Therefore, their meaningful comparison with water would first require their full characterization under extreme confinement. Given the complexity of such experiments, particularly the device fabrication and the several years it took us to explore nanoconfined water, the characterization of other liquids is expected to be equally demanding. We intend to move in this direction in the near future. However, at present, expanding the work to additional liquid systems would be unrealistic and beyond its scope and reasonable timeline.

Reviewer #6 raised a couple of technical questions on the measurement procedure.

We are grateful for Reviewer #3's thorough evaluation and positive assessment of our responses to Reviewer #6's technical questions. We particularly appreciate this independent confirmation that our experimental methodology adequately addresses the technical concerns raised previously.

The first question concerns the accuracy of the vertical tip-sample distance. The authors used a "constant height" approach, where the scanner remains at a constant vertical position during the horizontal scans. The explanations all make sense as long as the sample is oriented exactly parallel to the scanner. I assume that the authors took care of that – nevertheless it would probably be helpful to mention this, as it is crucial to understand how the constant height mode improves accuracy here. I also wonder how piezo creep affects the vertical position. Did the authors observe changes in the vertical position between two subsequent "touch downs" between scanlines?

We have carefully aligned the scanner parallel to the sample plane so that the height remained constant along the horizontal direction. This can be verified directly from the reported dielectric images, which cover three (or more) parallel nanochannels. If the scanner were not properly aligned in the plane, the tip would be at different heights above different channels, causing these channels to exhibit spurious variations in the dielectric signal. Regarding piezo creep after 'touch downs', we have ensured consistent scan height by allowing sufficient settling time at the beginning of each line to reach the target height. This is also evident in the images that display the uniform signal across multiple channels.

These clarifications have been added to the revised manuscript (Methods, beginning of 'Dielectric images and dielectric spectra acquisition'). We thank the Reviewer for highlighting these important technical details.

The concern about potential z-error induced by electrostatics-induced cantilever bending seems to be taken into account by recording the deflection and applying a corresponding correction to the vertical position. Finally, additional simulations show that in the z-range used in this study, the effect of smaller z-variations on the expected outcome is negligible.

The third comment concerned the modelling of the electric field around the channels. The authors used a 3D numerical modelling, that explicitly calculated the field distribution around the channels, so as far as I understood the reviewer's question, the issue is resolved.

We appreciate the above analysis of Reviewer #6's concerns and the confirmation that our responses adequately addressed and clarified the issues.

When reading the manuscript and the discussion, I had one additional question: The authors used relatively high AC drive amplitudes (up to 4V). At high voltages, the response can become non-linear (e.g. due to band bending effects at the surfaces). Did you check whether the response is still within the linear regime?

We thank the Reviewer for this important question. Despite the relatively large AC amplitude used in the experiments, the response remained within the linear regime. This was verified through systematic measurements over a range of amplitudes, confirming that the chosen AC value did not affect the outcome. The linearity was explicitly checked in previous studies using the same approach (see, e.g., Fumagalli et al Nat. Mater. 11, 808, 2012) and is further validated in the present work by our ability to reproduce known bulk water properties when measuring relatively large channels as a reference. We added this clarification to the revised manuscript (Methods, section 'Local broadband dielectric imaging and spectroscopy').
